# CRISPR-based environmental detection of *Burkholderia pseudomallei* identifies sanitation gaps and melioidosis risk in northeast Thailand

**DOI:** 10.1038/s41467-026-73286-8

**Published:** 2026-05-15

**Authors:** Sukritpong Pakdeerat, Chalita Chomkatekaew, Phumrapee Boonklang, Raiwin Mothong, Maturada Patchsung, Arin Wongprommoon, Kesorn Angchagun, Yaowaret Dokket, Areeya Faosap, Gumphol Wongsuwan, Premjit Amornchai, Vanaporn Wuthiekanun, Jiramate Changklom, Suwatthiya Siriboon, Parinya Chamnan, Sharon J. Peacock, Julian Parkhill, Jukka Corander, Nicholas PJ Day, Nicholas R. Thomson, Chayasith Uttamapinant, Somsakul Pop Wongpalee, Claire Chewapreecha

**Affiliations:** 1 Mahidol Oxford Tropical Medicine Research Unit (MORU), Faculty of Tropical Medicine, Mahidol University, Bangkok, Thailand; 2 Department of Veterinary Medicine, University of Cambridge, Cambridge, UK; 3 Wellcome Sanger Institute, Hinxton, UK; 4 School of Biomolecular Science and Engineering, Vidyasirimedhi Institute of Science and Technology (VISTEC), Rayong, Thailand; 5 Faculty of Dentistry, Burapha University, Chonburi, Thailand; 6 Faculty of Engineering, Kasetsart University, Bangkok, Thailand; 7 Department of Infectious Medicine, Sunpasitthiprasong Regional Hospital, Ubon Ratchathani, Thailand; 8 Cardiometabolic Research Group, Department of Social Medicine, Sunpasitthiprasong Regional Hospital, Ubon Ratchathani, Thailand; 9 Faculty of Medicine, Chulalongkorn University, Bangkok, Thailand; 10 Department of Medicine, University of Cambridge, Cambridge, UK; 11 Institute of Basic Medical Science, University of Oslo, Oslo, Norway; 12 Centre for Tropical Medicine and Global Health, Nuffield Department of Medicine, University of Oxford, Oxford, UK; 13 Department of Microbiology, Faculty of Medicine, Chiangmai University, Chiang Mai, Thailand; 14 Department of Clinical Tropical Medicine, Faculty of Tropical Medicine, Mahidol University, Bangkok, Thailand

**Keywords:** CRISPR-Cas systems, Bacterial infection, Water microbiology

## Abstract

Environmental exposure to *Burkholderia pseudomallei*, the causative agent of melioidosis, remains poorly characterised due to the low sensitivity of conventional detection methods. Here, we develop CRISPR-BEEPs, a sensitive and resource-efficient CRISPR-based assay, and evaluate its performance against conventional culture-based plate inspection using double-qPCR as the reference standard. CRISPR-BEEPs demonstrated higher sensitivity (93.5% vs 19.4%) and high specificity (100% vs 98.0%). We apply the assay to water samples from natural and piped sources across 15,118 km² in northeast Thailand, collected from or near the households of 439 participants with melioidosis. We compared these with households of 190 participants with other bacterial infections and 506 healthy control participants living in the same endemic region who had never developed melioidosis. CRISPR-BEEPs detects *B. pseudomallei* in 73.3% of groundwater, 32.9% of surface water, and 26.2% of piped water samples, with results comparable to double-qPCR. The improved sensitivity reveals a significant association between environmental detection within 10 km of households and melioidosis risk (OR 2.74; 95% CI:1.38-5.48), an association undetectable using conventional methods. These findings expose critical sanitation gaps and highlight the value of high-resolution environmental surveillance for disease prevention.

## Abstract (web summary)

Detection of *Burkholderia* *pseudomallei* is challenging. Here, the authors develop a CRISPR-based assay that outperforms culture-based methods, reveal extensive *B. pseudomallei* contamination in environmental and piped water in northeast Thailand and link this to known human melioidosis cases nearby.

## Introduction

Access to clean water and adequate sanitation is fundamental to public health development^1^. The United Nations has issued Sustainable Development Goal 6 (SDG 6) to call for universal access to safe water, improved sanitation, and sustainable resource management^2^. However, in many low-resource settings, water safety remains compromised, increasing the risk of exposure to environmental pathogens that contribute to disease burdens^1,3,4^. *Burkholderia pseudomallei*, the causative agent of melioidosis, can be found in soil and water in tropical and subtropical regions, including South- and Southeast Asia, parts of Africa and the Americas, and northern Australia^5^. Human infection occurs through skin abrasions, inhalation, or ingestion and disproportionately affects individuals with underlying conditions such as diabetes or immunosuppression^6,7^. Melioidosis is a severe infection with reported case fatality rates of up to 40% in many resource-limited settings in Southeast Asia^8,9^. In contrast, mortality can be reduced to below 10% in well-resourced health systems, such as in Australia^6,7^, highlighting melioidosis as a poverty-associated disease driven by environmental exposure and health-system inequities.

Multiple outbreaks have linked *B. pseudomallei* to contaminated water supplies^10–15^. In 2023, a cluster of ten neurological melioidosis cases in southern India—eight of which were fatal—was traced to contaminated saline used in a dental clinic^11^. In 2021, an outbreak at a COVID-19 field hospital in Thailand resulted in 25 culture-confirmed cases and eight deaths, primarily among corticosteroid-treated patients^12^. The source was traced to inadequately chlorinated water used for hygiene. Similarly, outbreaks in northern^13,14^ and western^15^ Australia have been associated with unchlorinated or poorly maintained water systems, collectively resulting in 15 confirmed cases and nine deaths. Although chlorination is generally effective against *B. pseudomallei*, the bacterium can survive within biofilms and may display moderate chlorine resistance^16,17^. The World Health Organisation (WHO) Water Safety Plan framework emphasises the need for multilayered strategies to maintain effective disinfection, prevent environmental contamination, and control biofilm formation^18^. However, implementation of these measures remains challenging in many endemic regions^3,19^. Moreover, current water monitoring practices, which typically rely on faecal indicator bacteria such as *Escherichia coli*^20^, are insufficient for identifying non-faecal pathogens like *B. pseudomallei*, which can persist and replicate in water systems independently of faecal contamination.

Environmental surveillance of *B. pseudomallei* has traditionally depended on culture-based methods using selective media followed by visual inspection before confirmation of suspected colonies with biochemical, serological, or PCR assays^21–23^. While these methods have provided a foundation for detection, they suffer from low sensitivity and are increasingly challenged by competing environmental organisms resistant to antibiotics commonly used for selection, such as gentamicin and colistin, which complicates visual inspection. Although serial dilution can reduce competing flora, the unknown and highly variable concentrations of both *B. pseudomallei* and background microbes make it difficult to determine an appropriate dilution strategy. Molecular techniques such as PCR offer improved sensitivity and specificity, but their use in field settings is limited. PCR reactions are sensitive to environmental inhibitors^24,25^, require extensive protocol optimisation, and depend on specialised equipment and trained personnel. To overcome these limitations, emerging CRISPR-based diagnostics technologies – such as Cas12 and Cas13-based detection systems—show considerable promise^26,27^. When combined with isothermal amplification methods like recombinase polymerase amplification (RPA) or loop-mediated isothermal amplification (LAMP), they form powerful platforms such as SHERLOCK (Cas13 with RPA)^28–30^ and DETECTR (Cas12 with either RPA^31,32^ or LAMP^33^). These integrated systems have demonstrated high sensitivity and specificity in clinical diagnostics and are increasingly being explored for environmental surveillance^32^. Their key advantages include tolerance to common environmental inhibitors, minimal instrumentation requirements, and suitability for deployment in low-resource or field settings^34,35^.

In this study, we evaluated CRISPR-BP34, a sensitive and equipment-light CRISPR-based assay originally developed for clinical diagnostics^36,37^, for its potential use in environmental surveillance of *B. pseudomallei*. The assay is based on DETECTR^31^ technology, combining RPA with CRISPR-Cas12-mediated detection. Upon recognition of the target sequence, the Cas12 enzyme activates collateral cleavage of a reporter molecule, generating a detectable signal that can be read using a simple lateral flow dipstick, making it suitable for low-resource settings. We adapted this assay for environmental sampling by developing a laboratory workflow, termed CRISPR-BEEPs (CRISPR-BP34 with Enhanced Environmental Plate-sweep sampling). This method was deployed across 15,118 km^2^ region in northeast Thailand, an area endemic for melioidosis. Samples were collected from diverse water sources, including piped water, groundwater, and natural surface waters. In addition to validating the assay’s performance, we investigated whether individuals living in areas with higher environmental *B. pseudomallei* levels faced increased risk of melioidosis. CRISPR-BEEPs enabled a more sensitive detection and improved assessment of exposure risk, reinforcing the importance of access to clean water and calling for improved sanitation in endemic regions.

## Results

### Environmental microbial diversity in the tropics and subtropics

**Fig. 1:**
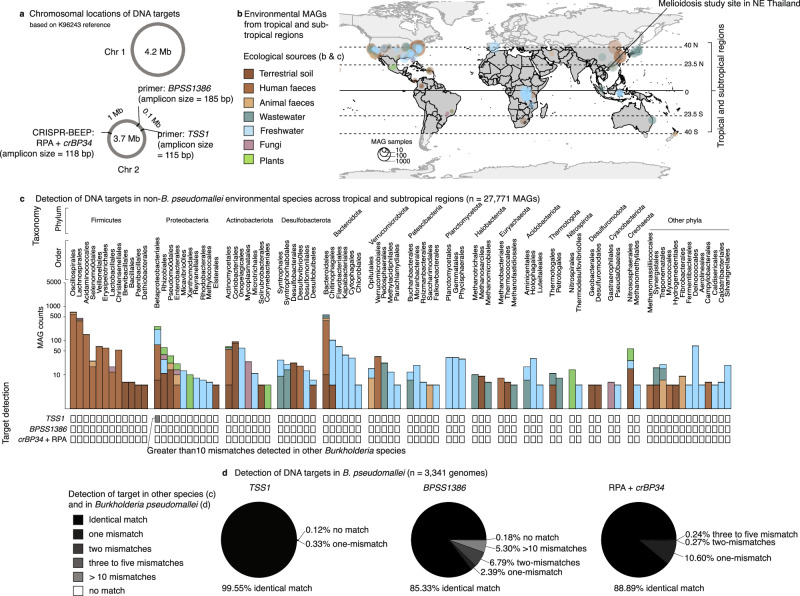
Molecular targets for *B. pseudomallei* detection and their in silico specificity and sensitivity in the context of environmental microbial background. **a** DNA targets include two qPCR primer sets (*TTS1* and *BPSS1386*) and the CRISPR-BP34 locus (RPA primers and *crBP34*), located in distinct genomic regions for independent detection. **b** Data availability of 27,771 MAGs from tropical and subtropical regions, representing microbial communities from soil, freshwater, wastewater, faeces, fungi, and plants. The top 86 taxonomic orders were shown. **c** Taxonomic abundance of environmental MAGs found in (**b**) and assessment of partial sequence matches between these taxa and the DNA targets shown in (**a**). **d** Conservation of DNA targets in the global *B. pseudomallei* population, based on 3341 high-quality genome assemblies. **b**, c MAGs are colour-coded by source. **c**, **d** Matching status is visualised using a greyscale gradient: black indicates perfect matches, grey indicates partial mismatches, and white indicates no match. Source data are provided as a Source Data file.

To adapt DNA-based detection methods—originally developed for clinical applications - for environmental surveillance, we first assessed the in silico specificity and sensitivity of selected *B. pseudomallei* DNA targets (Fig. 1a) within the context of highly diverse microbial communities found in tropical and subtropical environments. Water sources in melioidosis-endemic areas (Supplementary Fig. 1) are particularly complex^38^, containing fast-growing fungi, diverse bacterial taxa (including both fast- and slow-growing species), and other eukaryotic microbes, which pose analytical challenges for the specific and sensitive detection of *B. pseudomallei*. To characterise the microbial environment in which *B. pseudomallei* exists, we analysed metagenome-assembled genomes (MAGs) from the Earth Microbiome Project^39^—a global dataset comprising microbial communities from freshwater, wastewater, soil, plant rhizospheres, fungi, and human and animal faeces. These environments are relevant to the water systems and contamination pathways in melioidosis-endemic areas. Although the dataset does not cover all endemic countries, it provides broad represent of MAGs from tropical (23.5° N–23.5° S) and subtropical regions (23.5°–40° in both hemispheres), which overlap with previously defined melioidosis-endemic zones^5^ (Fig. 1b). This yielded a dataset of 27,771 MAGs representing 9739 operational taxonomic units (OTUs), encompassing microbial species that potentially co-exist with *B. pseudomallei* in the environment.

Among freshwater samples, 50.3% of MAGs were dominated by Proteobacteria (473/2644, 17.9%), Bacteroidota (471/2644, 17.8%), and Patescibacteria (333/2644, 12.6%). In contrast, microbial communities in faecal-contaminated and soil-associated sources were dominated by Firmicutes (2140/4467, 47.9%), Bacteroidota (775/4467, 17.3%), and Proteobacteria (417/4467, 9.3%) (Fig. 1c). The consistent presence of Proteobacteria and Bacteroidota across water, soil, faeces, and rhizosphere environments—highlights the ecological ubiquity of these phyla—Proteobacteria also include the members of the Burkholderiales order. Notably, *B. pseudomallei* was not identified in any of the MAG datasets. This is likely due to a combination of factors: (i) the bacterium’s typically low abundance in environmental samples, (ii) insufficient sequencing depth and biases in MAG assembly or taxonomic assignment, or (iii) the masking of rare *B. pseudomallei* sequences by the high-background of other microbial and archaeal DNA that varies across geographic regions.

### In silico specificity and sensitivity of *B. pseudomallei* DNA targets

The challenges described above mirror real-world difficulties in detecting *B. pseudomallei* DNA within complex environmental samples containing high-background levels of non-target microbial, archaeal, and closely related *Burkholderia spp*. DNA. To evaluate assay performed in such conditions, we assessed the in silico specificity and sensitivity of three molecular targets: *TTS1*^40^ and *BPSS1386*^41^ (used in qPCR reference test), and the CRISPR-BP34 target region, which includes the guide RNA *crBP34* and its associated RPA primers^36^ (Fig. 1a and Supplementary Table 1). When mapped against the 27,771 environmental MAGs, the CRISPR-BP34 target region and *BPSS1386* qPCR primers showed no sequence matches, indicating high environmental specificity (Fig. 1c). In contrast, *TTS1* primers exhibited partial matches to other *Burkholderia* species, with more than ten mismatches—most critically at the 3’ end of the primer, where mismatches are known to compromise PCR efficiency^42,43^. Nonetheless, all three targets are sufficiently specific to *B. pseudomallei* in the context of available MAG data, with a low likelihood of off-target amplification in environmental samples.

To assess sequence conservation within the *B. pseudomallei* population, we screened 3,341 high-quality *B. pseudomallei* genome assemblies^44–48^ representing the species’ global diversity (Fig. 1d). Exact matches to the target regions were found in 99.55% of genomes for *TTS1*, 85.33% for *BPSS1836*, and 88.89% for the CRISPR-BP34 target. Given that PCR^42,43^, RPA^49^, and CRISPR-Cas12a^50^ assays can tolerate a small number of mismatches—particularly outside critical regions such as the 3’ end of primers or the seed region of crRNAs—we applied a conservative threshold allowing up to two nucleotide differences. Under this relaxed criterion, the predicted coverage increased to 99.88% for *TTS1*, 94.52% for *BPSS1386*, and 99.76% for CRISPR-BP34 target regions. These results confirm that three targets are both conserved in *B. pseudomallei* and are unlikely to cross-react with non-target organisms, thereby validating their use in molecular assays for environmental surveillance.

### Analytical sensitivity of molecular assays in mixed-species DNA background

**Fig. 2:**
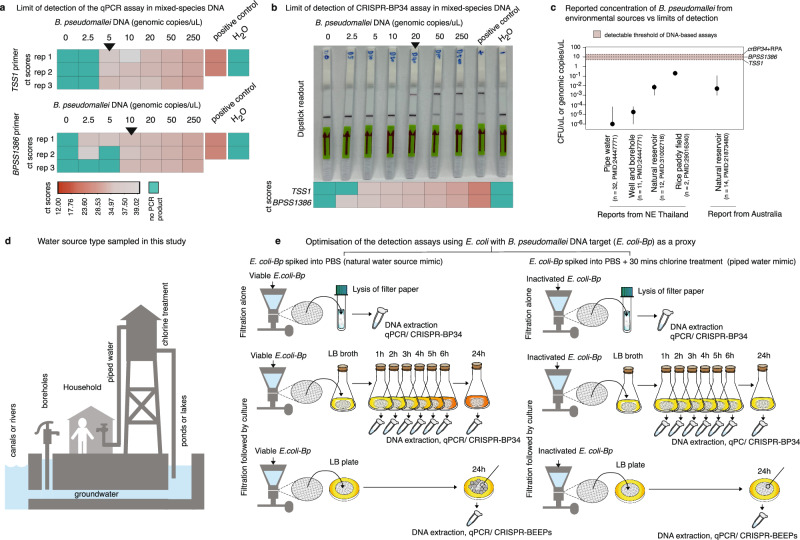
Analytical sensitivity of qPCR and CRISPR-BP34 assays relative to environmental bacterial loads. **a**, **b** Analytical sensitivity in mixed species DNA of qPCR assays using *TSS1* and *BPSS1386* (**a**), and the CRISPR-BP34 system with lateral flow readout (**b**). Each assay was performed in three technical replicates. Ct values are colour-coded: green = no amplification, red gradient = increasing signal intensity (dark red = low Ct, pale red = high Ct). Black triangles indicate the lowest concentration at which three replicates were consistently detected. **c** Comparison of assay detection limits (genomic copies/µL) with reported concentrations of *B. pseudomallei* in environmental water samples (CFU/µL), assuming a 1:1 genome-to-CFU ratio. **d** Schematic illustrating the different water types sampled in this study. **e** Laboratory optimisation workflows for environmental surveillance using *E. coli* carrying the crBP34 DNA target (*E. coli-Bp*) spiked into phosphate-buffered saline (PBS) as a proxy for *B. pseudomallei*, in compliance with biosafety requirements. Workflows included (i) filtration of spiked PBS alone; (ii) filtration of spiked PBS followed by culturing the filter paper in broth; and (iii) filtration of spiked PBS followed by culturing the filter paper on agar plates. DNA was then extracted and analysed by qPCR and CRISPR-BP34 assays. Source data are provided as a Source Data file.

We determined the analytical sensitivity of the CRISPR-BP34 assay and compared it with standard qPCR using a simulated environmental DNA background. Genomic DNA from *B. pseudomallei* was serially diluted into a constant background of DNA extracted from bacteria commonly present in tropical freshwater and piped-water systems^51^. This background comprised DNA from *E. coli*, *Klebsiella pneumoniae*, *Enterobacter cloacae*, *Serratia marcescens*, *Citrobacter freundii*, and *Leclercia adecarboxylata*, representing potential competing environmental DNA. Using these conditions, qPCR detected *B. pseudomallei* at 5 genomic copies/μL using *TTS1* primers and 10 copies/µL using *BPSS1386* primers (Fig. 2a). The CRISPR-BP34 assay, using a lateral-flow dipstick readout, achieved a detection limit of 20 copies/µL (Fig. 2b). By comparison, reported concentrations of viable *B. pseudomallei* in melioidosis-endemic water sources are substantially low (Fig. 2c). These include ~10^−6^ CFU/µL (range: <0.1–6.3 × 10^−^^5^ CFU/µL) in public piped water^52^, ~10^−5^ CFU/µL (range: <0.1–6.5 × 10^−^^5^ CFU/µL) in household wells and boreholes^52^, ~10^−3^ CFU/µL (range: 0.1–1.4 × 10^−^^1^ CFU/µL) in natural water reservoir in northeast Thailand^53^ and Australia^54^. Higher concentrations have been reported in rice paddy fields at ~10^−1^ CFU/µL (range: 5.0 × 10^−^^3^ to 7.5 × 10^−^^1^ CFU/µL) in northeast Thailand^55^ and Vietnam^56^. These data indicate that most environmental water samples contain *B. pseudomallei* at concentrations below the detection limits of direct molecular assays, highlighting the need for a pre-enrichment step to enhance sensitivity for environmental surveillance.

### Optimising assays to distinguish viable *B. pseudomallei*

As the assay is intended for field use, where water is typically collected from both natural sources and chlorinated piped-water systems (Fig. 2d), it is essential to distinguish viable bacteria from residual DNA. Chlorination effectively inactivates bacteria but does not immediately degrade their DNA, which can remain detectable by molecular assays^57,58^. This can produce false-positive molecular signals and unnecessary public-health alerts. To address this, we evaluated three enrichment strategies under two conditions: untreated water, representing natural sources; and chlorine-treated water, representing piped-water sanitation. To comply with biosafety requirements while enabling controlled testing at environmentally relevant concentrations, we used *E. coli* engineered to carry the CRISPR-BP34 target (hereafter *E. coli-Bp*) as a surrogate for *B. pseudomallei*. The analytical sensitivity of molecular detection of *E. coli-Bp* was comparable to that of *B. pseudomallei* (5 genomic copies/μL for both PCR and CRISPR-BP34; Supplementary Fig. 2), supporting its use for assay optimisation. *E. coli-Bp* was spiked at a total concentration of 0, 4, 40, 400, 4000, 40,000, and 100,000 CFU, spanning reported environmental levels of *B. pseudomallei* (10^−6^ to 10^−1^ CFU/µL)^52–55^ when sampling 0.5–5 L of water. Samples were processed using three enrichment approaches: (i) filtration alone; (ii) filtration followed by culturing the filter paper in broth; and (iii) filtration followed by culturing the filter paper on agar plates (Fig. 2e).

**Fig. 3:**
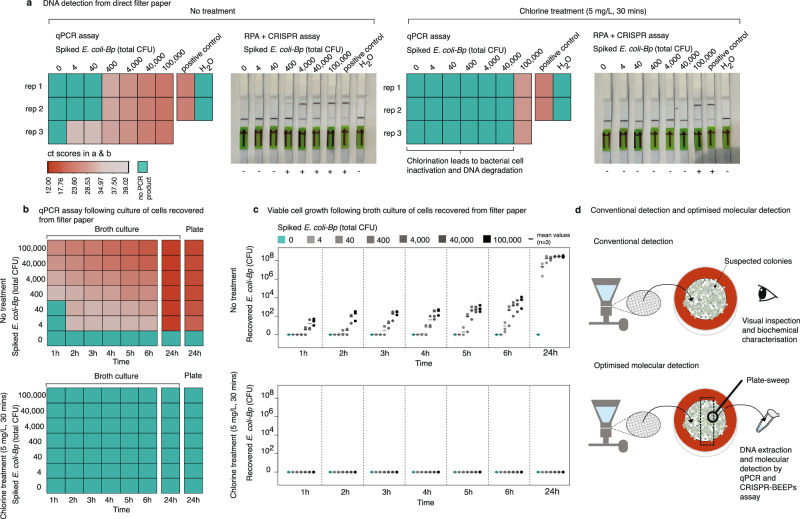
Cell viability-aware molecular detection under chlorine-treated and untreated conditions using *E. coli* carrying crBP34 DNA target as a surrogate. **a** qPCR and CRISPR-BP34 detection from direct filtration of *E. coli-Bp* spiked across a range of 0 to 100,000 CFU, reflecting the expected *B. pseudomallei* loads recovered from filtering 0.5–5 L of environmental water based on previous reports. Filtration was performed under untreated conditions and following chlorine treatment (5 mg/L for 30 min). Each assay was performed in three technical replicates. Ct values are colour-coded (green, no amplification; red gradient, increasing signal intensity). **b** qPCR detection following filtration and subsequent culture of filter papers in broth or on agar plates under untreated and chlorine-treated conditions. For broth cultures, 1 mL aliquots were sampled at 1, 2, 3, 4, 5, 6 and 24 h to assess change in DNA signal over time. For the agar plate, a plate sweep was collected at 24 h using a 10 μL sterile loop for DNA extraction. Each assay was performed in three technical replicates, with means of Ct values plotted. Ct values are colour-coded as in (**a**). **c** Colony-forming unit (CFU) counts as a measure of viable bacteria recovered from filter paper after broth culture under untreated and chlorine-treated conditions. Spiked *E. coli-Bp* concentrations are colour-coded (green, 0 CFU; lighter grey to black, 4–100,000 CFU). At each time point after filter paper inoculation, 100 µL of broth was plated for CFU enumeration. Data represent three independent biological replicates (*n* = 3), and lines indicate the mean across replicates. **d** Schematic of the proposed viability-aware molecular detection workflow based on filtration followed by culture on an agar plate and colony sweeping for DNA extraction, compared with the conventional approach requiring visual inspection of suspected bacterial colonies. For clarity, intermediate sample preservation steps (i.e. temporary storage of plate-sweep material as glycerol stocks prior to DNA extraction) are not depicted. Source data are provided as a Source Data file.

Chlorine treatment at 5 mg/L for 30 min successfully inactivated *E. coli-Bp* across the tested range (0–100,000 CFU), with no visible growth after 24 h of incubation in either broth or on agar (Fig. 3). However, when samples were processed by filtration alone, direct molecular testing still produced positive qPCR and CRISPR-BP34 signals at ≥400 total CFU in untreated samples and at 100,000 CFU in chlorine-treated samples (Fig. 3a). This demonstrates that filtration-based detection cannot distinguish viable from non-viable cells following chlorination. In contrast, filtration followed by culture (broth or agar plate) provided clear discrimination between viable and non-viable bacteria. Chlorinated samples remained qPCR- and CRISPR-BP34-negative after 24 h, whereas untreated samples became positive by qPCR (Fig. 3b) and CRISPR-BP34 (Supplementary Fig. 3), with signal intensity increasing over time in parallel with rising CFU counts (Fig. 3c) and consistent with bacterial growth. Given the comparable performance of broth and agar plate culture and the practical advantages of reduced space requirements and a lower risk of spillage when handling liquid volumes contaminated with *B. pseudomallei*, we selected agar plate culture followed by sweeping the bacterial lawn for DNA extraction as the optimal enrichment strategy. We termed this workflow CRISPR-BEEPs (CRISPR-BP34 with Enhanced Environmental Plate-sweep sampling). This approach (Fig. 3d) ensures that downstream molecular detection reflects viable *B. pseudomallei* rather than residual DNA, improving reliability for surveillance of both natural and chlorinated water sources.

### Field sensitivity and specificity of CRISPR-BEEPs for *B. pseudomallei* detection in environmental water samples from northeast Thailand

**Fig. 4:**
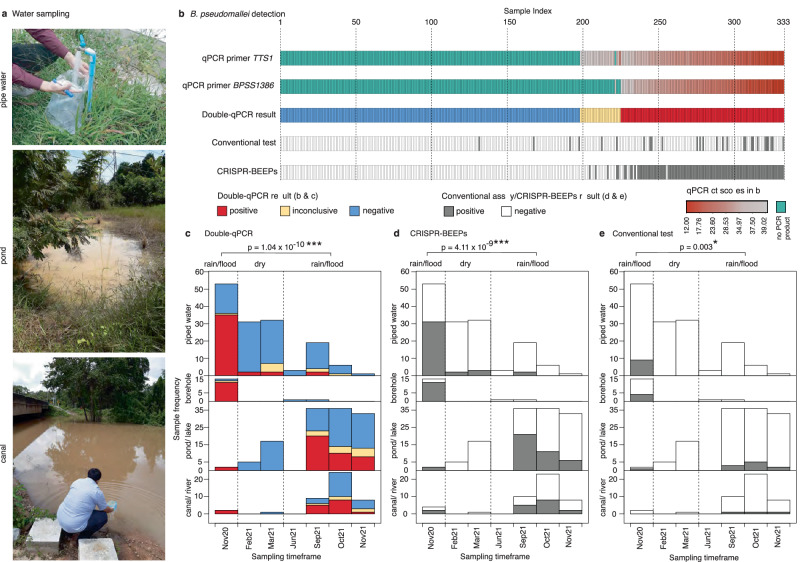
Field performance of conventional detection and CRISPR-BEEPs, using double-qPCR as the reference standard. **a** Representative sampling of locations: household piped water (top), stagnant pond water (middle), and flowing canal water (bottom). **b** Comparison of detection outcomes across assays, including qPCR targeting *TSS1* and *BPSS1386*, combined double-qPCR classification, conventional detection, and CRISPR-BEEPs (*n* = 333 samples). Ct values are colour-coded (green, no amplification; red gradient, increasing signal intensity with dark red indicating low Ct and pale red indicating high Ct). For double-qPCR result: red = positive, yellow = inconclusive, blue = negative. For CRISPR-BEEPs and conventional detection: grey = positive, white = negative. **c**–**e** Seasonal variation in *B. pseudomallei* detection across water sources: piped water (*n* = 145), boreholes (*n* = 15), ponds or lakes (*n* = 129), and canals or rivers (*n* = 44) based on double-qPCR (**c**), CRISPR-BEEPs (**d**), and conventional assay (**e**). Statistical significance of the difference in detection between the rain/flood and dry seasons was assessed using the chi-square test. All tests were two-sided. Source data are provided as a Source Data file.

To evaluate the real-world performance of CRISPR-BEEPs in environmental surveillance, we applied the assay to water samples collected across northeast Thailand, a region endemic for melioidosis. Between November 2020 and November 2021, 356 water samples were collected from household piped water, private boreholes, and natural surface water^47^ (Fig. 4a and Supplementary Fig. 1). All samples were filtered and cultured on selective Ashdown’s agar for three days to select for *B. pseudomallei*. Twenty-one samples were excluded due to fungal overgrowth, leaving 335 interpretable cultures. Using conventional screening based on colony morphology and monoclonal antibody testing^59,60^ (Supplementary Fig. 4), *B. pseudomallei* was identified in only 23 of 87 visually suspicious colonies. This reflects known limitations of culture-based methods: *B. pseudomallei* often grows slowly and may be outcompeted by faster-growing environmental organisms, while colony morphology alone lacks sufficient specificity for accurate identification.

We next applied a molecular plate-sweep approach, harvesting all colony growth from Ashdown plates for DNA extraction, irrespective of colony appearance (Fig. 3d, Supplementary Methods). High-quality DNA was obtained from 333 of the 335 cultures and screened using two qPCR targets (*TTS1* and *BPSS1386*), and the CRISPR-BEEPs assay. Given its higher analytical sensitivity (Fig. 2a, b) and widespread validation, qPCR was used as the reference standard. Samples were classified as *B. pseudomallei*-positive if both qPCR targets were detected (“double-qPCR positive”, *n* = 108), inconclusive if only one target was detected (*n* = 27), and negative if neither target was detected (*n* = 198) (Fig. 4b).

CRISPR-BEEPs showed high concordance with this classification, correctly identifying 101 of 108 double-qPCR positive samples (sensitivity 93.52%, 95% CI: 87.10–97.35%) (Table 1). In contrast, the conventional test detected only 21 of these samples (sensitivity 19.44%, 95% CI: 12.46–28.17%). CRISPR-BEEPs achieved 100% specificity, correctly identifying all qPCR-negative samples (95% CI: 98.15–100.00%), compared with 97.98% specificity for the conventional test (95% CI: 94.91–99.45%). McNemar’s test confirmed the significantly superior performance of CRISPR-BEEPs over the conventional test (*p* = 5.83 × 10^−^^8^). Together, these results demonstrate that CRISPR-BEEPs, which integrates selective enrichment and plate-sweep DNA extraction, offers a highly sensitive, specific, and scalable approach for detecting *B. pseudomallei* in complex environmental water samples.

**Table 1:** Sensitivity and specificity of CRISPR-BEEPs compared to the conventional approach using double-qPCR as the reference standard

	Sensitivity (per cent, 95% CIs)	Specificity (per cent, 95% CIs)
Conventional plate inspection	21 of 108 (19.44%, 12.46–28.17)	194 of 198 97.98% (94.91–99.45)
CRISPR-BEEPs	101 of 108 (93.52%, 87.10–97.35)	198 of 198 (100%, 98.15–100.00)

The diagnostic performance of CRISPR-BEEPs and conventional plate inspection was evaluated using double-qPCR as the reference standard. Sensitivity and specificity are presented as percentages with corresponding 95% confidence intervals (CIs). Sensitivity was calculated based on 108 double-qPCR positive samples, and specificity based on 198 double-qPCR negative samples.

### Enhanced environmental resolution reveals the true burden of *B. pseudomallei* in northeast Thailand, including the piped water system

To characterise the environmental distribution of *B. pseudomallei*, we analysed water type-specific and seasonal detection patterns across northeast Thailand (Fig. 4c–e and Supplementary Table 2). *B. pseudomallei* was detected in all major water types sampled, including public piped water (41/145 samples, 28.3% by double-qPCR; 38/145, 26.2% by CRISPR-BEEPs; compared with 9/145, 6.2% by conventional test), groundwater from private boreholes (11/15, 73.3% by both double-qPCR and CRISPR-BEEPs; 4/15, 26.7% by conventional test), and surface water from stagnant (ponds and lakes) and flowing (canals and rivers) sources (56/173, 32.4% by double-qPCR; 57/173, 32.9% by CRISPR-BEEPs; 14/173, 8.1% by conventional test). Groundwater showed the highest positivity rate in all screening methods, consistent with prior studies^61,62^, and supporting its potential role as a stable environmental reservoir. Subsurface aquifers maintain relatively constant moisture, are shielded from sunlight, and experience limited drying – conditions conducive to long-term bacterial survival. Detection of *B. pseudomallei* in both private boreholes and public piped water is of particular concern. Participant interviews confirmed that these sources are routinely used for household activities such as bathing, dishwashing and food preparation, highlighting a direct route of exposure in endemic areas.

Seasonality had a clear impact on detection rates. During the dry season, many surface water sources such as ponds, lakes, canals and rivers either dried up or receded, reducing sample availability and likely lowering the detectability. In contrast, rainy and flood seasons create conditions that promote bacterial mobilisation. Heavy rainfall can transport *B. pseudomallei* from deeper soil layers to the surface or increase surface runoff into water bodies^63^, thereby enhancing the likelihood of human exposure. Consistent with this, *B. pseudomallei* prevalence in non-groundwater natural water sources was significantly higher during the rainy or flood season. Surface water positivity increased to 56/150 (37.3%) by double-qPCR, 57/150 (38.0%) by CRISPR-BEEPs, and 14/150 (9.3%) by conventional test, compared with 0/23 (0%) by all methods in the dry season (*χ*^2^ *p*_qPCR_ = 6.24 × 10^−5^; *p*_CRISPR_ = 7.46 × 10^−^^4^; *p*_convention_ = 0.264). A similar seasonal pattern was observed for public piped water, with detection increased from 4/63 (6.3%) by double-qPCR, 5/63 (7.9%) by CRISPR-BEEPs, and 0/63 (0%) by conventional test in the dry season to 37/82 (45.1%), 33/82 (40.2%), and 9/82 (11.0%), respectively, during the rainy or flood season (*χ*^2^ *p*_qPCR_ = 1.84 × 10^−^^6^; *p*_CRISPR_ = 2.73 × 10^−^^5^; *p*_convention_ = 0.018). Across a one-year sampling period, detection frequencies obtained from CRISPR-BEEPs closely mirrored those measured by double-qPCR and were consistent with seasonal patterns reported in previous multi-year studies^61,62^. The higher prevalence detected by CRISPR-BEEPs compared with the conventional method suggests that previous reliance on the conventional approach alone may have underestimated the environmental burden of *B. pseudomallei*.

### Environmental exposure and risk of melioidosis

**Fig. 5:**
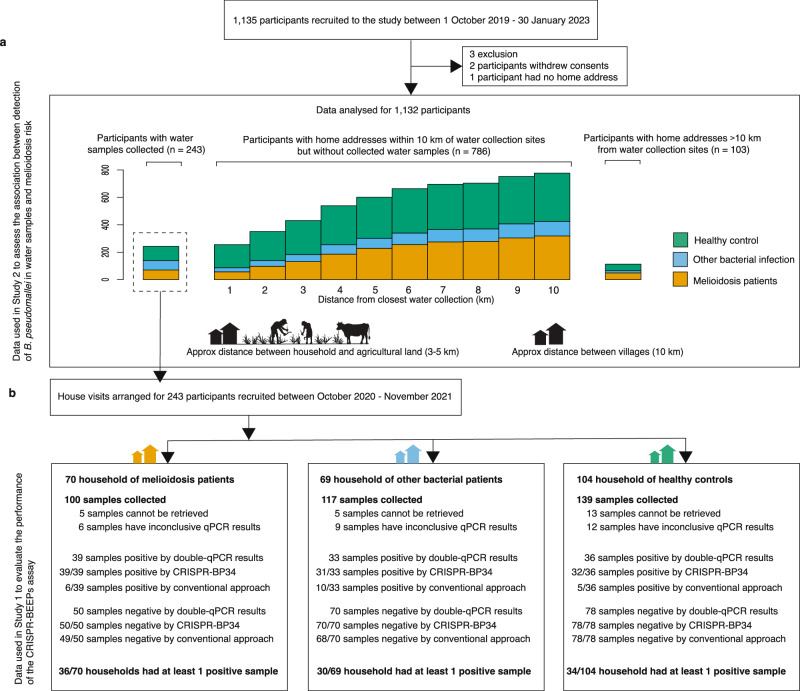
Cohort structure, participant distribution and detection of *B. pseudomallei* in household water samples by assay type. **a** Cohort overview. Stacked bar plots summarise participants enroled across the time period: those with direct water sampling between November 2020 and November 2021, and additional participants from 2019 to 2020 and 2021to 2023 who lived within 1–10 km of the sampling sites but without direct water collection. Participants are grouped by clinical status: clinically-confirmed melioidosis (orange), other bacterial infections (blue), and healthy controls with no history of melioidosis (green). The 3–5 km radius corresponds to the typical travel distance for villagers' agricultural work, while 10 km approximates the distance between neighbouring villages. **b** Detection of *B. pseudomallei* in household water samples from the cohort by assay type. For households with multiple water samples, a household was considered positive if at least one sample tested positive. Source data are provided as a Source Data file.

The association between environmental *B. pseudomallei* and the incidence of melioidosis has largely been described qualitatively, with limited population-level quantitative evidence, in part due to the low sensitivity of conventional environmental detection methods^64^. Using an improved water surveillance protocol, we assessed the presence of *B. pseudomallei* in household and nearby water sources as an indicator of potential environmental exposure and examined whether individuals who either used these water sources directly or lived in proximity to the sampling sites developed melioidosis. Participants were classified into three groups: confirmed melioidosis cases, patients with other bacterial infections, and healthy controls with no prior history of melioidosis. All participants resided within the same endemic regions, allowing for comparison of environmental exposure under similar ecological conditions (Fig. 5). A total of 243 participants enroled between 2020 and 2021 underwent household-level water sampling within three months of melioidosis diagnosis or enrolment, providing a temporally relevant snapshot of environmental exposure (Fig. 5b and Supplementary Fig. 5).

Detection frequencies obtained using CRISPR-BEEPs closely matched those from qPCR, while a conventional method detected fewer positive samples (Supplementary Table 3). *B. pseudomallei* was detected more frequently in households of melioidosis patients than in households of healthy controls. At least one positive water sample was identified in 34/70 (48.6%) melioidosis patient households by both double-qPCR and CRISPR-BEEPs, compared with 34/104 (32.7%) and 33/104 (31.7%) healthy control households by double-qPCR and CRISPR-BEEPs, respectively (two-sided Fisher’s exact test *p*_qPCR_ = 0.04, *p*_CRISPR_ = 0.03). In contrast, detection frequencies in households of melioidosis patients did not differ significantly from those in households of patients with other bacterial infections, where *B. pseudomallei* was detected in 30/69 (43.5%) households by both double-qPCR and CRISPR-BEEPs (*p*_qPCR & CRISPR_ = 0.61). The conventional method showed no significant differences between any household groups. These findings indicate that environmental detection of *B. pseudomallei* is associated with melioidosis when compared with households of healthy participants, but does not distinguish melioidosis from households where participants had other bacterial infections. Univariable regression analyses indicated that melioidosis patients had a higher prevalence of established risk factors such as diabetes mellitus and agricultural occupation when compared to patients with other bacterial infections (Supplementary Tables 4 and 5). These findings suggest that environmental exposure alone is insufficient to cause disease and that host-related factors are crucial in determining progression from exposure to clinical infection.

### Combined effect of environmental and host factors

**Fig. 6:**
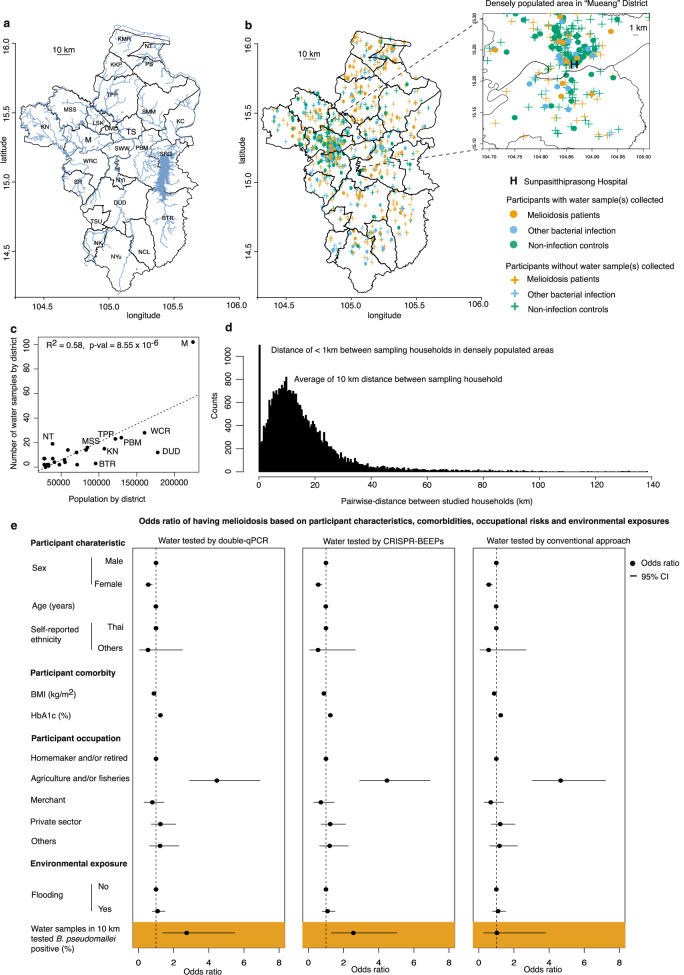
Association between B*. pseudomallei* detection in water sources and melioidosis incidence. **a** Map of Ubon Ratchathani province in northeast Thailand, where 1012 of 1135 study participants reside, highlighting local water systems and terrain. **b** Locations of participant households, including those with directly collected water samples and those without samples but residing in close proximity. **c** Positive correlation between the number of water samples collected and district-level population density, estimated using linear regression. **d** average distance (in kilometres) between participant households in the cohort. **e** Adjusted odds ratios from multivariable logistic regression showing associations between environmental detection of *B. pseudomallei* in water sources and melioidosis risk. Dots indicate odds ratios, and error bars represent 95% confident intervals. Detection of *B. pseudomallei* in water samples located within 10 km of participant households was used as a spatial proxy for nearby environmental presence. The analysis includes melioidosis cases (*n* = 395) and non-melioidosis controls (*n* = 634), comprising individuals with other bacterial infections and healthy individuals living in the same endemic region who never developed melioidosis. Statistical significance varies by detection method. Detection approaches include double-qPCR (two DNA targets, each measured in two technical replicates per sample), CRISPR-BEEPs (one DNA target with two technical replicates per sample), and the conventional culture-based plate inspection approach (two technical plates per sample). Positivity criteria are described in the “Methods” section. All tests are two-sided. Districts are abbreviated: Buntharik (BTR), Don Mot Daeng (DMD), Det Udom (DUD), Khong Chiam (KC), Kut Khaopun (KKP), Khemarat (KMR), Khuang Nai (KN), Lao Suea Kok (LSK), Mueang (M), Muang Sam Sip (MSS), Na Chaluai (NCL), Nam Khun (NK), Na Tan (NT), Na Yia (NYi), Nam Yuen (NYu), Phibun Mangsahan (PBM), Pho Sai (PS), Samrong (SR), Sawang Wirawong (SWW), Sirindhorn (SRD), Si Mueang Mai (SMM), Trakan Phuet Phon (TPP), Tan Sum (TS), Thung Si Udom (TSU), Warin Chamrap (WRC). To protect participant privacy in accordance with the Thailand Personal Data Protection Act, individual household geolocation data are not publicly available. All spatial analyses and figures are based on anonymised, aggregated data that cannot be used to identify individual participants or households. Source data are provided as a Source Data file.

To further investigate the combined roles of host susceptibility and environmental exposure, we analysed data from a larger cohort of 1132 participants enroled between 2019 and 2023 (Figs. 5a and 6a, b). Although household-level water sampling was not conducted for this group, environmental samples were collected across all districts, with sampling density proportional to population distribution (Fig. 6c, R^2^ = 0.58, p = 8.55 × 10^−6^). The average distance between sampling locations was approximately 10 km, corresponding to a typical distance between villages in the study region (Fig. 6d). In more densely populated areas, such as the central “Mueang” district, sampling was more frequent to ensure adequate environmental representation relative to the population density. Multivariable logistic regression analysis was conducted using covariates that were significant in univariate models (Supplementary Table 5). These also included *B. pseudomallei* detection by double-qPCR, CRISPR-BEEPs, and conventional assays, assessed at multiple spatial scales ranging from the household level up to 10 km from a participant’s residence (Fig. 6e and Supplementary Tables 6–15). Results from double-qPCR and CRISPR-BEEPs were consistent. Both methods identified environmental detection of *B. pseudomallei*, along with host comorbidities and occupation, as independent predictors of melioidosis.

Host susceptibility was a determinant of disease risk. Poor glycaemic control, reflected by elevated HbA1c levels, was strongly associated with increased risk of melioidosis (adjusted OR _qPCR_ = 1.25 [95% CI: 1.19–1.31], *p*_qPCR_ < 2 × 10^−16^; adjusted OR _CRISPR_ = 1.25 [1.19–1.31], *p*_CRISPR_ < 2 × 10^−16^). Individuals working in high-exposure occupations, including agriculture, fisheries, or farming, also had higher odds of infection (adjusted OR _qPCR_ = 4.46 [2.91–6.91], *p*_qPCR_ = 1.20 × 10^−11^; adjusted OR _CRISPR_ = 4.47 [2.92–6.93], *p*_CRISPR_ = 7.23 × 10^−9^). In contrast, female sex (adjusted OR _qPCR_ = 0.56 [0.40–0.78], *p*_qPCR_ = 5.74 × 10^−4^; adjusted OR _CRISPR_ = 0.56 [0.41–0.78], *p*_CRISPR_ = 1.14 × 10^−3^), and being overweight or obese (adjusted OR _qPCR_ = 0.88 [0.84–0.91], *p*_qPCR_ = 1.06 × 10^−11^; adjusted OR _CRISPR_ = 0.88 [0.84–0.91], *p*_CRISPR_ = 1.05 × 10^−12^) were associated with lower risk. These protective associations likely reflect reduced participation in high-exposure agricultural work, particularly compared to lean male agricultural labourers who experience frequent environmental contact with *B. pseudomallei*.

Environmental detection of *B. pseudomallei*, when measured using sensitive molecular methods, also independently predicted melioidosis incidence. Using double-qPCR detection, *B. pseudomallei* positivity within a 7 km radius of the participant’s household was significantly associated with disease risk (adjusted OR _qPCR_ 1.91 [1.09–3.34], *p*_qPCR_ = 0.024, Supplementary Table 9). CRISPR-BEEPs showed a similar association at a 9 km radius (adjusted OR _CRISPR_ 1.87 [1.01–3.5], *p*_CRISPR_ = 0.048, Supplementary Table 7). The association strengthened further at a 10 km radius – a distance corresponding approximately to the administrative area of a typical rural village (Fig. 6e). At this scale, qPCR detection of *B. pseudomallei* was associated with an adjusted OR _qPCR_ of 2.74 (1.38–5.48, *p*_qPCR_ = 0.004), while CRISPR-BEEPs detection yielded an adjusted OR _CRISPR_ of 2.56 (1.31–5.04, *p*_CRISPR_ = 0.006). In contrast, conventional culture-based plate methods failed to detect any significant relationship between environmental positivity and melioidosis incidence. These findings highlight the importance of sensitive molecular diagnostics for identifying high-risk environmental exposure and support village-level risk mapping as a strategy for targeted intervention.

## Discussion

Our study introduces CRISPR-BEEPs as a sensitive and resource-efficient approach for environmental surveillance of *B. pseudomallei*, compatible with decentralised laboratory settings. It outperformed conventional plate inspection and matched the analytical performance of standard qPCR while being more tolerant of environmental inhibitors and requiring minimal instrumentation (see cost and other considerations for ideal point-of-care test based on WHO REASSURED guidelines, Supplementary Tables 16 and  17). Crucially, the use of sensitive molecular detection enabled a pioneering detection of a statistically supported association between *B. pseudomallei* in household and community water sources and clinical melioidosis. This relationship, long hypothesised but previously unsupported by robust data, was not detectable using conventional culture-based plate inspection alone^64^ which is prone to low sensitivity. In contrast, culture enrichment followed by plate-sweep molecular detection using double-qPCR or CRISPR-BEEPs revealed strong and reproducible associations between positive water exposure and disease risk.

The applicability of CRISPR-BEEPs is currently restricted to water samples. Although soil is an important environmental reservoir for *B. pseudomallei*, its physicochemical heterogeneity and high abundance of PCR- and CRISPR-inhibitory compounds present substantial technical barriers for reliable detection. Adapting similar CRISPR-based approaches for soil surveillance will likely require dedicated soil-specific extraction and detection workflows. Addressing these challenges represents an important direction for future methodological development.

Our assay optimisation relied on *E. coli* carrying *B. pseudomallei* target sequence as a surrogate organism due to biosafety requirements. While analytical sensitivity was comparable between the surrogate and the target organism (Fig. 2a, b and Supplementary Fig. 2), biological differences between *E. coli* and *B. pseudomallei*—including growth kinetics, selective culture requirements, and resistance to chlorination—resulted in different culture windows for optimisation (*E. coli*, 24 h) and field deployment (*B. pseudomallei*, 72 h). Notably, *B. pseudomallei* exhibits markedly greater tolerance to chlorine than *E. coli*: standard disinfection conditions (5 mg/L free chlorine with 30-min contact^65^) effectively inactivate most *E. coli* strains, whereas viable *B. pseudomallei* has been recovered from water containing up to 1000 mg/L free chlorine^16^. This disparity has implications for both water treatment and environmental surveillance in melioidosis-endemic regions, indicating that standard chlorination may reduce bacterial load without completely eliminating viable *B. pseudomallei* from piped water systems.

The continued requirement for a culture step remains a constraint, introducing time delays and laboratory dependence. However, this step is currently indispensable for discriminating viable from non-viable organisms. For environmental pathogens such as *B. pseudomallei*, DNA-only detection may reflect past contamination or prior disinfection (e.g. chlorination) rather than ongoing exposure risk, as demonstrated in Fig. 3a. Culture enrichment selectively amplifies signals from viable cells, thereby strengthening the epidemiological relevance of downstream molecular detection. At the same time, locus-specific DNA assays are subject to inherent limitations, including potential off-target amplification due to horizontal gene transfer and false negatives arising from sequence divergence among strains. To address these issues, target selection was guided by in silico genomic analyses, and detection was benchmarked against a double-qPCR framework requiring concordant amplification of two independent genomic targets, improving species specificity and confidence in positive calls.

We also acknowledged that environmental sampling coverage was incomplete: with household water samples obtained from 243 of 1132 participants (356 samples total), primarily due to SARS-CoV-2-related travel restrictions and logistical constraints. Despite this limitation, repeated detection of *B. pseudomallei* in water samples from 2020 to 2021 suggests the presence of persistent environmental reservoirs, potentially in deep soil or groundwater, that may continue to contaminate surface and piped water sources. This interpretation aligns with the spatial and temporal distribution of clinical melioidosis cases observed between 2019 and 2020, and from 2021 to 2023, although causal inference should be made cautiously.

Collectively, these findings identify water exposure to both natural and piped water as a potential contributor to melioidosis risk. The detection of *B. pseudomallei* in piped water highlights critical deficiencies in water and sanitation, including inadequate chlorine dosing, ageing infrastructure, and system failures that sustain environmental contamination. Although our study demonstrates an ecological association rather than direct transmission from a specific source, it provides an evidence base for urgent intervention. Findings have been communicated to the Thailand Provincial Waterworks Authority and the Regional Ministry of Public Health to support immediate policy review. In line with SDG 6—ensuring access to clean water and sanitation for all—our results demonstrate that improving water treatment, distributing infrastructure, and routine monitoring represent a tractable and high-impact strategy for preventing severe bacterial disease in vulnerable communities and lowering disease burden.

## Methods

### Study design

We conducted two interrelated studies to improve *B. pseudomallei* detection sensitivity in environmental samples and to assess the association between environmental presence and melioidosis clinical incidence.

Study 1 evaluated the performance of CRISPR-BEEPs against a conventional approach, with a double-qPCR assay as the reference standard. A total of 356 water samples were collected between November 2020 and November 2021 from households and natural water sources used by 243 participants, who were part of the case-control cohort from Study 2 in Ubon Ratchathani and nearby provinces in northeast Thailand, areas known to be endemic for melioidosis. The sampling framework captured environmental heterogeneity, including variation in water source types and hydrological conditions such as flood-prone and dry areas. The diagnostic component of this study adhered to the Standards for Reporting of Diagnostic Accuracy (STARD) guideline.

Study 2 investigated the association between the presence of *B. pseudomallei* in water samples and the incidence of melioidosis in households of cases and controls. From October 2019 to January 2023, a total of 1135 participants were recruited from a melioidosis cohort. This cohort consisted of 439 melioidosis patients, 190 patients with other community-acquired infections, and 506 healthy controls. Melioidosis and other infectious cases were recruited immediately after a culture-confirmed diagnosis at Sunpasitthiprasong Hospital, using hospital computer records. Controls were selected from blood donors or diabetic outpatients at the same hospital. After obtaining informed consent, demographic information such as age, sex, ethnicity and underlying health conditions was extracted from medical records, while participants provided details about their exposure risks and household locations (Supplementary Methods). Data were recorded in MACRO EDC (version 4), a clinical management system, and de-identified prior to downstream analysis. Participants received 100 THB as compensation for their time participating in the research. This amount is approximately equivalent to the cost of a meal in Thailand.

Participants whose water samples were collected in Study 1 included 70 melioidosis patients, 69 patients with other bacterial infections, and 104 healthy controls from Study 2. Sampling locations were geographically distributed across the study region to capture spatial variation in exposure at the scale relevant to individual risk and broadly reflected district-level population density. To address sample variability, multiple water samples were collected from the household and surrounding areas of 80 participants, including 74 cross-seasonal samples to account for seasonal fluctuations (Supplementary Fig. 3). Both the human participant study and environmental sample collection were approved by the Sunpasitthiprasong Hospital Ethical Review Board (015/62C) and the Oxford Tropical Research Ethics Committee (OxTREC 25–19). Written informed consent was obtained from all participants. The full study protocol is described in ref. ^47^ and followed the Strengthening the Reporting of Observational Studies in Epidemiology (STROBE) guideline.

### In silico evaluation of qPCR (*TSS1*, *BPSS1386*) and CRISPR-BEEPs (RPA + *crBP34*) target specificity and coverage

To evaluate the specificity of the selected DNA targets, we performed in silico screening using BLASTn^66^ (v.2.16.0) against 27,771 MAGs derived from ecological samples^39^ collected across tropical and subtropical regions. To assess conservation across the global *B. pseudomallei* population, the same amplicons were also screened against 3341 high-quality *B. pseudomallei* genome assemblies^44–48^ using BLAT (v. 36). Bulk download of MAG dataset is available from the Joint Genome Institute GEMs database (https://genome.jgi.doe.gov/GEMs), and the accession codes for *B. pseudomallei* genome assemblies used in this study are available at 10.6084/m9.figshare.31742224.

The screened sequences include the full amplicons generated by the forward and reverse primers of the *TSS1* (115 bp), *BPSS1386* (185 bp), and CRISPR-BEEPs, which encompass the RPA primer sites and *crBP34* region (118 bp) (Supplementary Table 1 and Supplementary Fig. 5). All partial hits were examined for mismatches and their locations, as the position of mismatches influences assay performance. Based on published tolerance thresholds, we considered up to three mismatches outside the 3’ end for qPCR primers^42,43^, up to three non-clustered mismatches and outside 3’ end for RPA primers^49^, and one to two mismatches in non-seed regions for the CRISPR-Cas12a crRNA^50^ as acceptable.

### qPCR and CRISPR-BP34 assay conditions

DNA samples were screened using multiple primer sets, including the *TTS1* and *BPSS1386* qPCR assays, which were used for cross-validating results in environmental detection, and the CRISPR-BP34 primer set, which was the assay evaluated in this study. All oligonucleotides used in this study, including qPCR primers, RPA primers, and CRISPR crRNA, are listed in Supplementary Table 1, with full assay details provided in the Supplementary Methods. Primers and crRNA were synthesised commercially and purified by standard desalting (Macrogen, Korea). All assay components described in this study are available from the corresponding author upon request.

For qPCR assays, primers’ efficiency was evaluated prior to the analysis (Supplementary Fig. 6). All reactions were performed in duplicate in a 20-μL reaction volume. The qPCR protocol consisted of an initial denaturation step at 95 °C for 10 min, followed by 40 cycles of denaturation at 95 °C for 15 s, with annealing temperatures at 61 °C for *TTS1*, 64 °C for *BPSS1386*, and 62 °C for the CRISPR-BP34 primer set.

For the CRISPR-BP34 assay, DNA extracts were first amplified by RPA using TwistAmp® Basic (Cat#TABAS03KIT, TwistDx, Maidenhead, UK), with CRISPR-BP34 primers added at 0.48 nM, and 2-6 μL of genomic DNA included in each 30-μL reaction. RPA reactions were incubated at 39 °C for 30 min, and the resulting amplicons were transferred directly into the CRISPR-cas12 reaction (described in the Supplementary Methods), and incubated at 37 °C for 60 min. Detection was performed using a HybriDetect universal lateral flow strip (Cat#MGHD1, Milenia Biotec, Giessen, Germany), which was dipped into the reaction for 5 min.

### Determination of molecular sensitivity for qPCR and CRISPR-BP34 in mixed background DNA

To determine the analytical sensitivity of both methods, we performed a spiking experiment in which a defined concentration of *B. pseudomallei* DNA (0, 2.5, 5, 10, 20, 50, and 250 copies/μL) was added into a 2 μL volume of pan-microbial DNA background (Fig. 2c). The background consisted of DNA from bacterial species commonly found in water sources, including *E. coli, K. pneumoniae, E. cloacae, Serratia odorifera, C. freundii*, and *L. adecarboxylata*^51^. The pan-microbial DNA was prepared at a final concentration of 278 ng/µL to match concentrations observed in real samples. This setup recreated the complexity of natural microbial communities and enabled us to determine the minimum detectable concentration of *B. pseudomallei* for both qPCR and CRISPR-BP34.

### Optimisation of molecular assays with and without culture

As the limits of detection for both qPCR and CRISPR-BP34 (5 - 20 copies/μL) are higher than the reported concentrations of *B. pseudomallei* (10^−6^ to 10^−1^ CFU/μL), an enrichment step was required to either concentrate or proliferate the bacterial cells to detectable levels. To meet biosafety requirements during protocol optimisation, *E. coli* carrying the RPA and crBP34 target sequence (*E. coli-Bp*) was used as a surrogate. *E. coli-Bp* was cultured in LB broth to an OD_600_ = 0.400, DNA was extracted with the QIAamp kit, quantified by Qubit, and diluted to 0, 2.5, 5, 10, 20, 50, and 250 copies/μL. Both qPCR and CRISPR-BEEPs assays showed comparable limits of detection for *E.coli-Bp* DNA and *B. pseudomallei* DNA (Supplementary Fig. 2), confirming *E. coli-Bp* is a suitable proxy for assay optimisation.

To mimic CFU levels observed in field samples, *E. coli-Bp* was spiked into 40 mL PBS at final concentrations of 0, 4, 40, 400, 4000, 40,000, 100,000 and 400,000 CFU. These concentrations represent an estimated *B. pseudomallei* range of 0.5 to 500,000 CFU in typical filtration volumes of 0.5 to 5 L of water^52^, based on reported concentrations of 10^−6^ to 10^−1^ CFU/μL (Fig. 2c). Three enrichment approaches were evaluated: filtration alone, filtration followed by broth enrichment, and filtration followed by plate enrichment. Each approach was tested under two conditions: untreated water and water treated with 5 mg/L calcium hypochlorite (70% available chlorine) for 30 min to mimic household chlorination practices in the study communities. Chlorination did not fully inactivate *E. coli-Bp* at a total load of 400,000 CFU. Therefore, this concentration was excluded to avoid ambiguity between viable cells and residual DNA.

### Protocol optimisation using filtration alone

In the filtration-alone method, each spiked sample was passed through a 0.45-μm cellulose paper filter (40.5 mm diameter, Sartorius, Germany) using vacuum suction. DNA was extracted directly from the filter using the QIAGEN DNeasy PowerWater Kit (Cat#14900-100-NF, USA). The entire filter was transferred into a 5-mL bead-beating tube, where lysis was achieved by vortexing in a QIAGEN buffer. Following protein and inhibitor removal, total DNA was captured on an MB Spin Column, washed, and eluted for analysis using qPCR and CRISPR-BP34. Results were compared between chlorine-treated and untreated samples.

### Protocol optimisation using filtration with broth culture

For the filtration-with-broth-culture method, each spiked sample was filtered as described above, and the filter paper was transferred into 40 mL of LB broth. Cultures were incubated at 37 °C with shaking. At each time point (1–6 h and 24 h), 1 mL aliquots were collected for DNA extraction using the QIAamp DNA Micro Kit (cat #56304, USA), followed by qPCR, and CRISPR-BP34 analysis. Viable cell growth was assessed by CFU enumeration from duplicate 100 μL platings for each time point. Optical density (OD_600_) was measured from 1 mL aliquots as an additional indicator of bacterial growth. Results were compared between chlorine-treated and untreated samples.

### Protocol optimisation using filtration with plate culture

For the filtration-with-plate-culture method, each spiked sample was filtered as described above, and the filter was then placed directly onto an LB agar plate and incubated at 37 °C for 24 h. Plates were monitored for colony development during incubation. After 24 h, all bacterial growth on the filter was collected by sweeping the filter surface with a sterile 1 μL-loop and resuspended in 1 mL PBS for DNA extraction using QIAamp DNA Micro Kit (cat #56304, USA), and the recovered material was analysed by qPCR and CRISPR-BP34. Results were compared between chlorine-treated and untreated samples.

### Collection and processing of environmental samples

The filtration-with-plate-culture method was applied to environmental samples for *B. pseudomallei* detection. A total of 356 samples were collected from sites within a 120 km driving distance from the MORU laboratory at Sunpasitthiprasong Hospital to enable same-day processing. Five-litre water samples were collected from each household, except during the peak of SARS-CoV-2 outbreaks, when nearby communal water reservoirs were sampled due to visitation restrictions. Participants’ water sources included piped water, boreholes, ponds, lakes, canals or rivers (Fig. 3c and Supplementary Fig. 1). Samples were transported in sterile plastic bags to the laboratory within 3 h and processed promptly. To minimise overgrowth by competing microorganisms, different filtration volumes were evaluated. A filtration volume of 0.5 L was selected as optimal, as larger volumes resulted in excessive background growth that hindered visual inspection of colonies resembling *B. pseudomallei*. Each sample was filtered, and the filter membrane was placed onto Ashdown^67^ agar to selectively support *B. pseudomallei* growth while inhibiting other microbes. Plates were incubated at 40 °C, and examined daily. Plates overgrown with fungi were excluded from further analysis. After three days of incubation, the microbial lawn, including both visible and non-visible *B. pseudomallei*, was swept from the filter surface on the plate using a 10 μL sterile loop and preserved in 1 mL glycerol stock (20% *v*/*v*, VWR, Belgium) at −80 °C for subsequent culture and re-sweep for DNA extraction. All procedures were conducted in an enhanced biosafety level 2 laboratory, but with biosafety level 3 practices. Personnel performing the tests were blinded to the results of the other methods.

### Detection of *B. pseudomallei* in environmental samples using a conventional method

For conventional screening, plates cultured on Ashdown agar were examined daily for colonies showing characteristic *B. pseudomallei* morphology, including dry, wrinkled, metallic, or purple colony appearances. Colonies with these typical features were then subjected to confirmatory testing using monoclonal antibody-based assays^59,60^ (Supplementary Fig. 4). These antibody assays specifically detect the exopolysaccharide of *B. pseudomallei* and have been used to distinguish true *B. pseudomallei* from morphologically similar environmental bacteria.

### Detection of *B. pseudomallei* in environmental samples using optimised molecular assays

For molecular testing, microbes were revived from plate-sweep glycerol stocks for each sample. An aliquot of 20 μL from each glycerol stock was plated onto Ashdown agar, followed by re-sweeping of the resulting growth for bacterial DNA extraction using QIAGEN Genomic-tips [Cat#10243, Germany] to preserve high molecular-weight pan-microbial DNA and minimise DNA shearing (Supplementary Methods). The extracted DNA had an average concentration of 278.42 ng/μL (IQR 181.00 – 433.23) and an A260/A280 purity score ranging from 1.68 to 1.94, ensuring high yield and low impurities, suitable for both qPCR detection and CRISPR-BEEPs.

Double-qPCR detection was performed using two independent primer sets—*TTS1* and *BPSS1386*, to cross-validate results. Assay conditions were identical to those described earlier in the assay development section. Samples were processed in batches alongside known positive and negative controls. The cycle threshold (Ct) values were recorded for each sample. The individuals interpreting the results were blinded to the source of the water samples. A sample was classified as positive if qPCR amplification occurred for both primers and the melting temperatures were consistent with the expected values. Samples were classified as inconclusive if amplification with correct melting temperature was observed with only one primer. Samples were considered negative if no amplification was detected or if amplification occurred with incorrect melting temperatures.

CRISPR-BEEPs detection followed the protocol described in the assay development section. DNA samples were first amplified using RPA, and the resulting amplicons were added to the CRISPR reaction. Detection was performed using the HybriDetect universal lateral flow assay kit. Lateral flow strip kits were read visually by three independent individuals, each blinded to the results of other assays. Two bands indicated a positive result, while a single band signified a negative result.

### Statistical analysis of environmental *B. pseudomallei* detection and melioidosis risk

Environmental prevalence of *B. pseudomallei* was estimated for each detection method (double-qPCR, CRISPR-BEEPs, and conventional assay) by calculating the proportion of positive water samples relative to the total number collected within the radii ranging from 1 km to 10 km of each participant's household (Supplementary Tables 6 and 15). Distances were computed based on each participant’s geoposition using the R package “geosphere”^68^ (v. 1.6-5). Associations between *B. pseudomallei* detection in household and neighbourhood water sources, participants’ occupations, health conditions, and disease status (binary outcomes) were examined using univariable analysis to explore individual associations (Supplementary Table 5). Multivariable logistic regression models^69^ were then fitted to estimate adjusted associations while accounting for all covariates (Fig. 6e and Supplementary Tables 6 and 15). Independence of observations was ensured by study design; linearity of continuous predictors on the log-odds scale and absence of substantial multicollinearity were evaluated prior to modelling. All tests were two-sided with a significance level of 0.05. Analyses and visualisation were performed in R (version 4.5.2). Individual-level data underlying this analysis are not publicly available due to participant consent restrictions related to sharing individual-level data and to protect participant privacy.

Sensitivity analyses were conducted to evaluate the robustness of the results under various conditions. First, we restricted the study population to the actual water sampling period from November 2020 to November 2021. Second, we expanded the study period to include data from October 2019 to January 2023. Third, we assessed the geographical ranges of exposure by using different radius distances from each household. Additionally, we analysed the data by season to account for observed bacterial persistence in the environment across different seasons and the potential latency period, which may span several years before melioidosis develops^7^.

### Power calculation and sample size determination

For study 1, which assessed the performance of the CRISPR-BEEPs assay, the minimum required sample size was calculated using the formula *n* = *z*^2^*p*(1 − *p*)/*d*^2^, with *z* as the 95% confidence interval at 1.96; *p* representing the prevalence at 0.5; and *d* as the margin of error at 0.1. At least 96 positive and 96 negative environmental samples were needed to validate the conventional approach and CRISPR-BEEPs. Sensitivity and specificity were computed using double-qPCR results as the reference, with the 95% confidence interval estimated based on a binomial assumption. McNemar’s test was used to compare detection performance with paired data.

For study 2, which assessed the association between melioidosis status and detection of *B. pseudomallei* in household or nearby water sources using assays evaluated in study 1, sample size calculations were based on previously reported exposure prevalence of 7% in cases and 3% in controls, obtained using conventional detection methods^64^ where an estimated sensitivity is approximately 20%. This observed prevalence corresponds to the estimated true exposure prevalence of 35% (7%/0.20) in cases and 15% (3%/0.20) in controls. Under this assumption, a sample size of 70 cases and 70 controls provided 80% power to detect a difference at a two-sided significance level of α = 0.05. The number of samples collected directly from participant households exceeded this minimum requirement, with 243 households sampled and 356 water samples collected.

### Reporting summary

Further information on research design is available in the Nature Portfolio Reporting Summary linked to this article.

## Supplementary information

Supplementary Information

Reporting Summary

Transparent Peer Review file

## Source data

Source Data

## Data Availability

De-identified participant data generated in this study are available under restricted access due to ethical and consent constraints, limiting use to purposes consistent with the approved study protocol^47^. Requests should be submitted to the corresponding author (claire@tropmedres.ac) with a brief proposal outlining the intended use. Requests are reviewed for compliance with ethical approvals and consent conditions, with responses provided within 2–4 weeks. Approved access is subject to a data use agreement. Processed data generated in this study are provided in the Supplementary Information and Source Data. Environmental metagenomic and *B. pseudomallei* sequence data used in this study are available in the repositories referenced in refs. ^39,44–46,48^. Bulk downloads of the metagenome-assembled genome dataset are available from the Joint Genome Institute GEMs database (https://genome.jgi.doe.gov/GEMs). Accession codes for *B. pseudomallei* genome assemblies used in this study are available via Figshare 10.6084/m9.figshare.31742224. Source data are provided with this paper.
