# Supplementary material for: CRISPR-based environmental detection of Burkholderia pseudomallei identifies sanitation gaps and melioidosis risk in northeast Thailand
Source: Nat Commun. 2026 May 15;17:6460. doi: 10.1038/s41467-026-73286-8 (PMC13376406; doi:10.1038/s41467-026-73286-8)
Supplement: Supplementary file 1 — Supplementary Information [file 41467_2026_73286_MOESM1_ESM.pdf]

Supplementary Information

CRISPR-based environmental detection of *Burkholderia pseudomallei* identifies sanitation gaps and melioidosis risk in northeast Thailand

Sukritpong Pakdeerat*, Chalita Chomkatekaew*, Phumrapee Boonklang*, Raiwin Mothong*, Maturada Patchsung, Arin Wongprommoon, Kesorn Angchagun, Yaowaret Dokket, Areeya Faosap, Gumphol Wongsuwan, Premjit Amornchai, Vanaporn Wuthiekanun, Jiramate Changklom, Suwatthiya Siriboon, Parinya Chamnan, Sharon J Peacock, Julian Parkhill, Jukka Corander, Nicholas PJ Day, Nicholas R Thomson, Chayasith Uttamapinant, Somsakul Pop Wongpalee & Claire Chewapreecha†

*These authors contribute equally
† Corresponding author: claire@tropmedres.ac

This Supplementary Information includes Supplementary Methods, Supplementary Figures 1-6, Supplementary Table 1 – 17, and Supplementary References

Table of Contents

Supplementary methods **4**

Study 1 Protocol: Environmental screening for B. pseudomallei **4**

Field water collection and plate culture 4
Conventional plate inspection method 5
Plate-sweep and DNA extraction method 5
CRISPR-BP34 6

Study 2 Protocol: a case-control observational cohort study **7**

Study design, sites and population 7
Participant inclusion criteria 7
Ethics/protection of human subjects 8
Other prevalent bacterial infections identified in other infectious controls 9
Examples of cases and controls with multiple water samples collected 10

Supplementary Figures **11**

Supplementary Figure 1 Type of water samples collected 11
Supplementary Figure 2 Engineering of *E. coli* carrying CRISPR-BP34 target and validation as a proxy for *B. pseudomallei*. 13
Supplementary Figure 3 CRISPR-BP34 signal intensity increases with cell proliferation over time .. 15
Supplementary Figure 4 Screening approaches 17
Supplementary Figure 5 Participants with multiple water samples collected. 19
Supplementary Figure 6 Efficiency, coverage, cycle threshold (ct) scores of PCR primers used in this study. 21

Supplementary Tables **23**

Supplementary Table 1: A list of primers and oligos used in this study 23
Supplementary Table 2: Seasonal fluctuations in *B. pseudomallei* positivity detected by molecular and conventional methods 24
Supplementary Table 3: Household-level detection of *B. pseudomallei* by molecular and conventional methods and its association with melioidosis in residents 25
Supplementary Table 4: Demographic characteristics of the studied population 26
Supplementary Table 5: Univariate logistic regression of factors associated with melioidosis 28
Supplementary Table 6: Multivariable logistic regression of factors associated with melioidosis based on *B. pseudomallei* detection within 10 km 29
Supplementary Table 7: Multivariable logistic regression of factors associated with melioidosis based on *B. pseudomallei* detection within 9 km 30
Supplementary Table 8 Multivariable logistic regression of factors associated with melioidosis based on *B. pseudomallei* detection within 8 km 31
Supplementary Table 9 Multivariable logistic regression of factors associated with melioidosis based on *B. pseudomallei* detection within 7 km 32
Supplementary Table 10 Multivariable logistic regression of factors associated with melioidosis based on *B. pseudomallei* detection within 6 km 33
Supplementary Table 11 Multivariable logistic regression of factors associated with melioidosis based on *B. pseudomallei* detection within 5 km 34
Supplementary Table 12 Multivariable logistic regression of factors associated with melioidosis based on *B. pseudomallei* detection within 4 km 35

Supplementary Table 13 Multivariable logistic regression of factors associated with melioidosis based on *B. pseudomallei* detection within 3 km........ 36
Supplementary Table 14 Multivariable logistic regression of factors associated with melioidosis based on *B. pseudomallei* detection within 2 km........ 37
Supplementary Table 15 Multivariable logistic regression of factors associated with melioidosis based on *B. pseudomallei* detection within 1 km........ 38
Supplementary Table 16 Stages of CRISPR-BEEPs development benchmarked by WHO REASSURED guideline........ 39
Supplementary Table 17 Established PCR test benchmarked against WHO REASSURED criteria . 40

Supplementary References 41

SUPPLEMENTARY METHODS

STUDY 1 PROTOCOL: ENVIRONMENTAL SCREENING FOR *B. PSEUDOMALLEI*

This study aims to compare and improve the environmental screening protocols for *Burkholderia pseudomallei* by comparing the conventional approach[1] against the alternative CRISPR-BEEPS method[2,3], using two primer-based qPCR assays as reference tests.

Reviews of existing methods of water sample surveillance for *B. pseudomallei*

The current guideline[1] for environmental sampling of *B. pseudomallei* was established in 2013 by the Detection of Environmental *B. pseudomallei* Working Party (DEBWorP). This guideline focuses on soil and water sampling, primarily using conventional culture-based methods on selective media, followed by visual inspection of colonies that resemble *B. pseudomallei*. These traditional methods have been widely used to study the environmental presence of *B. pseudomallei* (**Supplementary Fig. 1**), helping define its ecological habitats in regions such as Australia, Oceania, Southeast Asia, Africa, and South America [4–12]. For water sampling, the protocol involves collecting 1 to 5,000 mL of water and concentrating the bacteria using filtration, centrifugation, or potassium alum precipitation. Detection follows selective media, such as Ashdown[13] agar containing gentamicin and crystal violet, after which colonies are visually examined for characteristic *B. pseudomallei* morphologies and isolated for subsequent characterisation. However, the rise of antibiotic-resistant microbes complicates the differentiation of *B. pseudomallei* from other environmental bacteria.

To improve detection, recent studies have introduced molecular methods such as PCR-based assays[14–17], mass spectrometry[18,19], and sequencing[19,20], often following culture with Ashdown agar or modified Ashdown broth. qPCR-based methods have shown a significant increase in test positivity rate compared to conventional using Ashdown agar alone. In Laos, qPCR nearly doubled the positivity rate when applied to the same water samples[21]. Similarly, a study in Australia demonstrated that a qPCR method using samples enriched in Ashdown's broth achieved a positivity rate of 69.2%, while the conventional method yielded only 5.5%[22]. Although effective, molecular techniques often require complex and costly equipment, which may not be available in resource-limited settings. To overcome this issue, we have developed CRISPR-BEEPS[2,3], an assay that detects *B. pseudomallei* nucleic acids without complex equipment, making it suitable for resource-limited areas.

Field water collection and plate culture

Following protocol optimisation (**Supplementary Fig. 2-3**), a culture step remained essential to distinguish viable from non-viable cells, particularly for piped water samples, where chlorination may eliminate viable bacteria while leaving detectable DNA for molecular assays. Plate culture was selected over broth enrichment to improve storage efficiency and reduce spillage risk when handling large volumes of water potentially contaminated with *B. pseudomallei*. Five litres of water were collected from either household piped water or a neighborhood water reservoir[23]. To minimise overgrowth by competing microorganisms that can obscure visual detection of *B. pseudomallei*, and to reduce membrane clogging during vacuum filtration, 500 mL of water was

selected as an optimal volume that balance reduced background flora with sufficient volume to capture *B. pseudomallei*. The water was passed through two 0.45-μm filters, with 250 mL filtered through each to prevent clogging of the vacuum. Both filters were then cultured on Ashdown agar to enrich for *B. pseudomallei* for downstream confirmation.

Conventional plate inspection method
The conventional method, including confirmation using monoclonal antibodies (**Figure 3d**, **Supplementary Fig. 4**), was carried out following the procedures outlined in [24].

Plate-sweep and DNA extraction method
The Plate Sweep method begins with recovering *B. pseudomallei* or other Gram-negative bacteria from lawn mixed colonies on Ashdown agar (**Supplementary Fig. 4**). The bacteria colonies are then harvested by sweeping the plate with a 10 μL-loop, after which the bacterial pellet is resuspended in 5 mL of PBS to wash the cells. It is important to ensure all representative strains are included by sweeping from the plate. The mixture is centrifuged at 3000g for 5 minutes, and the supernatant is removed by quickly decanting the liquid into a waste bin. The bacterial pellet is resuspended in 5 mL of PBS, vortexed at top speed to ensure a homogeneous mixture, and centrifuged again at 3000g for 5 minutes before the supernatant is removed. All procedures were conducted in an enhanced biosafety level 2 laboratory but with biosafety level 3 practices.

For DNA extraction, 7 μL of RNase A solution (100 mg/mL) is added to 3.5 mL of Buffer B1, which should be prepared fresh and stored at 2-8°C. Lysozyme is dissolved in distilled water to a concentration of 100 mg/mL and stored at -20°C, while Buffer QF is warmed to 50°C to increase the DNA yield. Isopropanol and 70% v/v ethanol should also be stored at -20°C and placed on ice during the procedure. Bacterial lysis begins by resuspending the bacterial pellet in 3.5 mL of Buffer B1 (with RNase A) and vortexing it thoroughly. Then, 80 μL of lysozyme stock solution (100 mg/mL) and 100 μL of proteinase K are added, followed by incubation at 37°C for at least 30 minutes. Next, 1.2 mL of Buffer B2 is added, mixed by inverting the tube several times, and incubated at 50°C for 1-2 hours until the mixture becomes clear, indicating complete lysis. If lysis is incomplete, extend the incubation or vortex the mixture before separating the bacterial debris by centrifugation at 5000g for 10 minutes at 4°C.

For DNA purification, a Qiagen genomic tip is equilibrated with 4 mL of Buffer QBT when 10 minutes remain in the previous incubation. The lysed sample is then applied to the genomic tip using a Pasteur pipette and allowed to pass through by gravity flow. The genomic tip is washed twice with 7.5 mL of Buffer QC. During DNA elution and precipitation, the genomic DNA is eluted with 5 mL of prewarmed Buffer QF and collected in a new 50 mL centrifuge tube. Depending on the volume, 800 μL of the eluted DNA is aliquoted into labelled 2 mL microcentrifuge tubes. DNA precipitation is achieved by adding 560 μL of ice-cold isopropanol and inverting the tubes 20-30 times until the DNA precipitates as a white, feather-like strand. If the DNA is not visible, proceed with the steps regardless. The DNA is stored at -20°C overnight.

The following day, the tubes are kept on ice, and the precipitated DNA is centrifuged at maximum speed at 4°C for 5 minutes. The supernatant is carefully discarded, and the DNA pellet is washed

with 1 mL of ice-cold 70% v/v ethanol. An alternative method involves transferring the DNA pellet into one tube with 200 µL of ethanol, creating a final volume of 1.2 mL. The DNA is centrifuged again, and the remaining supernatant is removed carefully using a P20 pipette if necessary. The DNA pellet is air-dried for 5-10 minutes, ensuring it does not overdry. If large droplets remain, incubate the pellet on a 55°C heat block for 3-5 minutes. To dissolve the DNA, add 35 µL of nuclease-free water (or ~200 µL for the alternative method) and incubate it overnight or at 55°C for 1-2 hours. Finally, the DNA is stored at 2-8°C for short-term storage or at -20°C for up to 6 months.

CRISPR-BP34

Expression and purification of MBP-LbCas12a

The expression and purification of MBP-LbCas12a were performed according to the procedures detailed in the previous study[2].

CRISPR RNA Synthesis

The CRISPR RNA, crBP34, previously reported[2], was used in this study. The synthesis of crRNA followed the same methods outlined in the prior study.

Recombinase polymerase amplification (RPA)

RPA was performed using the TwistAmp Basic kit (TwistDx, USA, #TABAS03KIT) following the manufacturer's protocol, with the following modifications:
(i) The total reaction volume was adjusted to 30 µL,
(ii) Incubation was carried out at 39°C for 30 minutes,
(iii) DNA input was limited to 2 µL, as adding more DNA did not improve sensitivity and could introduce inhibitors, and
(iv) MgOAc was added last to initiate the reaction. All RPA reactions were stored at -20°C until use.

RPA primers 148 and 149 (**Supplementary Table 1**), previously reported[2], were synthesised and purified by Macrogen (Korea) using standard desalting.

CRISPR reaction and dipstick detection

CRISPR reactions were conducted in a 50-µL volume containing 100 nM crRNA, 200 nM MBP-LbCas12a, 100 nM FAM-biotin probe, 5 µL RPA product, and 1x HOLMES buffer (2 mM spermidine, 40 mM Tris pH 8.5, 6 mM $MgCl_2$, 1 mM DTT, 40 mM glycine, 0.001% v/v Triton X-100, and 0.4% w/v PEG-20,000). The reaction was incubated at 37°C for 60 minutes. After incubation, a lateral flow dipstick (Milenia Biotec, Germany, #MGHD1) was inserted into the reaction mixture and allowed to develop for 5 minutes before reading the results.

qPCR

Two sets of qPCR primers, *TTS1* (primers 192-193) and *BPSS1386* (primers 270-271)(**Supplementary Table 1**), were used to detect *B. pseudomallei* genomic DNA in samples. Since environmental microbes can exchange genetic material via horizontal gene transfer, both primer

sets, targeting different genes, were employed to ensure the accuracy of the detection. The primers were synthesised and desalted by Macrogen (South Korea).

qPCR reactions were prepared in a 20-µL volume using Maxima SYBR Green/ROX qPCR Master Mix (ThermoFisher Scientific, USA, #K0221), following the manufacturer's protocol. The qPCR conditions included: an initial denaturation at 95°C for 10 minutes, followed by 40 cycles of denaturation at 95°C for 15 seconds, with annealing temperatures of 61°C for *TTS1* and 64°C for *BPSS1386.* Each sample was processed in duplicate along with known positive and negative controls. The cycle threshold (Ct) values were recorded for each sample, and positive detection was defined as any duplicate reaction yielding a positive result for either primer set. Standard melting curve analysis was performed at the end of the PCR and compared to the melting profiles of the positive controls. The ΔRn threshold was automatically determined by the built-in software.

STUDY 2 PROTOCOL: A CASE-CONTROL OBSERVATIONAL COHORT STUDY

Study design, sites and population

One of the objectives of this study was to investigate the risk factors associated with melioidosis infection. To achieve this, demographic information, occupation, and environmental exposure data, along with water samples were collected from participants, including melioidosis cases, other infections, and healthy controls, living within a 120 km radius of Sunpasitthiprasong Hospital in Ubon Ratchathani and nearby provinces, where high incidences of melioidosis are observed. The hospital, which hosts a satellite unit of the Mahidol-Oxford Tropical Medicine Research Unit (MORU), also facilitated the collection and processing of water samples for the study. The full study protocol is available in Angchagun *et al.* 2023[23].

Care was taken to maintain the separation of cases and controls throughout the study. The control group had no documented history of melioidosis at the point of the study end date in January 2023. We acknowledged the transfer of controls to cases. Throughout the study period, one control with underlying diabetes has become infected with melioidosis and thus was transferred into the case category. This accounted for 0.2 % of the control population.

Given the similar clinical manifestations between melioidosis and tuberculosis (TB), individuals with active TB were excluded from the study. Additionally, other underlying health conditions that may increase susceptibility to bacterial infections were also excluded. The complete inclusion and exclusion criteria are outlined below.

Participant inclusion criteria

Melioidosis cases:

- Participants must be aged 18 years or older.
- A culture-confirmed infection of *B. pseudomallei* must be obtained from any clinical sample.
- Participants must be willing to participate in the study, with written informed consent provided by the patient or their relative.
- Participants must have resided in northeast Thailand for at least the previous two years.

Other Infection controls:
- Participants must be aged 18 years or older.
- A culture-confirmed infection by bacterial pathogens other than *B. pseudomallei* must be obtained.
- Participants must be willing to participate in the study, with written informed consent provided by the patient or their relative.
- Participants must have resided in northeast Thailand for at least the previous two years.

Healthy controls:
- Participants must be aged 18 years or older.
- Participants should currently be in good health (non-diabetic) or be outpatients from a diabetic clinic, without any other medical problems requiring hospital supervision.
- Participants must be willing to participate in the study, with written informed consent provided by the patient or their relative.
- Participants must have resided in northeast Thailand for at least the previous two years.

Participant exclusion criteria
Melioidosis cases:
- Current tuberculosis (TB) or treatment for TB within the last six months.
- Documented human immunodeficiency virus (HIV) infection, chemotherapy, or other immunosuppressive therapies in the last 12 months.
- Pregnancy.

Other Infection controls:
- Previous history of melioidosis.
- Current tuberculosis (TB) or treatment for TB within the last six months.
- Documented human immunodeficiency virus (HIV) infection, chemotherapy, or other immunosuppressive therapies in the last 12 months.
- Pregnancy.

Healthy controls:
- Previous history of melioidosis.
- Current tuberculosis (TB) or treatment for TB within the last six months.
- Documented human immunodeficiency virus (HIV) infection, chemotherapy, or other immunosuppressive therapies in the last 12 months.
- Pregnancy.

Ethics/protection of human subjects
Screening
All participants meeting the recruitment criteria were approached by a research nurse for eligibility screening. A written participant information sheet (PIS) and informed consent form (ICF) in Thai were provided to participants or their relatives if they lacked capacity. The PIS detailed the study's nature, implications, constraints, known side effects, and risks. Participants were given a

minimum of ten minutes to consider the information and had the opportunity to ask questions before deciding whether to participate.

Informed consent was obtained through a dated signature from the participant or their relative, as well as from the person obtaining consent. For illiterate participants, a thumbprint was used. A signed copy of the ICF was provided to the participant or responsible relative, while the original signed forms were retained at the study site.

Ethics/Protection of Human Subjects

The study received ethical approval from the Sunpasitthiprasong Hospital Ethical Review Board (015/62C) and the Oxford Tropical Research Ethics Committee (OxTREC 25-19). All research data sets were pseudonymised to protect participant identities and allow for feedback or withdrawal of consent. No participant names, identities, or exact GPS locations of their households will be disclosed.

Other prevalent bacterial infections identified in other infectious controls

Among the infection control group (n = 190), the most prevalent infections were *Escherichia coli* (n = 65, 35%), followed by *Klebsiella pneumoniae* (n = 40, 21%), coagulase-negative *Staphylococcus spp.* (n = 11, 5.9%), and *Acinobacter baumanii* (n = 9, 5.3%). One patient was co-infected with both *K. pneumoniae* and *A. baumanii.* These findings align with previous studies on community-acquired infections in adults from northeast Thailand (2013-2017[25] and 2004-2010[26]) and a broader study in rural communities in Southeast Asia[27].

Examples of cases and controls with multiple water samples collected

Melioidosis case

A monk reported using mixed water sources, including piped water and water pumped from nearby ponds. His daily alms round, a mindfulness practice where he walked barefoot through the village to receive food from villagers, further exposed him to potential environmental risks. Five water samples were taken: from the temple's piped water, the pond water used in the temple, a pond at the temple entrance, a canal flowing past the temple and village, and a lake serving as a water reservoir. All but the pond at the temple entrance tested positive for *B. pseudomallei* (4 out of 5 samples, 80%). The monk had been admitted for melioidosis earlier that year.

Healthy control

A homemaker reported using public piped water at home. Five water samples were collected, including household piped water and ponds surrounding her village. Three pond water samples tested positive for *B. pseudomallei* (3 out of 5 samples, 60%). Although her daily routine was home-based, making environmental exposure unclear, she reported not having melioidosis up to the time of enrollment.

Other infection control

A third case is an employer who used public piped water at home. Five water samples were collected, including a sample from household piped water and four samples from nearby ponds. Only one pond sample tested positive for *B. pseudomallei* (1 out of 5 samples, 20%). He was infected with *Escherichia coli* and had no reported history of melioidosis at the time of the study.

Supplementary Figures

Supplementary Figure 1 Type of water samples collected and potential environmental exposure

The study was conducted in northeast Thailand, a melioidosis-endemic region with a tropical monsoon climate characterised by distinct wet and dry seasons and mean annual temperatures ranging from approximately 23-37 C. The area lies within the lower Mekong River basin and consists largely of low-lying floodplains subjected to seasonal flooding. Land use is predominantly agricultural, particularly irrigated rice cultivation, and communities rely on multiple water sources, including piped water, boreholes, and surface water bodies. Water samples collected in this study encompassed these key sources, including public piped water, groundwater from private boreholes, and surface water from rivers, canals, lakes, and ponds. Together, these reflect the primary environmental niches and exposure pathways for *Burkholderia pseudomallei*, shaped by monsoon-driven dynamics, through routine activities such as drinking, bathing, food preparation, and agricultural use.

Supplementary Figure 1

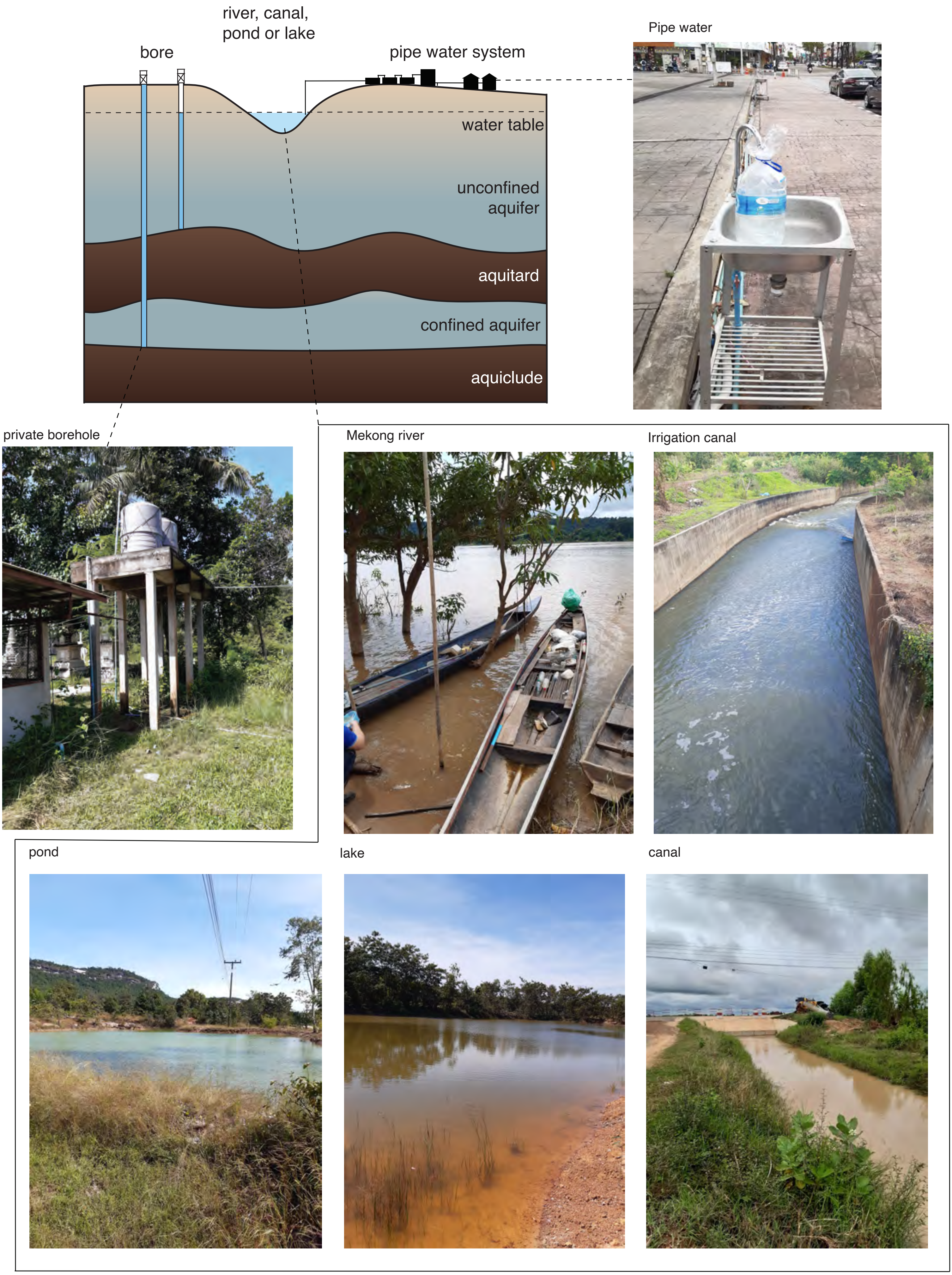

Supplementary Figure 2 Engineering of *E. coli* carrying CRISPR-BP34 target and validation as a proxy for *B. pseudomallei*.

Due to biosafety constraints associated with large-volume optimisation experiments, *Escherichia coli* was engineered to carry the RPA and CRISPR-BP34 target sequence, as described in [3]. (a) Generation of *E. coli-Bp*. The CRISPR-BP34 target fragment was cloned into the *pACYCDuet-1* plasmid at the *NcoI* site. The inserted fragment, comprising the RPA amplicon and crRNA target sequence, was PCR-amplified and integrated into the *lacZ* locus of *E. coli NEB 10-beta* using λ Red recombinase–mediated homologous recombination. Correct genomic insertion was confirmed by PCR and sequencing. (b,c) Analytical sensitivity of qPCR (b) and the CRISPR-BP34 assay with lateral-flow readout (c) using *E. coli-Bp* genomic DNA across concentrations of 0, 2.5, 5, 10, 20, 50, and 250 genomic copies/µL. Ct values are colour-coded (green, no amplification; red gradient, increasing signal intensity). Black triangles indicate the lowest concentration at which all three replicates were consistently detected. Each assay was performed in three biological replicates. qPCR Ct values represent the mean of three replicates, and lateral-flow dipsticks were performed for all replicates. Source Data are provided as Source Data file.

Supplementary Figure 2

a CRISPR-BP34 target fragment cloned into pACYCDuet-1 and integrated into the *lacZ* locus of *Escherichia coli* NEB 10-beta (*E. coli-Bp*) for assay optimisation

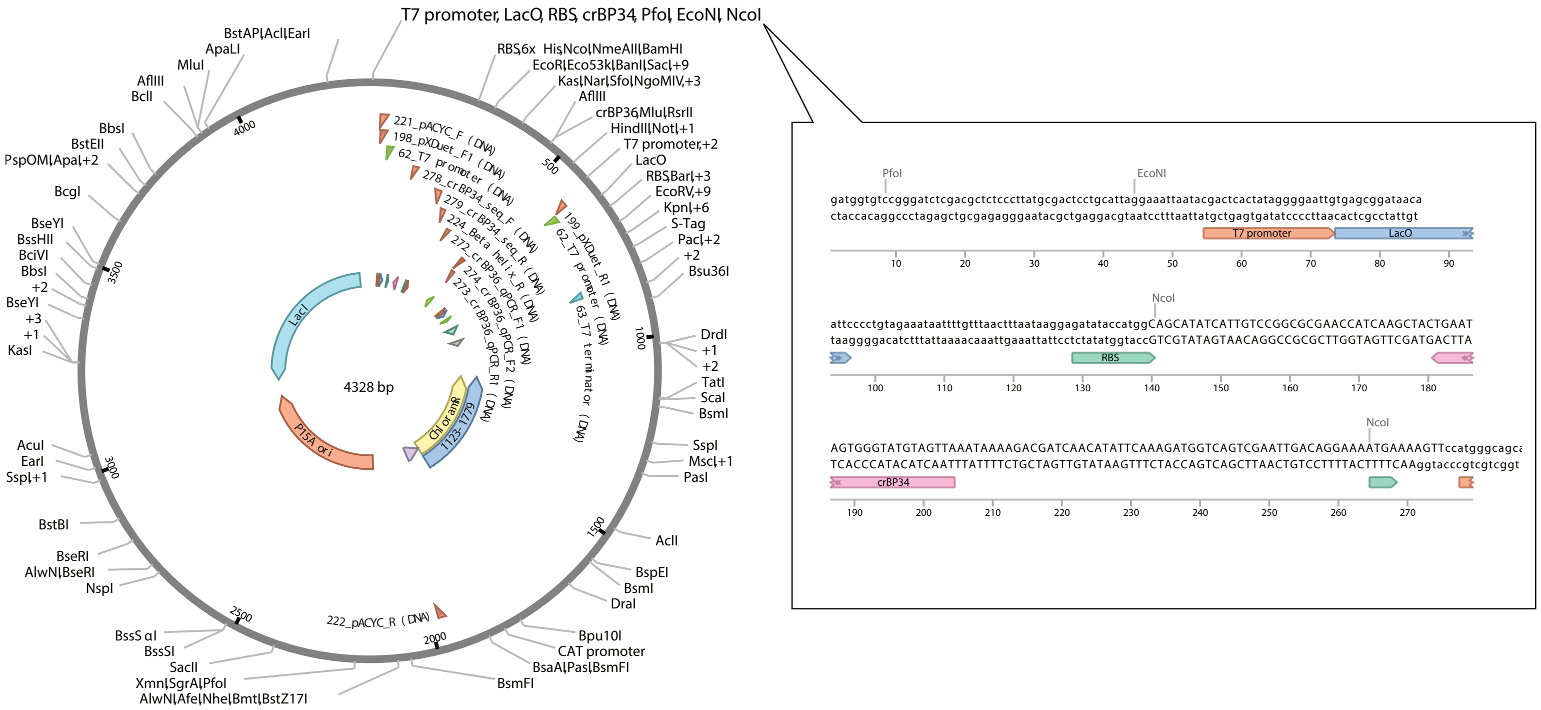

b Limit of detection of qPCR assay using *E. coli-Bp* as a proxy

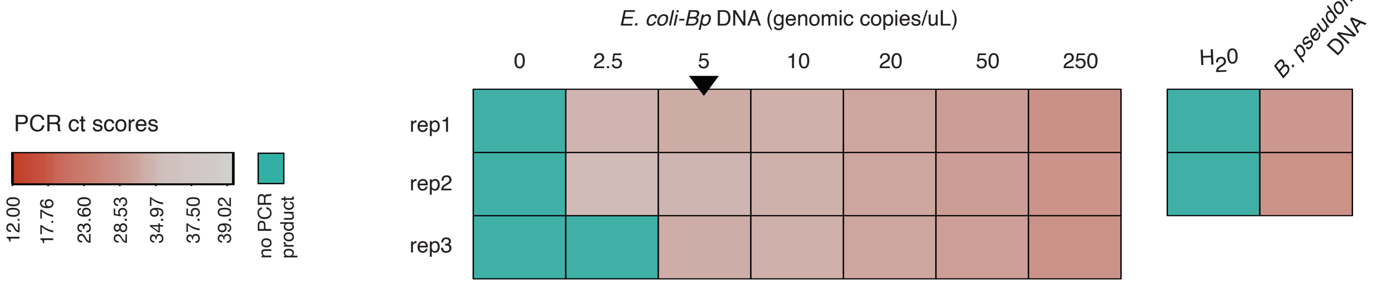

c Limit of detection of RPA+crBP34 assay using *E. coli-Bp* as a proxy

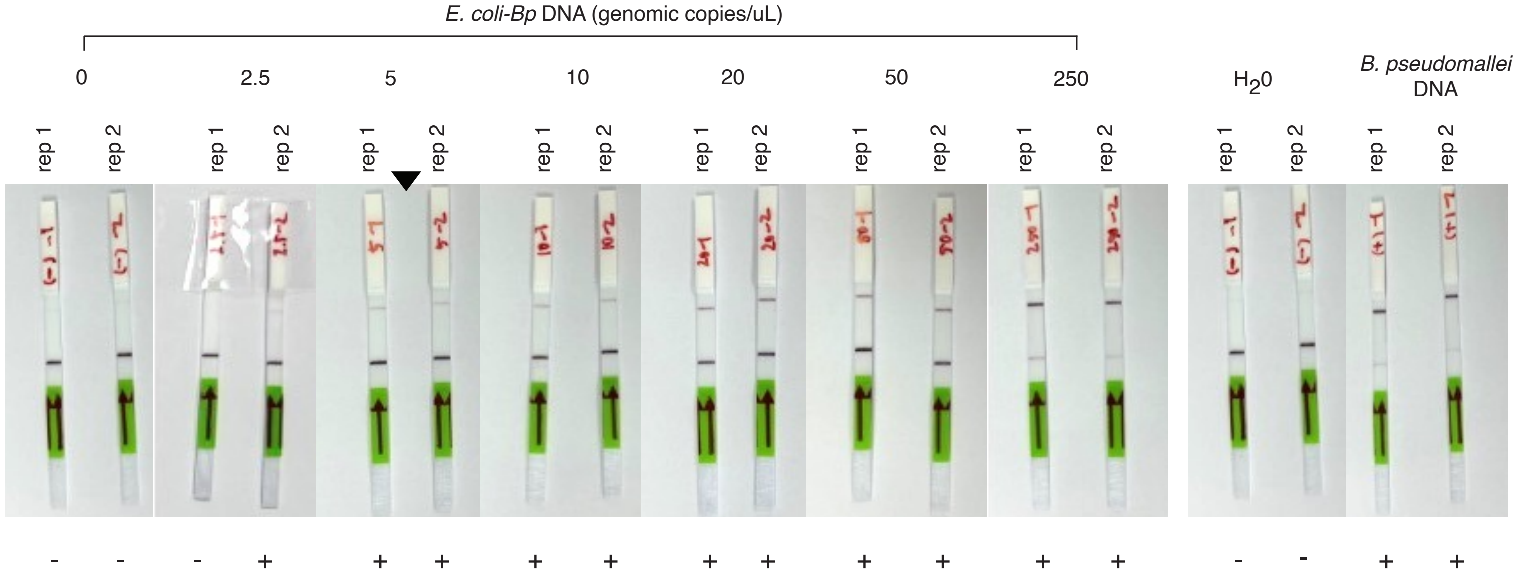

Supplementary Figure 3 CRISPR-BP34 signal intensity increases with cell proliferation over time

Samples were spiked with *E. coli-Bp* across a range of initial concentrations (0, 4, 40, 400, 4,000, 40,000, and 100,000 total CFU) and captured on filter paper. (a). Filter papers were cultured in broth and sampled at 1, 2, 3, 4, 5, 6, and 24 hours for DNA extraction and CRISPR-BP34 analysis. (b) In a parallel setup, filter papers were cultured on agar plates and sampled at 24 hours by CRISPR-BEEP (plate sweep for DNA extraction and CRISPR-BP34 analysis). CRISPR-BP34 dipstick signal intensities obtained from both broth- and plate-based methods correspond to CFU counts shown in Fig 3c. The initial experiment was performed in three replicates (Figure 3b, c); one replicate was randomly selected for lateral-flow dipstick analysis. Source Data are provided as Source Data file.

Supplementary Figure 3

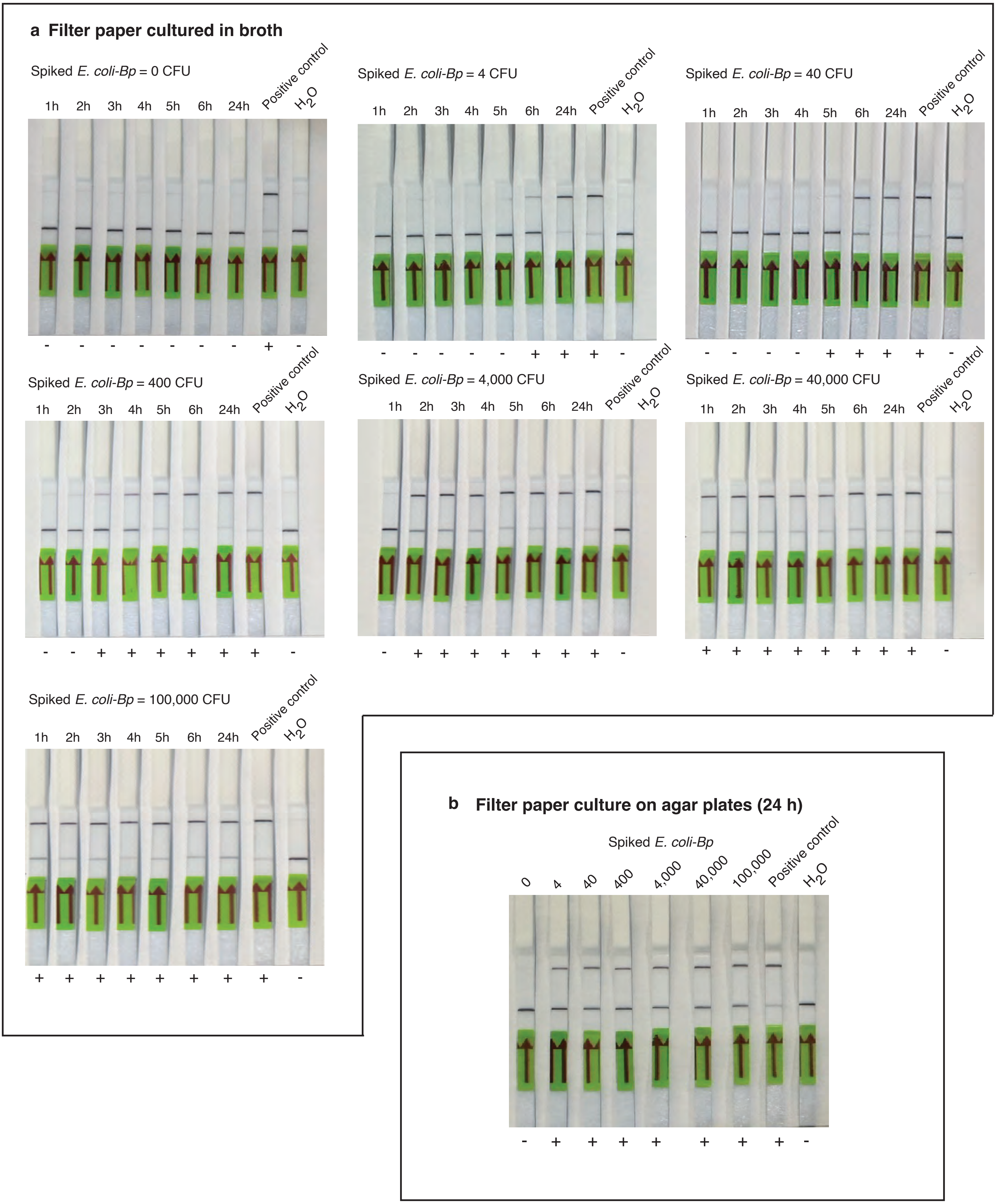

Supplementary Figure 4 Screening approaches

(a) Morphological characteristics of *B. pseudomallei* used for species identification on Ashdown agar plates. (b) Conventional method: A sample of suspected *B. pseudomallei* grown on filtered water paper is placed on an Ashdown agar plate. Suspected colonies (white circles) were re-cultured onto fresh Ashdown agar and confirmed using monoclonal antibody tests. (c) Alternative plate-sweep approach: bacteria grown in modified Ashdown broth[21,22] or on filtered papers placed on Ashdown agar were plate-swept for DNA extraction, followed by testing with CRISPR-BEEPs (the assay used in this study) or PCR (reference assay).

Supplementary Figure 4 Typical morphology of *Burkholderia pseudomallei* and comparison between conventional and alternative plate-sweep screening methods for water samples

a Morphology of *Burkholderia pseudomallei* colonies on solid media like Ashdown's agar

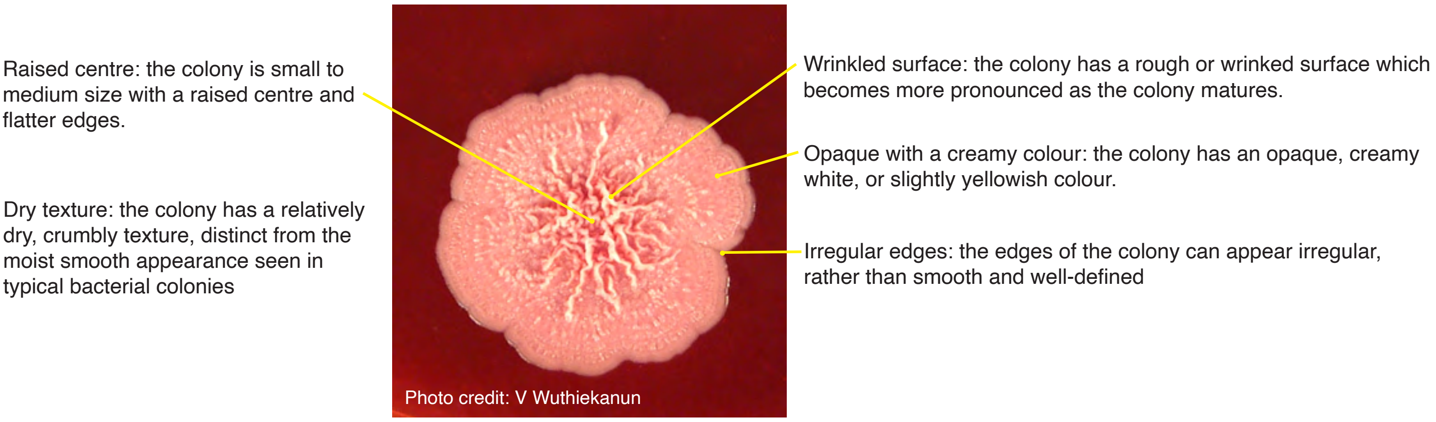

b Conventional plate inspection followed by species confirmation with monoclonal antibodies

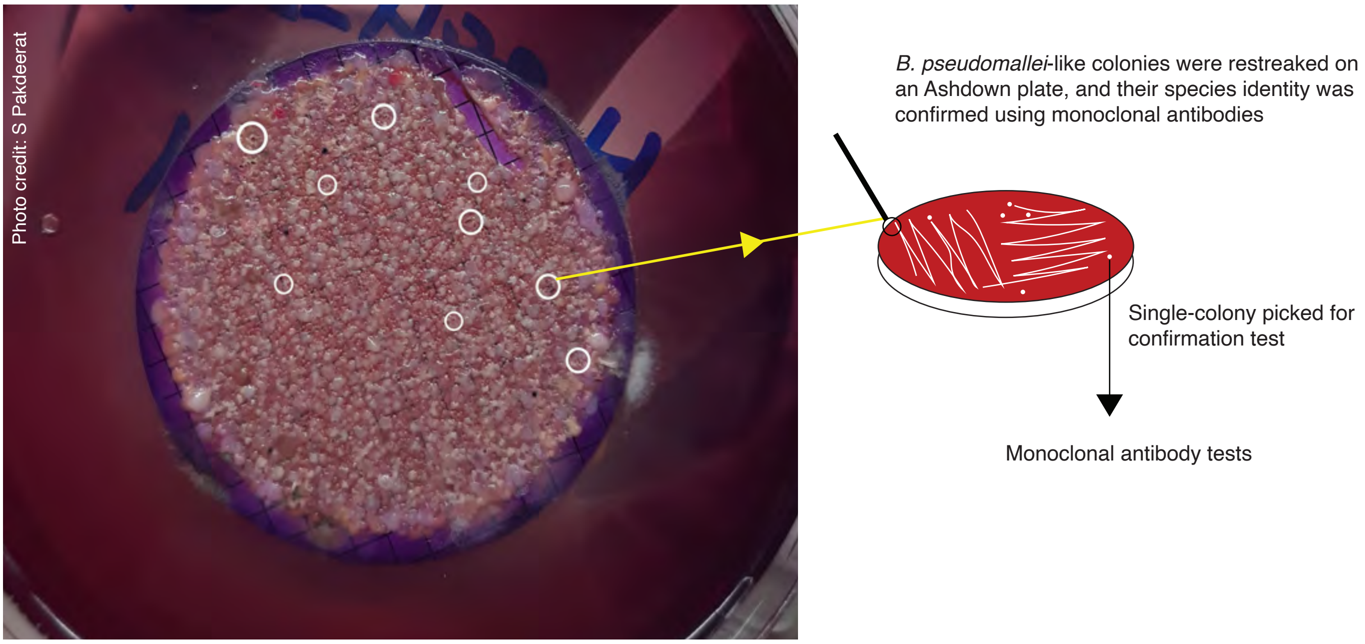

c New plate-sweep method followed by species confirmation with PCR or CRISPR-BEEPs

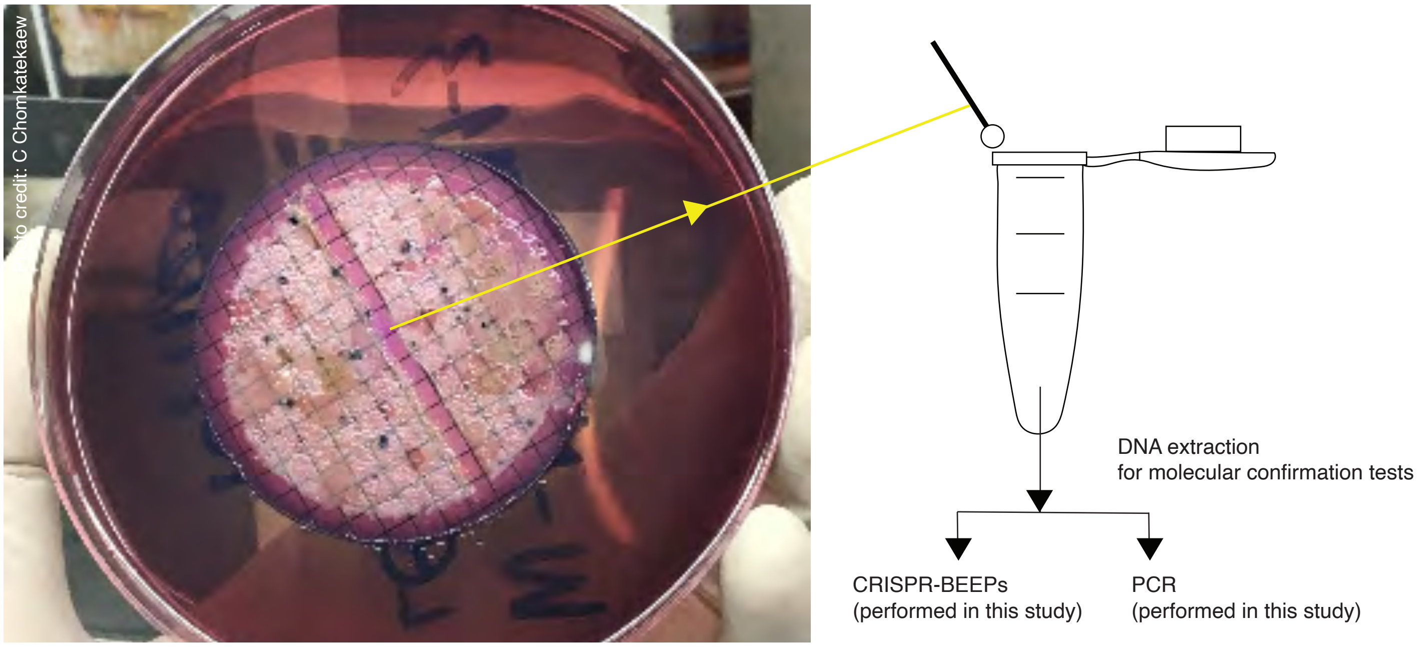

Supplementary Figure 5 Participants with multiple water samples collected.
Participants with more than one water sample collected from their household and surrounding environment included melioidosis cases (n = 23), other bacterial infection controls (n = 30), and healthy controls (n=26). (a) Temporal distribution of collected water samples by participant group, presented from left to right: healthy controls, other infectious controls, and melioidosis cases. Each row represents an individual participant, with dots indicating the collected water samples; the size of each dot is proportional to the sample size. (b) *B. pseudomallei* positivity rates in water samples collected from participants across different seasons, categorised by case and control groups. The upper arc represents the dry season, while the lower arc indicates the rainy/flood season. In both (a) and (b), colour coding reflects the positivity of *B. pseudomallei* in water samples, as determined by double-qPCR assays. Source Data are provided as Source Data file.

Supplementary Figure 5

a participants with multiple samples collected and the seasonal fluctuation in positive *B. pseudomallei* samples

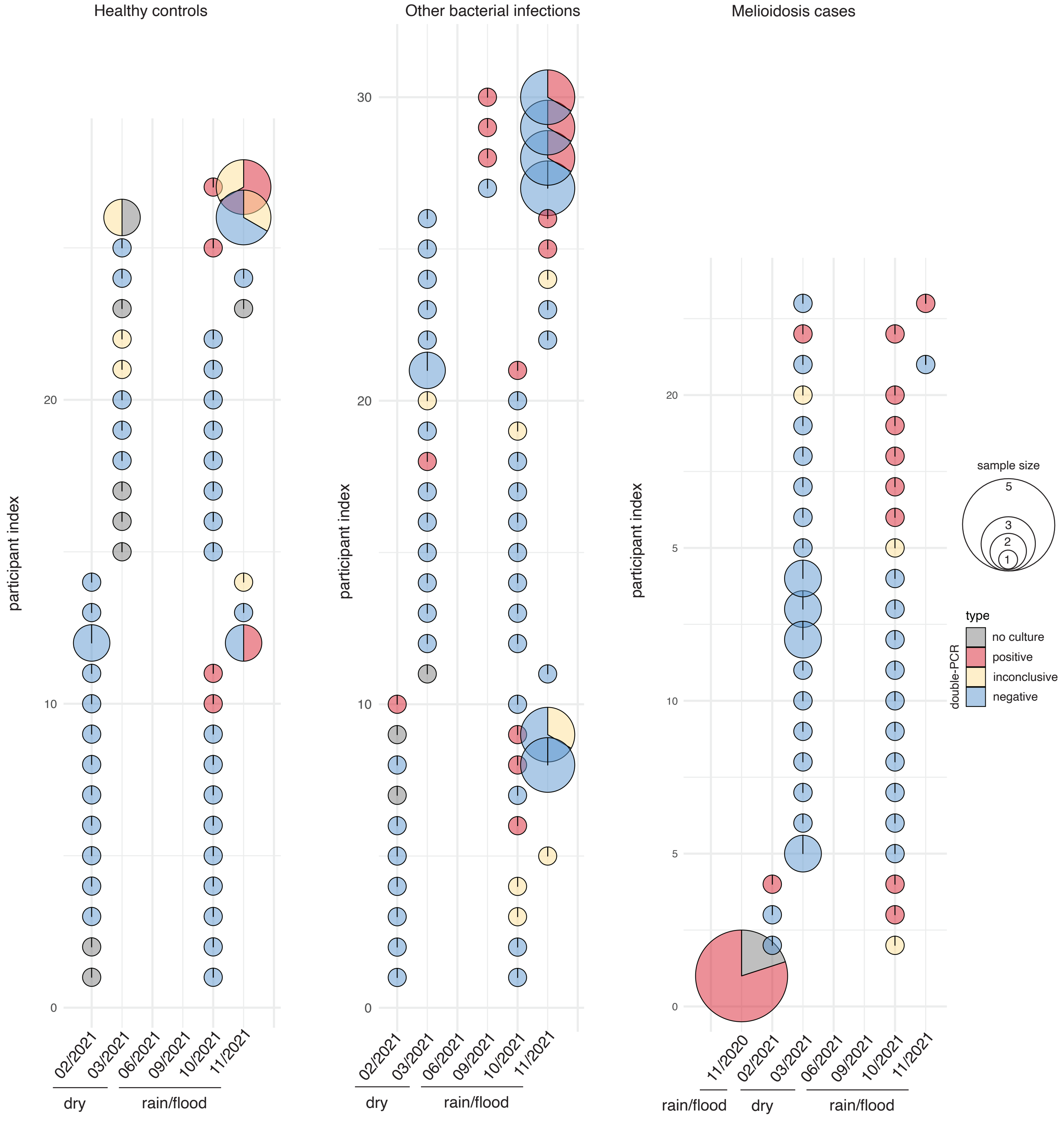

b Changes in *B. pseudomallei* positivity rate between the dry and rainy/flood seasons across cohort

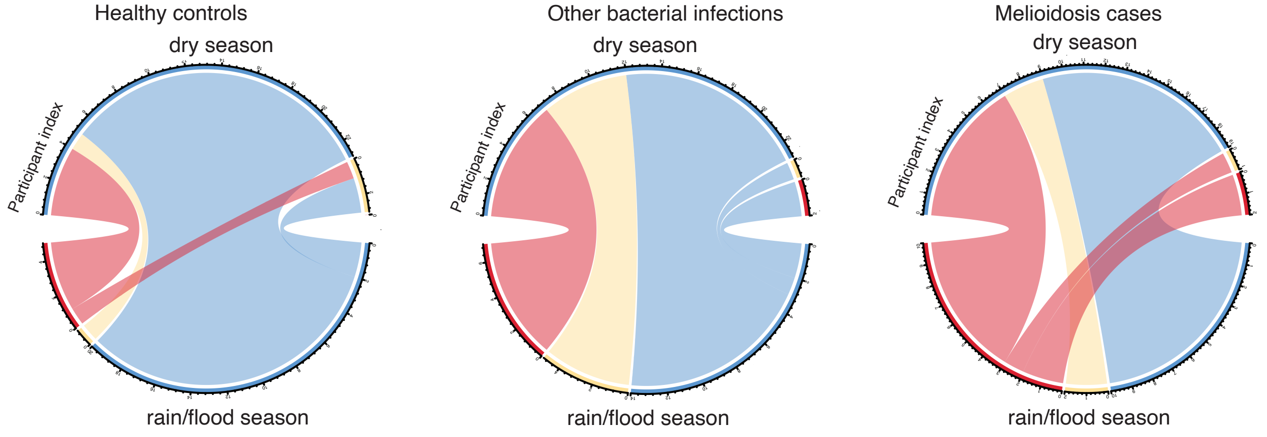

Supplementary Figure 6 Efficiency, coverage, cycle threshold (ct) scores of PCR primers used in this study.

(a) *In silico* PCR analysis demonstrating the detailed coverage of primer 1 (*TTS1*) and primer 2 (*BPSS1386*) across the *B. pseudomallei* genomic population (n = 3,341 genomes). (b) Linear regression analysis used to calculate primer efficiencies: *TTS1* (96.1%) and *BPSS1386* (81.3%), determined using a formula in reference[28]. (c) Cycle threshold (Ct) values from technical replicates (n = 2) for each primer set. Black points indicate experiments with correct melting temperatures, while grey points represent those with incorrect melting temperatures, where non-specific primer binding may occur. Source Data are provided as Source Data file.

Supplementary Figure 6

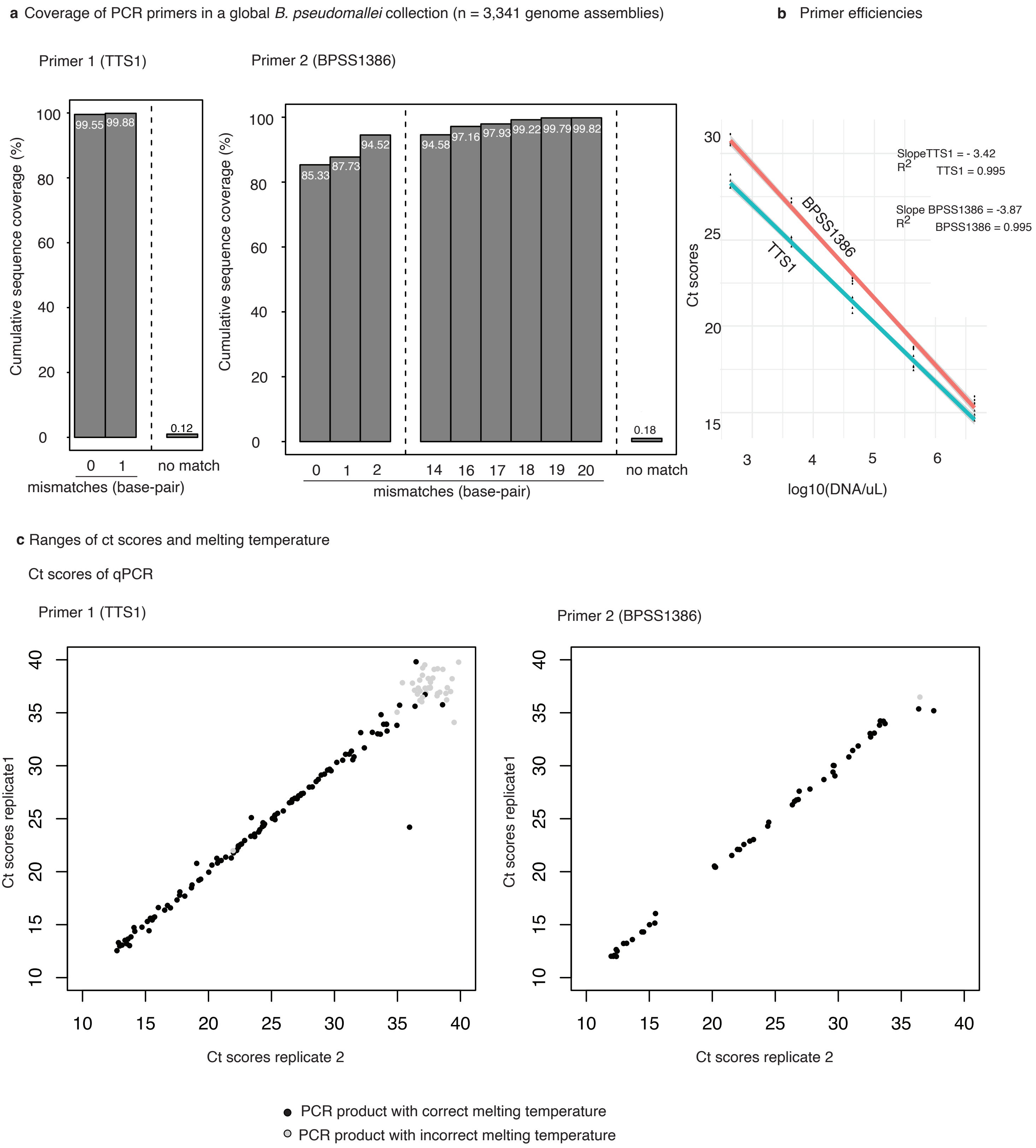

SUPPLEMENTARY TABLES

Supplementary Table 1: A list of primers and oligos used in this study

A list of primers and oligonucleotides used for recombinase polymerase amplification (RPA), qPCR assays targeting *Burkholderia pseudomallei*, and CRISPR-based detection. Sequences are provided in the 5'-3' orientation. RPA primers were designed to amplify the crBP34 target region, while PCR primers target genes within the type III secretion system cluster 1 (*TSS1*) and BPSS1386. A FAM-biotin-labelled probe was used as a collateral reporter in CRISPR-based detection assays. References indicate the original source of each sequence.

ID	Sequences	Usage	Reference
148	CAGCATATCATTGTCCGGCGCGAACCATCAAGCTA	RPA primer for *crBP34*	[2]
149	AACTTTTCATTTTCCTGTCAATTCGACTGACCATC	RPA primer for *crBP34*	[2]
167	TAATACGACTCACTATAGGGTAATTTCTACTAAGTG TAGATACTACATACCCACTATTCAGAAAGGAA	Annealing to DNA oligo 167 to create a T7transcription template for *crBP34* crRNA	[2]
168	TTCCTTTCTGAATAGTGGGTATGTAGTATCTACACT TAGTAGAAATTACCCTATAGTGAGTCGTATTA	Annealing to DNA oligo 167 to create a T7transcription template for *crBP34* crRNA	[2]
192	CGTCTCTATACTGTCGAGCAATCG	PCR primer targeting orf2 within putative type III secretion system cluster 1 (*TTS1*) of *B. pseudomallei*	[29]
193	CGTGCACACCGGTCAGTATC	PCR primer targeting orf2 within putative type III secretion system cluster 1 (*TTS1*) of *B. pseudomallei*	[29]
270	AACACTGACAAGTGGCCCTATGGA	PCR primer targeting orf11 (*BPSS1386*) of *B. pseudomallei*	[30]
271	TCCGATCGGTTTCGAATAACGGGT	PCR primer targeting orf11 (*BPSS1386*) of *B. pseudomallei*	[30]
FAM-biotin probe	/56-FAM/TTATT/3Bio/	Collateral probe	[2,3]

Supplementary Table 2: Seasonal fluctuations in *B. pseudomallei* positivity detected by molecular and conventional methods

Water samples from different sources (piped water, surface water, and boreholes) were screened using double-qPCR, CRISPR-BEEPs, and conventional plate inspection. Results are presented as the number of positive, inconclusive (where applicable), and negative samples, with corresponding positivity rates. Comparisons between dry and rain/flood seasons were performed using chi-square tests. P-values are reported to assess seasonal differences in detection rates for each method and water type. Borehole samples were only available during the rain/flood season. “NA” or “ - ” indicates data not available or not applicable.

Water types	Screening methods	Dry season				Rain/flood season				Chi-sq test comparing dry vs rain/flood season (p-value)
		N positive samples	N Inconclusive samples	N negative samples	Positive rate (%)	N positive samples	N Inconclusive samples	N negative samples	Positive rate (%)	
Piped water	Double-qPCR	4	5	54	4/63 (6.3%)	37	4	41	37/82 (45.1%)	1.84×10^{-6}
	CRISPR-BEEPs	5	NA	58	5/63 (7.9%)	33	NA	49	33/82 (40.2%)	2.73×10^{-5}
	Conventional plate inspection	0	NA	63	0/63 (0.0%)	9	NA	73	9/82 (11.0%)	0.018
Surface water (ponds, lakes, canals and rivers)	Double-qPCR	0	0	23	0/23 (0.0%)	56	17	77	56/150 (37.3%)	6.24×10^{-5}
	CRISPR-BEEPs	0	-	23	0/23 (0.0%)	57	-	93	57/150 (38.0%)	7.46×10^{-4}
	Conventional plate inspection	0	-	23	0/23 (0.0%)	14	-	136	14/150 (9.3%)	0.264
Boreholes	Double-qPCR	no data	no data	no data	NA	11	1	3	11/15 (73.3%)	NA
	CRISPR-BEEPs	no data	no data	no data	NA	11	-	4	11/15 (73.3%)	NA
	Conventional plate inspection	no data	no data	no data	NA	4	-	11	4/15 (26.7%)	NA
Overall	Double-qPCR	4	5	77	4/86 (4.7%)	104	22	121	104/247 (42.1%)	1.04×10^{-10}
	CRISPR-BEEPs	5	-	81	5/86 (5.8%)	101	-	146	101/247 (40.9%)	4.11×10^{-9}
	Conventional plate inspection	0	-	86	0/86 (0.0%)	27	-	220	27/247 (10.9%)	0.003

Supplementary Table 3: Household-level detection of *B. pseudomallei* by molecular and conventional methods and its association with melioidosis in residents

The proportion of households with at least one water sample testing positive for *B. pseudomallei* is shown for melioidosis households, households with other infections, and healthy control households. Detection was performed using double-qPCR, CRISPR-BEEPs, and conventional plate inspection. Positivity is expressed as the number of positive households over the total number tested, with corresponding percentages. Differences in positivity between household groups were assessed using two-tailed Fisher's exact tests, and p-values are reported for pair-wise comparisons.

Detection methods	Water samples tested positive for *B. pseudomallei* at least once (%)			P-value from two-tailed Fisher's exact test comparing differences in *B. pseudomallei* positivity between households		
	Melioidosis household (n = 70)	Other infection household (n = 69)	Healthy control household (n = 104)	Melioidosis vs Healthy	Melioidosis vs Other infection	Other infection vs Healthy
Double-qPCR	34/70 (48.6%)	30/69 (43.5%)	34/104 (32.7%)	0.04	0.61	0.20
CRISPR-BEEPs	34/70 (48.6%)	30/69 (43.5%)	33/104 (31.7%)	0.03	0.61	0.15
Conventional plate inspection	7/70 (10.0%)	11/69 (15.9%)	6/104 (5.7%)	0.38	0.33	0.04

Supplementary Table 4: Demographic characteristics of the studied population

Participant characteristics are summarised for melioidosis cases, other infectious disease controls, and healthy controls. Continuous variables are presented as median with interquartile range (IQR), and categorical variables as number (percentage). Variables include age, sex, self-reported ethnicity, body-mass index (BMI), glycaemic status based on HbA1c, occupation, and recent flood exposure. BMI and HbA1c are categorized using standard clinical thresholds. Totals may vary slightly due to missing data, as indicated.

	**Melioidosis cases (n=439 or stated otherwise)**	**Other infectious controls (n=190 or stated otherwise)**	**Healthy controls (n=506 or stated otherwise)**
**Participant characteristics**			
Age, median years (IQR)	54 (45-62)	60 (52-69)	50 (45-58)
Age groups, years (%)			
18-30	22 (5%)	7 (4%)	0
31-50	154 (35%)	36 (19%)	275 (54%)
51-70	225 (51%)	110 (58%)	214 (42%)
>70	37 (8%)	36 (19%)	17 (3%)
Sex (%)			
Female	127 (29%)	69 (37%)	237 (47%)
Male	311 (71%)	120 (63%)	269 (53%)
Self-reported ethnicity (%)			
Thai	436 (>99%)	187 (99%)	504 (>99%)
Lao	2 (<1%)	2 (1%)	1 (<1%)
Cambodian	0	0	1 (<1%)
**Participant underlying health conditions**			
Body-mass index, median BMI scores (IQR)	22 (20-25)	22 (20-26)	25 (23-28)
Body-mass index, BMI groups (%)			
Underweight (<18.5)	52 (12%)	23 (12%)	13 (3%)
Healthy (18.5 – 22.9)	192 (44%)	78 (41%)	109 (22%)
Overweight (23.0 – 26.5)	139 (32%)	51 (27%)	220 (43%)
Obese (≥ 27.0)	55 (12%)	37 (19%)	164 (32%)
unknown	1 (<1%)	1 (<1%)	0
Blood glucose level, HbA1c (IQR)	8.65 (6.00-12.38)	5.90 (5.10-7.68)	6.45 (5.50-8.70)
Blood glucose level, groups			
Non-diabetic (<5.7)	88 (20%)	87 (46%)	186 (37%)
Prediabetic (5.7 – 6.4)	48 (11%)	31 (16%)	67 (13%)
Diabetic (≥ 6.5)	298 (68%)	72 (38%)	253 (50%)
unknown	5 (1%)	0	0
**Participant occupational and behavioural risks**			

Occupation (%)			
Agriculture and/or fisheries	277 (63%)	68 (36%)	127 (25%)
Homemaker and/or retirees	62 (14%)	86 (46%)	122 (24%)
Private sector	55 (13%)	16 (8%)	128 (25%)
Merchant	15 (3%)	11 (6%)	59 (12%)
Other	29 (7%)	8 (4%)	69 (14%)
Exposure to flood in the past (%)			
True	229 (52%)	151 (80%)	327 (65%)
False	209 (48%)	38 (20%)	177 (35%)

Supplementary Table 5: Univariate logistic regression of factors associated with melioidosis

Univariable logistic regression was performed to assess the association between demographic characteristics, underlying health conditions, and occupational and environmental exposures with melioidosis risk. The analysis included 1,132 participants. Odds ratios (ORs) with 95% confidence intervals (CIs) are reported. P-values are presented both unadjusted and after Bonferroni correction for multiple comparisons. Reference categories are indicated for categorical variables.

	**Odds ratio (95% CI)**	**Non-adjust p-value**	**Bonferroni adjusted p-value**
**Participants demographic**			
Age	1.00 (0.99-1.01)	0.424	1.000
Sex			
Male (baseline)	1		
Female	0.53 (0.41-0.70)	4.68×10^{-7}	4.212×10^{-6}
Self-reported ethnicity			
Thai (baseline)	1		
Others	0.70 (0.11-2.76)	0.970	1.000
**Participants underlying health conditions**			
Body-mass index (BMI)	0.88 (0.85-0.91)	3.75×10^{-16}	3.375×10^{-15}
Blood glucose level (HbA1c)	1.22 (1.17-1.28)	$< 2 \times 10^{-16}$	1.800×10^{-15}
**Participants occupational and exposure risks**			
Occupation			
Homemaker and/or retirees (baseline)	1		
Agriculture and/or fisheries	5.48 (3.86-7.86)	$< 2 \times 10^{-16}$	1.800×10^{-15}
Private sector	1.28 (0.82-2.00)	0.248	1.000
Merchant	0.71 (0.35-1.35)	0.280	1.000
Other	1.27 (0.74-2.16)	0.371	1.000
Exposure to flood in the past 6 months			
False (baseline)	1		
True	2.02 (1.56-2.63)	1.99×10^{-8}	1.791×10^{-7}

Supplementary Table 6: Multivariable logistic regression of factors associated with melioidosis based on *B. pseudomallei* detection within 10 km

Multivariable logistic regression was used to identify factors associated with melioidosis risk based on *B. pseudomallei* detection within a 10 km radius of participant households. The analysis included 1,029 participants, 243 of whom had direct environmental sampling data. Odds ratios (ORs) with 95% confidence intervals (CIs) are reported. The test was two-sided, and reference categories are indicated for categorical variables.

	Risk of developing melioidosis					
	Environmental screening performed by conventional approach		Environmental screening performed by CRISPR-BEEPs		Environmental screening performed by double-qPCR	
	Odds ratio (95% CI)	p-value	Odds ratio (95% CI)	p-value	Odds ratio (95% CI)	p-value
**Participants demographic**						
Age	0.99 (0.98-1.00)	0.174074	0.99 (0.98-1.00)	0.178617	0.99 (0.98-1.00)	0.181014
Sex						
Male (baseline)	1		1		1	
Female	0.57 (0.41-0.78)	0.000667	0.56 (0.41-0.78)	0.000641	0.56 (0.40-0.78)	0.000574
Self-reported ethnicity						
Thai (baseline)	1		1		1	
Others	0.56 (0.07-2.69)	0.511618	0.55 (0.07-2.67)	0.507336	0.54 (0.06-2.57)	0.481291
**Participants underlying health conditions**						
Body-mass index (BMI)	0.88 (0.84-0.91)	9.51×10^{-12}	0.88 (0.84-0.91)	1.58×10^{-11}	0.88 (0.84-0.91)	1.06×10^{-11}
Blood glucose level (HbA1c)	1.25 (1.19-1.32)	$< 2 \times 10^{-16}$	1.25 (1.19-1.31)	$< 2 \times 10^{-16}$	1.25 (1.19-1.31)	$< 2 \times 10^{-16}$
**Participants occupational and exposure risks**						
Occupation						
Homemaker and/or retirees (baseline)	1		1		1	
Agriculture and/or fisheries	4.66 (3.05-7.21)	2.27×10^{-12}	4.47 (2.92-6.93)	9.82×10^{-12}	4.46 (2.91-6.91)	1.20×10^{-11}
Private sector	1.22 (0.72-2.07)	0.463509	1.24 (0.73-2.11)	0.426860	1.25 (0.73-2.12)	0.418462
Merchant	0.68 (0.31-1.41)	0.325225	0.71 (0.32-1.47)	0.373195	0.70 (0.32-1.45)	0.3
Other	1.18 (0.63-2.20)	0.603167	1.21 (0.64-2.27)	0.556256	1.23 (0.65-2.30)	0.528693
Exposure to flood in the past 6 months						
False (baseline)	1		1		1	
True	1.10 (0.79-1.52)	0.587988	1.09 (0.78-1.52)	0.602743	1.09 (0.78-1.51)	0.623046
B. pseudomallei positivity rates in water samples within 10 km from the household	1.03 (0.28-3.79)	0.969096	2.56 (1.31-5.04)	0.006379	2.74 (1.38-5.48)	0.004191

Supplementary Table 7: Multivariable logistic regression of factors associated with melioidosis based on *B. pseudomallei* detection within 9 km

Multivariable logistic regression was used to identify factors associated with melioidosis risk based on *B. pseudomallei* detection within a 9 km radius of participant households. The analysis included 1,014 participants, 243 of whom had direct environmental sampling data. Odds ratios (ORs) with 95% confidence intervals (CIs) are reported. The test was two-sided, and reference categories are indicated for categorical variables.

	**Environmental screening performed by conventional approach**		**Environmental screening performed by CRISPR-BEEPs**		**Environmental screening performed by double-qPCR**	
	Odds ratio (95% CI)	p-value	Odds ratio (95% CI)	p-value	Odds ratio (95% CI)	p-value
**Participants demographic**						
Age	0.99 (0.98-1.00)	0.190387	0.99 (0.98-1.01)	0.214785	0.99 (0.98-1.00)	0.222943
Sex						
Male (baseline)	1		1		1	
Female	0.55 (0.40-0.77)	0.000463	0.55 (0.39-0.77)	0.000421	0.55 (0.39-0.76)	0.000405
Self-reported ethnicity						
Thai (baseline)	1		1		1	
Others	0.56 (0.07-2.73)	0.521354	0.57 (0.07-2.73)	0.525206	0.56 (0.07-2.66)	0.505412
**Participants underlying health conditions**						
Body-mass index (BMI)	0.88 (0.85-0.91)	3.46×10^{-11}	0.88 (0.85-0.91)	3.72×10^{-11}	0.88 (0.84-0.91)	2.72×10^{-11}
Blood glucose level (HbA1c)	1.26 (1.20-1.32)	$< 2 \times 10^{-16}$	1.25 (1.19-1.32)	$< 2 \times 10^{-16}$	1.26 (1.20-1.32)	$< 2 \times 10^{-16}$
**Participants occupational and exposure risks**						
Occupation						
Homemaker and/or retirees (baseline)	1		1		1	
Agriculture and/or fisheries	4.92 (3.20-7.66)	7.57×10^{-13}	4.77 (3.10-7.44)	2.59×10^{-12}	4.73 (3.07-7.39)	3.60×10^{-12}
Private sector	1.30 (0.76-2.23)	0.334030	1.33 (0.77-2.27)	0.303812	1.33 (0.78-2.28)	0.300432
Merchant	0.67 (0.29-1.43)	0.316695	0.69 (0.30-1.48)	0.360771	0.69 (0.30-1.47)	0.352503
Other	1.21 (0.63-2.28)	0.557157	1.23 (0.64-2.24)	0.520100	1.25 (0.65-2.47)	0.498600
Exposure to flood in the past 6 months						
False (baseline)	1		1		1	
True	1.11 (0.80-1.55)	0.516913	1.13 (0.81-1.58)	0.456914	1.13 (0.81-1.58)	0.461546
B. pseudomallei positivity rates in water samples within 9 km from the household	1.10 (0.32-3.81)	0.885515	1.87 (1.01-3.50)	0.047856	2.11 (1.11-4.05)	0.023525

Supplementary Table 8 Multivariable logistic regression of factors associated with melioidosis based on *B. pseudomallei* detection within 8 km

Multivariable logistic regression was used to identify factors associated with melioidosis risk based on *B. pseudomallei* detection within a 8 km radius of participant households. The analysis included 994 participants, 243 of whom had direct environmental sampling data. Odds ratios (ORs) with 95% confidence intervals (CIs) are reported. The test was two-sided, and reference categories are indicated for categorical variables.

	**Risk of developing melioidosis**					
	**Environmental screening performed by conventional approach**		**Environmental screening performed by CRISPR-BEEPs**		**Environmental screening performed by double-qPCR**	
	Odds ratio (95% CI)	p-value	Odds ratio (95% CI)	p-value	Odds ratio (95% CI)	p-value
**Participants demographic**						
Age	0.99 (0.98-1.01)	0.198914	0.99 (0.98-1.01)	0.233260	0.99 (0.98-1.00)	0.225539
Sex						
Male (baseline)	1		1		1	
Female	0.56 (0.40-0.78)	0.000645	0.55 (0.40-0.77)	0.000578	0.55 (0.39-0.77)	0.000519
Self-reported ethnicity						
Thai (baseline)	1		1		1	
Others	0.54 (0.06-2.63)	0.499526	0.57 (0.07-2.74)	0.527856	0.56 (0.07-2.68)	0.510741
**Participants underlying health conditions**						
Body-mass index (BMI)	0.88 (0.84-0.91)	1.96×10^{-11}	0.88 (0.84-0.91)	1.97×10^{-11}	0.88 (0.84-0.91)	1.57×10^{-11}
Blood glucose level (HbA1c)	1.26 (1.20-1.33)	$< 2 \times 10^{-16}$	1.26 (1.20-1.32)	$< 2 \times10^{-16}$	1.26 (1.20-1.32)	$< 2 \times 10^{-16}$
**Participants occupational and exposure risks**						
Occupation						
Homemaker and/or retirees (baseline)	1		1		1	
Agriculture and/or fisheries	4.80 (3.12-7.49)	2.32×10^{-12}	4.70 (3.05-7.35)	5.07×10^{-12}	4.64 (3.01-7.26)	8.33×10^{-12}
Private sector	1.23 (0.72-2.12)	0.445902	1.25 (0.73-2.16)	0.412231	1.26 (0.73-2.16)	0.412387
Merchant	0.67 (0.29-1.43)	0.316703	0.68 (0.30-1.46)	0.337436	0.67 (0.29-1.44)	0.325887
Other	1.28 (0.66-2.42)	0.457344	1.29 (0.67-2.45)	0.434577	1.30 (0.68-2.46)	0.428710
Exposure to flood in the past 6 months						
False (baseline)	1		1		1	
True	1.12 (0.81-1.59)	0.452918	1.17 (0.83-1.63)	0.370629	1.17 (0.83-1.63)	0.367047
B. pseudomallei positivity rates in water samples within 8 km from the household	2.33 (0.80-6.83)	0.120782	1.67 (0.93-3.00)	0.088697	1.96 (1.07-3.61)	0.029926

Supplementary Table 9 Multivariable logistic regression of factors associated with melioidosis based on *B. pseudomallei* detection within 7 km

Multivariable logistic regression was used to identify factors associated with melioidosis risk based on *B. pseudomallei* detection within a 7 km radius of participant households. The analysis included 968 participants, 243 of whom had direct environmental sampling data. Odds ratios (ORs) with 95% confidence intervals (CIs) are reported. The test was two-sided, and reference categories are indicated for categorical variables.

	**Risk of developing melioidosis**					
	**Environmental screening performed by conventional approach**		**Environmental screening performed by CRISPR-BEEPS**		**Environmental screening performed by double-qPCR**	
	Odds ratio (95% CI)	p-value	Odds ratio (95% CI)	p-value	Odds ratio (95% CI)	p-value
**Participants demographic**						
Age	0.99 (0.98-1.01)	0.357205	0.99 (0.98-1.01)	0.3835	0.99 (0.98-1.01)	0.383697
Sex						
Male (baseline)	1		1		1	
Female	0.55 (0.39-0.78)	0.000657	0.55 (0.39-0.77)	0.0006	0.55 (0.39-0.77)	0.000536
Self-reported ethnicity						
Thai (baseline)	1		1		1	
Others	0.58 (0.07-2.79)	0.539110	0.59 (0.07-2.86)	0.5557	0.59 (0.07-2.81)	0.545057
**Participants underlying health conditions**						
Body-mass index (BMI)	0.88 (0.84-0.91)	5.77×10^{-11}	0.88 (0.84-0.91)	5.36×10^{-11}	0.88 (0.84-0.91)	4.62×10^{-11}
Blood glucose level (HbA1c)	1.27 (1.20-1.33)	$< 2 \times 10^{-16}$	1.26 (1.20-1.33)	$< 2 \times 10^{-16}$	1.26 (1.20-1.33)	$< 2 \times 10^{-16}$
**Participants occupational and exposure risks**						
Occupation						
Homemaker and/or retirees (baseline)	1		1		1	
Agriculture and/or fisheries	5.12 (3.30-8.96)	7.74×10^{-13}	5.05 (3.35-7.96)	1.26×10^{-12}	5.01 (3.22-7.91)	1.81×10^{-12}
Private sector	1.29 (0.74-2.22)	0.369767	1.30 (0.75-2.25)	0.3538	1.30 (0.75-2.26)	0.346964
Merchant	0.70 (0.30-1.50)	0.375792	0.71 (0.31-1.52)	0.3929	0.71 (0.31-1.53)	0.397542
Other	1.33 (0.69-2.55)	0.390633	1.36 (0.70-2.61)	0.3565	1.39 (0.71-2.67)	0.326876
Exposure to flood in the past 6 months						
False (baseline)	1		1		1	
True	1.06 (0.75-1.50)	0.724133	1.07 (0.76-1.51)	0.6798	1.07 (0.76-1.50)	0.706172
B. pseudomallei positivity rates in water samples within 7 km from the household	1.50 (0.52-4.42)	0.452042	1.46 (0.85-2.52)	0.1668	1.91 (1.09-3.34)	0.023592

Supplementary Table 10 Multivariable logistic regression of factors associated with melioidosis based on *B. pseudomallei* detection within 6 km

Multivariable logistic regression was used to identify factors associated with melioidosis risk based on *B. pseudomallei* detection within a 6 km radius of participant households. The analysis included 934 participants, 243 of whom had direct environmental sampling data. Odds ratios (ORs) with 95% confidence intervals (CIs) are reported. The test was two-sided, and reference categories are indicated for categorical variables.

	**Risk of developing melioidosis**					
	**Environmental screening performed by conventional approach**		**Environmental screening performed by CRISPR-BEEPS**		**Environmental screening performed by double-qPCR**	
	Odds ratio (95% CI)	p-value	Odds ratio (95% CI)	p-value	Odds ratio (95% CI)	p-value
**Participants demographic**						
Age	1.00 (0.98-1.01)	0.59022	1.00 (0.98-1.01)	0.62136	1.00 (0.98-1.01)	0.61276
Sex						
Male (baseline)	1		1		1	
Female	0.57 (0.40-0.81)	0.00169	0.57 (0.40-0.80)	0.00156	0.56 (0.40-0.80)	0.00148
Self-reported ethnicity						
Thai (baseline)	1		1		1	
Others	0.59 (0.07-2.91)	0.56358	0.61 (0.07-2.98)	0.58136	0.60 (0.07-2.92)	0.56636
**Participants underlying health conditions**						
Body-mass index (BMI)	0.88 (0.85-0.92)	3.44×10^{-10}	0.88 (0.85-0.92)	3.68×10^{-10}	0.88 (0.85-0.92)	3.56×10^{-10}
Blood glucose level (HbA1c)	1.27 (1.20-1.33)	$< 2 \times 10^{-16}$	1.26 (1.20-1.33)	$< 2 \times 10^{-16}$	1.26 (1.20-1.33)	$< 2 \times 10^{-16}$
**Participants occupational and exposure risks**						
Occupation						
Homemaker and/or retirees (baseline)	1		1		1	
Agriculture and/or fisheries	5.69 (3.61-9.11)	1.65×10^{-13}	5.64 (3.58-9.03)	2.22×10^{-13}	5.59 (3.55-8.96)	3.08×10^{-13}
Private sector	1.39 (0.79-2.45)	0.25176	1.40 (0.79-2.46)	0.24387	1.39 (0.79-2.45)	0.24758
Merchant	0.73 (0.31-1.63)	0.46832	0.74 (0.31-1.65)	0.48397	0.74 (0.31-1.64)	0.48085
Other	1.32 (0.66-2.61)	0.42373	1.34 (0.67-2.63)	0.40532	1.34 (0.67-2.64)	0.40292
Exposure to flood in the past 6 months						
False (baseline)	1		1		1	
True	1.08 (0.76-1.54)	0.65373	1.09 (0.76-1.55)	0.63617	1.09 (0.76-1.54)	0.64488
B. pseudomallei positivity rates in water samples within 6 km from the household	1.25 (0.41-3.78)	0.68998	1.30 (0.78-2.16)	0.31929	1.45 (0.86-2.46)	0.16586

Supplementary Table 11 Multivariable logistic regression of factors associated with melioidosis based on *B. pseudomallei* detection within 5 km

Multivariable logistic regression was used to identify factors associated with melioidosis risk based on *B. pseudomallei* detection within a 5 km radius of participant households. The analysis included 868 participants, 243 of whom had direct environmental sampling data. Odds ratios (ORs) with 95% confidence intervals (CIs) are reported. The test was two-sided, and reference categories are indicated for categorical variables.

	**Risk of developing melioidosis**					
	**Environmental screening performed by conventional approach**		**Environmental screening performed by CRISPR-BEEPS**		**Environmental screening performed by double-qPCR**	
	Odds ratio (95% CI)	p-value	Odds ratio (95% CI)	p-value	Odds ratio (95% CI)	p-value
**Participants demographic**						
Age	1.00 (0.98-1.01)	0.57264	1.00 (0.98-1.01)	0.62158	1.00 (0.98-1.01)	0.60435
Sex						
Male (baseline)	1		1		1	
Female	0.58 (0.40-0.82)	0.00326	0.57 (0.39-0.82)	0.00272	0.57 (0.39-0.82)	0.00253
Self-reported ethnicity						
Thai (baseline)	1		1		1	
Others	0.58 (0.07-2.84)	0.54259	0.59 (0.07-2.90)	0.55850	0.60 (0.07-2.91)	0.56211
**Participants underlying health conditions**						
Body-mass index (BMI)	0.89 (0.85-0.92)	7.15×10^{-9}	0.89 (0.85-0.92)	7.92×10^{-9}	0.89 (0.85-0.92)	7.29×10^{-9}
Blood glucose level (HbA1c)	1.26 (1.19-1.33)	$< 2 \times 10^{-16}$	1.26 (1.19-1.33)	$< 2 \times 10^{-16}$	1.26 (1.19-1.33)	$< 2 \times 10^{-16}$
**Participants occupational and exposure risks**						
Occupation						
Homemaker and/or retirees (baseline)	1		1		1	
Agriculture and/or fisheries	6.04 (3.77-9.82)	1.66×10^{-13}	5.99 (3.74-9.75)	2.10×10^{-13}	5.94 (3.71-9.67)	2.93×10^{-13}
Private sector	1.30 (0.73-2.32)	0.37680	1.31 (0.73-2.34)	0.36069	1.31 (0.73-2.34)	0.36686
Merchant	0.76 (0.32-1.69)	0.52055	0.78 (0.33-1.74)	0.56163	0.78 (0.33-1.75)	0.56596
Other	1.33 (0.65-2.68)	0.42166	1.36 (0.67-2.73)	0.39291	1.37 (0.67-2.76)	0.38219
Exposure to flood in the past 6 months						
False (baseline)	1		1		1	
True	1.11 (0.76-1.60)	0.58213	1.12 (0.77-1.61)	0.55718	1.12 (0.76-1.65)	0.56021
B. pseudomallei positivity rates in water samples within 5 km from the household	1.24 (0.38-4.01)	0.71833	1.41 (0.83-2.40)	0.20019	1.64 (0.96-2.81)	0.07151

Supplementary Table 12 Multivariable logistic regression of factors associated with melioidosis based on *B. pseudomallei* detection within 4 km

Multivariable logistic regression was used to identify factors associated with melioidosis risk based on *B. pseudomallei* detection within a 4 km radius of participant households. The analysis included 801 participants, 243 of whom had direct environmental sampling data. Odds ratios (ORs) with 95% confidence intervals (CIs) are reported. The test was two-sided, and reference categories are indicated for categorical variables.

	Risk of developing melioidosis					
	Environmental screening performed by conventional approach		Environmental screening performed by CRISPR-BEEPS		Environmental screening performed by double-qPCR	
	Odds ratio (95% CI)	p-value	Odds ratio (95% CI)	p-value	Odds ratio (95% CI)	p-value
**Participants demographic**						
Age	0.99 (0.97-1.01)	0.2200	0.99 (0.97-1.01)	0.23745	0.99 (0.97-1.01)	0.22812
Sex						
Male (baseline)	1		1		1	
Female	0.60 (0.41-0.88)	0.0096	0.59 (0.40-0.87)	0.00857	0.66 (0.40-0.88)	0.00895
Self-reported ethnicity						
Thai (baseline)	1		1		1	
Others	0.60 (0.07-2.95)	0.5674	0.60 (0.07-2.94)	0.56941	0.60 (0.07-2.93)	0.56589
**Participants underlying health conditions**						
Body-mass index (BMI)	0.88 (0.84-0.92)	8.19×10^{-9}	0.88 (0.85-0.92)	9.22×10^{-9}	0.88 (0.84-0.92)	8.38×10^{-9}
Blood glucose level (HbA1c)	1.27 (1.20-1.34)	$< 2 \times 10^{-16}$	1.27 (1.20-1.34)	$< 2 \times 10^{-16}$	1.27 (1.20-1.34)	$< 2 \times 10^{-16}$
**Participants occupational and exposure risks**						
Occupation						
Homemaker and/or retirees (baseline)	1		1		1	
Agriculture and/or fisheries	5.46 (3.35-9.08)	2.40×10^{-11}	5.41 (3.32-9.02)	2.87×10^{-11}	5.43 (3.32-9.02)	3.00×10^{-11}
Private sector	1.23 (0.67-2.26)	0.4931	1.25 (0.68-2.29)	0.46410	1.25 (0.68-2.28)	0.40992
Merchant	0.69 (0.28-1.59)	0.4011	0.70 (0.28-1.62)	0.42509	0.70 (0.28-1.60)	0.41496
Other	1.32 (0.65-2.72)	0.4276	1.35 (0.65-2.75)	0.41109	1.35 (0.65-2.75)	0.47293
Exposure to flood in the past 6 months						
False (baseline)	1		1		1	
True	1.12 (0.76-1.65)	0.5626	1.12 (0.76-1.65)	0.56836	1.12 (0.76-1.65)	0.57336
B. pseudomallei positivity rates in water samples within 4 km from the household	0.98 (0.28-3.33)	0.9787	1.29 (0.75-2.21)	0.35038	1.24 (0.72-2.14)	0.43303

Supplementary Table 13 Multivariable logistic regression of factors associated with melioidosis based on *B. pseudomallei* detection within 3 km

Multivariable logistic regression was used to identify factors associated with melioidosis risk based on *B. pseudomallei* detection within a 3 km radius of participant households. The analysis included 679 participants, 243 of whom had direct environmental sampling data. Odds ratios (ORs) with 95% confidence intervals (CIs) are reported. The test was two-sided, and reference categories are indicated for categorical variables.

	Risk of developing melioidosis					
	Environmental screening performed by conventional approach		Environmental screening performed by CRISPR-BEEPS		Environmental screening performed by double-qPCR	
	Odds ratio (95% CI)	p-value	Odds ratio (95% CI)	p-value	Odds ratio (95% CI)	p-value
**Participants demographic**						
Age	0.99 (0.97-1.01)	0.219	0.99 (0.97-1.00)	0.229	0.99 (0.97-1.01)	0.220
Sex						
Male (baseline)	1		1		1	
Female	0.75 (0.49-1.14)	0.178	0.75 (0.49-1.14)	0.178	0.75 (0.49-1.14)	0.179
Self-reported ethnicity						
Thai (baseline)	1		1		1	
Others	0.47 (0.02-2.58)	0.472	0.45 (0.02-2.44)	0.440	0.45 (0.02-2.48)	0.449
**Participants underlying health conditions**						
Body-mass index (BMI)	0.88 (0.84-0.92)	1.09×10^{-7}	0.88 (0.84-0.92)	1.17×10^{-7}	0.88 (0.84-0.92)	1.08×10^{-7}
Blood glucose level (HbA1c)	1.23 (1.16-1.31)	7.05×10^{-12}	1.24 (1.16-1.31)	6.50×10^{-12}	1.23 (1.16-1.31)	6.66×10^{-12}
**Participants occupational and exposure risks**						
Occupation						
Homemaker and/or retirees (baseline)	1		1		1	
Agriculture and/or fisheries	4.63 (2.71-8.06)	3.30×10^{-8}	4.58 (2.68-7.97)	4.09×10^{-8}	4.60 (2.69-7.99)	3.90×10^{-8}
Private sector	1.07 (0.56-2.04)	0.838	1.07 (0.56-2.04)	0.828	1.07 (0.56-2.03)	0.835
Merchant	0.63 (0.23-1.55)	0.341	0.64 (0.23-1.57)	0.348	0.63 (0.23-1.56)	0.342
Other	1.33 (0.62-2.80)	0.453	1.35 (0.63-2.84)	0.433	1.34 (0.63-2.82)	0.445
Exposure to flood in the past 6 months						
False (baseline)	1		1		1	
True	1.33 (0.86-2.05)	0.202	1.32 (0.85-2.04)	0.210	1.32 (0.85-2.04)	0.211
B. pseudomallei positivity rates in water samples within 3 km from the household	0.87 (0.19-3.68)	0.859	1.34 (0.74-2.44)	0.334	1.20 (0.66-2.17)	0.554

Supplementary Table 14 Multivariable logistic regression of factors associated with melioidosis based on *B. pseudomallei* detection within 2 km

Multivariable logistic regression was used to identify factors associated with melioidosis risk based on *B. pseudomallei* detection within a 2 km radius of participant households. The analysis included 583 participants, of whom 243 had direct environmental sampling data. Odds ratios (ORs) with 95% confidence intervals (CIs) are reported. The test was two-sided, and reference categories are indicated for categorical variables.

	Risk of developing melioidosis					
	Environmental screening performed by conventional approach		Environmental screening performed by CRISPR-BEEPS		Environmental screening performed by double-qPCR	
	Odds ratio (95% CI)	p-value	Odds ratio (95% CI)	p-value	Odds ratio (95% CI)	p-value
**Participants demographic**						
Age	0.99 (0.97-1.01)	0.1849	0.99 (0.97-1.01)	0.1948	0.99 (0.97-1.01)	0.1808
Sex						
Male (baseline)	1		1		1	
Female	0.64 (0.39-1.02)	0.0642	0.64 (0.39-1.02)	0.0635	0.64 (0.39-1.02)	0.0644
Self-reported ethnicity						
Thai (baseline)	1		1		1	
Others	0.86 (0.03-9.54)	0.9087	0.86 (0.03-9.50)	0.9134	0.85 (0.03-9.27)	0.9035
**Participants underlying health conditions**						
Body-mass index (BMI)	0.86 (0.81-0.90)	1.99×10^{-8}	0.86 (0.81-0.90)	2.06×10^{-8}	0.86 (0.81-0.90)	1.92×10^{-8}
Blood glucose level (HbA1c)	1.24 (1.16-1.33)	8.14×10^{-10}	1.24 (1.16-1.33)	8.53×10^{-10}	1.25 (1.16-1.35)	7.94×10^{-10}
**Participants occupational and exposure risks**						
Occupation						
Homemaker and/or retirees (baseline)	1		1		1	
Agriculture and/or fisheries	4.58 (2.49-8.58)	1.33×10^{-6}	4.57 (2.49-8.56)	1.32×10^{-6}	4.58 (2.49-8.57)	1.31×10^{-6}
Private sector	0.99 (0.48-2.00)	0.9716	0.99 (0.48-2.00)	0.9708	0.98 (0.48-1.99)	0.9630
Merchant	0.87 (0.30-2.23)	0.7756	0.87 (0.30-2.23)	0.7771	0.86 (0.30-2.23)	0.7724
Other	1.25 (0.54-2.84)	0.5915	1.26 (0.54-2.85)	0.5838	1.25 (0.54-2.82)	0.5980
Exposure to flood in the past 6 months						
False (baseline)	1		1		1	
True	1.42 (0.87-2.32)	0.1600	1.42 (0.86-2.32)	0.1630	1.42 (0.87-2.33)	0.1594
B. pseudomallei positivity rates in water samples within 2 km from the household	0.95 (0.21-3.80)	0.9485	1.10 (0.58-2.08)	0.7717	0.96 (0.50-1.83)	0.9066

Supplementary Table 15 Multivariable logistic regression of factors associated with melioidosis based on *B. pseudomallei* detection within 1 km

Multivariable logistic regression was used to identify factors associated with melioidosis risk, defined by the detection of *B. pseudomallei* within a 1 km radius of participant households. The analysis included 443 participants, of whom 243 had direct environmental sampling data. Odds ratios (ORs) with 95% confidence intervals (CIs) are reported. The test was two-sided, and reference categories are indicated for categorical variables.

	**Risk of developing melioidosis**					
	**Environmental screening performed by conventional approach**		**Environmental screening performed by CRISPR-BEEPS**		**Environmental screening performed by double-qPCR**	
	Odds ratio (95% CI)	p-value	Odds ratio (95% CI)	p-value	Odds ratio (95% CI)	p-value
**Participants demographic**						
Age	0.97 (0.95-1.00)	0.039733	0.97 (0.95-1.00)	0.046296	0.97 (0.95-1.00)	0.041873
Sex						
Male (baseline)	1		1		1	
Female	0.57 (0.30-1.03)	0.068267	0.57 (0.30-1.03)	0.067675	0.57 (0.30-1.03)	0.067694
Self-reported ethnicity						
Thai (baseline)	1		1		1	
Others	0.92 (0.04-9.82)	0.948382	1.00 (0.04-11.00)	0.996989	0.99 (0.04-10.8)	0.992862
**Participants underlying health conditions**						
Body-mass index (BMI)	0.85 (0.80-0.90)	7.60×10^{-7}	0.85 (0.80-0.91)	1.05×10^{-6}	0.85 (0.80-0.91)	9.80×10^{-7}
Blood glucose level (HbA1c)	1.25 (1.15-1.35)	3.75×10^{-8}	1.25 (1.15-1.35)	4.02×10^{-8}	1.25 (1.15-1.35)	4.09×10^{-8}
**Participants occupational and exposure risks**						
Occupation						
Homemaker and/or retirees (baseline)	1		1		1	
Agriculture and/or fisheries	4.03 (1.91-8.72)	0.000315	4.04 (1.91-8.75)	0.000314	4.05 (1.91-8.79)	0.000305
Private sector	0.90 (0.38-2.08)	0.809571	0.94 (0.40-2.16)	0.883005	0.93 (0.40-2.15)	0.869598
Merchant	0.90 (0.25-2.79)	0.868505	0.89 (0.25-2.75)	0.849865	0.89 (0.25-2.73)	0.841597
Other	1.48 (0.57-3.75)	0.415676	1.25 (0.59-3.86)	0.375452	1.51 (0.59-3.83)	0.382394
Exposure to flood in the past 6 months						
False (baseline)	1		1		1	
True	1.63 (0.90-2.93)	0.105231	1.66 (0.92-2.99)	0.089466	1.66 (0.92-2.99)	0.089725
B. pseudomallei positivity rates in water samples within 1 km from the household	1.81 (0.47-6.40)	0.366763	1.11 (0.57-2.15)	0.759631	1.00 (0.51-1.93)	0.998077

Supplementary Table 16 Stages of CRISPR-BEEPs development benchmarked by WHO REASSURED guideline

The performance and operational characteristics of the CRISPR-BEEPs assay are summarised and benchmarked against the WHO REASSURED criteria. Current assay performance is compared with anticipated improvements in future development. Parameters assessed include diagnostic target, test format, end-user setting, cost, sensitivity and specificity, workflow complexity, turnaround time, equipment requirements, and storage and distribution conditions.

Test parameters	CRISPR-BEEPs	Further development
Diagnostic target	DNA	DNA
Test format	Lateral flow dipstick	Lateral flow dipstick
End-user	District (technicians)	District (technicians)
**R**eal-time connectivity	Human input to interpret results is still required	Human input to interpret results is still required
**E**ase of specimen collection and processing	Dedicated DNA extraction step is still required	Dedicated DNA extraction step is still required
**A**ffordable (A reaction cost)<br>(Staff cost per day)	~ 15 USD (laboratory scale)<br>~ 20.0 to 28.7 USD per staff/day	<10 USD (pre-industrial scale)<br>~ 20.0 to 28.7 USD staff/day
**S**ensitivity (%)	93.5	93.5 or less to compromise one pot reaction
**S**pecificity (%)	100.0	100.0
**U**ser-friendly	Multiple processing steps are still required	Simplified processing with pre-measured freeze-dried pellet
**R**apid and robust	~3 days total turnaround, as a culture step is required to distinguish viable from non-viable cells	~3 days total turnaround, as a culture step is required to distinguish viable from non-viable cells
**E**quipment-free	Heat block and pipette are still needed	Heat block and pipette are still needed
**D**eliverable	Cold shipment and refrigerator required	Shipment and storage can be performed at room temperature

Supplementary Table 17 Established qPCR test benchmarked against WHO REASSURED criteria

The performance and operational characteristics of established real-time PCR assay are summarised and benchmarked against the WHO REASSURED criteria. Key parameters include diagnostic target, test format, end-user setting, cost, analytical performance, workflow requirements, and infrastructure needs. Limitations are highlighted to reflect constraints in decentralized or resource-limited settings.

Test parameters	Established PCR (qPCR)	Limitations
Diagnostic target	DNA	DNA only; cannot distinguish viable vs non-viable organisms
Test format	Real-time PCR (fluorescence-based)	Fixed laboratory format
End-user	Central/regional laboratory staff	Difficult for district-level deployment due to machine availability
**R**eal-time connectivity	Automated digital readout	Requires specialised software and data systems
**E**ase of specimen collection and processing	Requires DNA extraction and purification	Dedicated DNA extraction and clean lab environment needed
**A**ffordable (A reaction cost)<br>(Staff cost per day)	~ 5 -10 USD per reaction (reagents only)<br>~ 20.0 to 28.7 USD per staff/day	High capital cost for instrument; maintenance and calibration required.<br>Also highly trained personal required
**S**ensitivity (%)	Very high (often >95%)[31]	Sensitivity may overestimate risk due to detection of residual DNA
**S**pecificity (%)	Very high (typically 98-100%)[32]	Dependent on primer/probe design and contamination control.
**U**ser-friendly	Low-moderate	Multiple pipetting steps; risk of contamination
**R**apid and robust	Hours once DNA is extracted	Not robust to field conditions (power, dust, humidity)

**E**quipment-free	No	Requires thermocycler, centrifuge, cold chain, and uninterrupted power.
**D**eliverable	Cold shipment and refrigerator required	Cold shipment and refrigerator required

SUPPLEMENTARY REFERENCES

1 Limmathurotsakul D, Dance DAB, Wuthiekanun V, *et al.* Systematic Review and Consensus Guidelines for Environmental Sampling of Burkholderia pseudomallei. *PLoS Negl Trop Dis* 2013; **7**: e2105.

2 Wongpalee SP, Thananchai H, Chewapreecha C, *et al.* Highly specific and sensitive detection of Burkholderia pseudomallei genomic DNA by CRISPR-Cas12a. *PLoS Negl Trop Dis* 2022; **16**: e0010659.

3 Pakdeerat S, Boonklang P, Angchagun K, *et al.* Benchmarking CRISPR-BP34 for point-of-care melioidosis detection in low-income and middle-income countries: a molecular diagnostics study. *The Lancet Microbe* 2024; **5**: e379–89.

4 Merianos A, Patel M, Lane JM, *et al.* The 1990-1991 outbreak of melioidosis in the Northern Territory of Australia: epidemiology and environmental studies. *Southeast Asian J Trop Med Public Health* 1993; **24**: 425–35.

5 Parry CM, Wuthiekanun V, Hoa NTT, *et al.* Melioidosis in Southern Vietnam: Clinical Surveillance and Environmental Sampling. *Clinical Infectious Diseases* 1999; **29**: 1323–6.

6 Vuddhakul V, Tharavichitkul P, Na-Ngam N, *et al.* Epidemiology of Burkholderia pseudomallei in Thailand. *Am J Trop Med Hyg* 1999; **60**: 458–61.

7 Inglis TJJ, Foster NF, Gal D, *et al.* Preliminary report on the northern Australian melioidosis environmental surveillance project. *Epidemiol Infect* 2004; **132**: 813–20.

8 Warner JM, Pelowa DB, Gal D, *et al.* The epidemiology of melioidosis in the Balimo region of Papua New Guinea. *Epidemiol Infect* 2008; **136**: 965–71.

9 Rolim DB, Rocha MFG, Brilhante RSN, *et al.* Environmental isolates of Burkholderia pseudomallei in Ceará State, northeastern Brazil. *Appl Environ Microbiol* 2009; **75**: 1215–8.

10 Limmathurotsakul D, Wuthiekanun V, Chantratita N, *et al.* Burkholderia pseudomallei Is Spatially Distributed in Soil in Northeast Thailand. *PLoS Negl Trop Dis* 2010; **4**: e694.

11 Sapian M, Khair MT, How SH, *et al.* Outbreak of melioidosis and leptospirosis co-infection following a rescue operation. *Med J Malaysia* 2012; **67**: 293–7.

12 Wiersinga WJ, Birnie E, Weehuizen TAF, *et al.* Clinical, environmental, and serologic surveillance studies of melioidosis in Gabon, 2012-2013. *Emerg Infect Dis* 2015; **21**: 40–7.

13 Ashdown LR. An improved screening technique for isolation of Pseudomonas pseudomallei from clinical specimens. *Pathology* 1979; **11**: 293–7.

14 Zimmermann RE, Ribolzi O, Pierret A, *et al.* Rivers as carriers and potential sentinels for Burkholderia pseudomallei in Laos. *Sci Rep* 2018; **8**: 8674.

15 Shaw T, Assig K, Tellapragada C, *et al.* Environmental Factors Associated With Soil Prevalence of the Melioidosis Pathogen Burkholderia pseudomallei: A Longitudinal Seasonal Study From South West India. *Front Microbiol* 2022; **13**: 902996.

16 Trung TT, Hetzer A, Göhler A, *et al.* Highly sensitive direct detection and quantification of Burkholderia pseudomallei bacteria in environmental soil samples by using real-time PCR. *Appl Environ Microbiol* 2011; **77**: 6486–94.

17 Rachlin A, Luangraj M, Kaestli M, *et al.* Using Land Runoff to Survey the Distribution and Genetic Diversity of Burkholderia pseudomallei in Vientiane, Laos. *Appl Environ Microbiol* 2021; **87**: e02112-20, AEM.02112-20.

18 Seng R, Saiprom N, Phunpang R, *et al.* Prevalence and genetic diversity of Burkholderia pseudomallei isolates in the environment near a patient's residence in Northeast Thailand. *PLoS Negl Trop Dis* 2019; **13**: e0007348.

19 Nithimongkolchai N, Hinwan Y, Kaewseekhao B, *et al.* MALDI-TOF MS analysis of Burkholderia pseudomallei and closely related species isolated from soils and water in Khon Kaen, Thailand. *Infect Genet Evol* 2023; **116**: 105532.

20 Birnie E, Van 'T Hof S, Bijnsdorp A, *et al.* Identification of Burkholderia thailandensis with novel genotypes in the soil of central Sierra Leone. *PLoS Negl Trop Dis* 2019; **13**: e0007402.

21 Knappik M, Dance DAB, Rattanavong S, *et al.* Evaluation of Molecular Methods To Improve the Detection of Burkholderia pseudomallei in Soil and Water Samples from Laos. *Appl Environ Microbiol* 2015; **81**: 3722–7.

22 Baker AL, Warner JM. Burkholderia pseudomallei is frequently detected in groundwater that discharges to major watercourses in northern Australia. *Folia Microbiol* 2016; **61**: 301–5.

23 Angchagun K, Boonklang P, Chomkatekaew C, *et al.* BurkHostGEN: a study protocol for evaluating variations in the Burkholderia pseudomallei and host genomes associated with melioidosis infection. *Wellcome Open Res* 2023; **8**: 347.

24 Limmathurotsakul D, Wongsuvan G, Aanensen D, *et al.* Melioidosis caused by Burkholderia pseudomallei in drinking water, Thailand, 2012. *Emerg Infect Dis* 2014; **20**: 265–8.

25 Hantrakun V, Kongyu S, Klaytong P, *et al.* Clinical Epidemiology of 7,126 Melioidosis Patients in Thailand and the Implications for a National Notifiable Diseases Surveillance System. *Open Forum Infectious Diseases* 2019; : ofz498.

26 Kanoksil M, Jatapai A, Peacock SJ, Limmathurotsakul D. Epidemiology, microbiology and mortality associated with community-acquired bacteremia in northeast Thailand: a multicenter surveillance study. *PLoS One* 2013; **8**: e54714.

27 Southeast Asia Infectious Disease Clinical Research Network. Causes and outcomes of sepsis in southeast Asia: a multinational multicentre cross-sectional study. *Lancet Glob Health* 2017; **5**: e157–67.

28 Nybo K. qPCR Efficiency Calculations. *BioTechniques* 2011; **51**: 401–2.

29 Novak RT, Glass MB, Gee JE, *et al.* Development and evaluation of a real-time PCR assay targeting the type III secretion system of Burkholderia pseudomallei. *J Clin Microbiol* 2006; **44**: 85–90.

30 Lowe W, March JK, Bunnell AJ, O'Neill KL, Robison RA. PCR-based Methodologies Used to Detect and Differentiate the Burkholderia pseudomallei complex: B. pseudomallei, B. mallei, and B. thailandensis. *Curr Issues Mol Biol* 2014; **16**: 23–54.

31 Binny RN, Priest P, French NP, *et al.* Sensitivity of Reverse Transcription Polymerase Chain Reaction Tests for Severe Acute Respiratory Syndrome Coronavirus 2 Through Time. *J Infect Dis* 2022; **227**: 9–17.

32 Skittrall JP, Wilson M, Smielewska AA, *et al.* Specificity and positive predictive value of SARS-CoV-2 nucleic acid amplification testing in a low-prevalence setting. *Clin Microbiol Infect* 2021; **27**: 469.e9-469.e15.
