# Supplementary material for: CRISPR-based environmental detection of Burkholderia pseudomallei identifies sanitation gaps and melioidosis risk in northeast Thailand
Source: Nat Commun. 2026 May 15;17:6460. doi: 10.1038/s41467-026-73286-8 (PMC13376406; doi:10.1038/s41467-026-73286-8)
Supplement: Supplementary file 2 — Reporting Summary [file 41467_2026_73286_MOESM2_ESM.pdf]

Corresponding author(s): Claire Chewapreecha

Last updated by author(s): Mar 15, 2026

# Reporting Summary

Nature Portfolio wishes to improve the reproducibility of the work that we publish. This form provides structure for consistency and transparency in reporting. For further information on Nature Portfolio policies, see our Editorial Policies and the Editorial Policy Checklist.

## Statistics

For all statistical analyses, confirm that the following items are present in the figure legend, table legend, main text, or Methods section.

| n/a | Confirmed | |
|---|---|---|
| ☐ | ☒ | The exact sample size (*n*) for each experimental group/condition, given as a discrete number and unit of measurement |
| ☐ | ☒ | A statement on whether measurements were taken from distinct samples or whether the same sample was measured repeatedly |
| ☐ | ☒ | The statistical test(s) used AND whether they are one- or two-sided<br>*Only common tests should be described solely by name; describe more complex techniques in the Methods section.* |
| ☐ | ☒ | A description of all covariates tested |
| ☐ | ☒ | A description of any assumptions or corrections, such as tests of normality and adjustment for multiple comparisons |
| ☐ | ☒ | A full description of the statistical parameters including central tendency (e.g. means) or other basic estimates (e.g. regression coefficient) AND variation (e.g. standard deviation) or associated estimates of uncertainty (e.g. confidence intervals) |
| ☐ | ☒ | For null hypothesis testing, the test statistic (e.g. *F*, *t*, *r*) with confidence intervals, effect sizes, degrees of freedom and *P* value noted<br>*Give P values as exact values whenever suitable.* |
| ☒ | ☐ | For Bayesian analysis, information on the choice of priors and Markov chain Monte Carlo settings |
| ☒ | ☐ | For hierarchical and complex designs, identification of the appropriate level for tests and full reporting of outcomes |
| ☒ | ☐ | Estimates of effect sizes (e.g. Cohen's *d*, Pearson's *r*), indicating how they were calculated |

*Our web collection on statistics for biologists contains articles on many of the points above.*

## Software and code

Policy information about availability of computer code

| | |
|---|---|
| Data collection | Genomic sequence data used for in silico confirmation of target DNA sequences were downloaded from publicly available repositories referenced in PMID: 33169036, 28112723, 31799430, 34662416, and 38972886. Epidemiological data for the observational case–control cohort were collected from clinical records and patient interviews. Data were entered into MACRO EDC version 4, a clinical data management system, and de-identified prior to downstream analysis. |
| Data analysis | Genomic analyses to assess the presence or absence of target DNA sequences were performed using BLASTn v2.16.0 for metagenome-assembled genomes and BLAT v36 for Burkholderia pseudomallei assembled genomes. Statistical analyses were conducted in R v4.5.2, with the geosphere package v.1.6-5 used to estimate distances between household locations and sampling sites based on GPS coordinates. All software used in this study is open access. |

For manuscripts utilizing custom algorithms or software that are central to the research but not yet described in published literature, software must be made available to editors and reviewers. We strongly encourage code deposition in a community repository (e.g. GitHub). See the Nature Portfolio guidelines for submitting code & software for further information.

## Data

Policy information about availability of data

All manuscripts must include a data availability statement. This statement should provide the following information, where applicable:

- Accession codes, unique identifiers, or web links for publicly available datasets
- A description of any restrictions on data availability
- For clinical datasets or third party data, please ensure that the statement adheres to our policy

De-identified participant data generated in this study are available under restricted access due to ethical and consent constraints, limiting use to purposes consistent with the approved study protocol. Requests should be submitted to the corresponding author (claire@tropmedres.ac) with a brief proposal outlining the intended use. Requests are reviewed for compliance with ethical approvals and consent conditions, with responses provided within 2-4 weeks. Approved access is subject to a data use agreement. Processed data generated in this study are provided in the Supplementary Information and Source Data. Environmental metagenomic and B. pseudomallei sequence data used in this study are available in the repositories referenced in 39,44–46,48. Bulk downloads of the metagenome-assembled genome dataset are available from the Joint Genome Institute GEMs database ( https://genome.jgi.doe.gov/GEMs). Accession codes for B. pseudomallei genome assemblies used in this study are available via Figshare https://doi.org/10.6084/m9.figshare.31742224.

## Research involving human participants, their data, or biological material

Policy information about studies with human participants or human data. See also policy information about sex, gender (identity/presentation), and sexual orientation and race, ethnicity and racism.

| | |
|---|---|
| Reporting on sex and gender | Sex was included as a biological variable in multivariable regression analyses examining factors associated with melioidosis susceptibility. Sex was determined based on clinical records at enrolment. All participants provided informed consent for the use and sharing of de-identified clinical and epidemiological data for research purpose consistent with the scope of their consent. The distribution of both sexes is reported in Supplementary Table 4. |
| Reporting on race, ethnicity, or other socially relevant groupings | Self-reported ethnicity was included as a variable in multivariable regression analyses of melioidosis susceptibility. The study population was recruited from northeast Thailand, a region bordering Laos and Cambodia, and reported ethnicities included Thai, Lao, and Cambodian. Ethnicity data were collected through participant interview and are summarised in Supplementary Table 4. These variables were included to account for population structure rather than to imply biological causation. |
| Population characteristics | Covariate-relevant population characteristics included age, sex, ethnicity and body mass index. Established risk factors for melioidosis were also recorded, including diabetes status assessed using HbA1c measurements to reduce misclassification due to undiagnosed diabetes, occupation, and exposure to flooding. These variables were collected at enrolment and included as covariates in the epidemiological analyses. |
| Recruitment | Participants were recruited at Sunpasitthiprasong Hospital, a regional referred hospital in Ubon Ratchathani, Thailand, which serves the local population and surrounding provinces and has extensive experience in diagnosing and managing melioidosis due to high regional incidence. Patients with melioidosis and other infectious diseases were recruited from the hospital's infectious disease department. Healthy control participants without diabetes mellitus were recruited from the hospital blood donor clinic, while healthy control participants with diabetes were recruited from diabetes outpatient clinic. Recruitment efforts aimed to achieve representation across age groups and both sexes. |
| Ethics oversight | The study protocol was reviewed and approved by Sunpasitthiprasong Hospital Ethical Review Board (015/62C) and the Oxford Tropical Research Ethics Committee (OxTREC, 25-19). Written informed consent was obtained from all participants prior to enrolment in the study. |

Note that full information on the approval of the study protocol must also be provided in the manuscript.

# Field-specific reporting

Please select the one below that is the best fit for your research. If you are not sure, read the appropriate sections before making your selection.

☐ Life sciences ☐ Behavioural & social sciences ☒ Ecological, evolutionary & environmental sciences

For a reference copy of the document with all sections, see nature.com/documents/nr-reporting-summary-flat.pdf

# Ecological, evolutionary & environmental sciences study design

All studies must disclose on these points even when the disclosure is negative.

| | |
|---|---|
| Study description | This study comprises two components: (i) optimisation and validation of a CRISPR-BEEPs assay for detection of Burkholderia pseudomallei in water, benchmarked against a standard double-qPCR assays; and (ii) an environmental surveillance study assessing the association between B. pseudomallei positivity in household-associated water source and melioidosis incidence in cases and controls from the same endemic region, adjusting for relevant comorbidities. |
| Research sample | Environmental water samples were analysed to detect Burkholderia pseudomallei, a Gram-negative environmental bacterium and the causative agent of melioidosis. Samples were collected from natural and household-associated water sources, including groundwater, surface water and piped water, representing environmental exposures encountered by residents in melioidosis- |

| | |
|---|---|
| | endemic communities in northeast Thailand. Water was selected as the environmental matrix because it has lower matrix complexity, reduced spatial heterogeneity and fewer assay-inhibitory substances compared with soil, enabling more reliable molecular detection of B. pseudomallei. No experimental manipulation of organisms was performed; the study focused on detecting naturally occurring environmental bacteria in collected samples. |
| Sampling strategy | Of the 356 water samples collected 21 were excluded due to fungal overgrowth, resulting in 335 interpretable samples for analysis.<br><br>For assay validation, sample size was calculated using $n = z^2 \times p \times (1 - p) / d^2$, where z = 1.96 (95% confidence interval), p = 0.5, and d = 0.1. A minimum of 96 positive and 96 negative environmental samples were required to validate the CRISPR-BEEPs assay.<br><br>For environmental surveillance, sample size were based on previously reported exposure prevalences of 7% in cases and 3% in controls (PMID: 23437412), obtained using culture-based methods with an estimated sensitivity of approximately 20%. These prevalences correspond to estimated true exposure prevalences of 35% in cases and 15% in controls. Under these assumptions, 70 cases and 70 controls provided 80% power to detect a difference at a two-sided significance level of $\alpha = 0.05$.<br><br>The number of samples and participants included in this study exceeded these minimum requirements. |
| Data collection | Water samples were collected from household water sources by trained field staff. During periods of restricted access related to SARS-CoV-2 outbreaks, samples were obtained from nearby communal water reservoirs. Water sources included piped water, boreholes, ponds, lakes, canals and rivers (Fig. 2d; Supplementary Fig. 1). Samples were collected in sterile containers and transported to the laboratory within three hours of collection for immediate processing. Geographic coordinates of sampling locations were recorded by the field team at the time of collection to enable spatial analyses. Metadata including water source type and date of collection were entered into the MACRO EDC database by study staff. To protect household confidentiality, precise coordinates were not retained in the final shared dataset. |
| Timing and spatial scale | Sampling was conducted between November 2020 and November 2021, spanning both wet and dry seasons. The study period overlapped with SARS-CoV-2 outbreaks, which limited repeat interval sampling. Sampling covered approximately 15,118 $km^2$ in northeast Thailand and was restricted to locations within three hours' driving distance of the laboratory. |
| Data exclusions | Samples with fungal overgrowth on plate culture were excluded |
| Reproducibility | Environmental surveillance employed a double-qPCR assay targeting two independent loci and a CRISPR-BEEPs assay targeting a third locus. All assays were performed in duplicate to minimise stochastic detection and confirm reproducibility of results. For epidemiological analyses, sensitivity analyses were conducted by varying temporal categories and geographical proximity thresholds to assess the robustness of associations between environmental B. pseudomallei detection, seasonality and melioidosis incidence. All attempts to reproduce the experimental and analytical findings were successful. |
| Randomization | Cases and controls, and their corresponding water samples, were not randomised, as case-control status constituted the dependent and independent variables of interest in the analysis. |
| Blinding | Personnel performing molecular and culture-based detection of B. pseudomallei in water samples were blinded to the results of the alternative detection methods during laboratory processing. Blinding was not relevant for environmental sampling or epidemiological analyses, which were based on predefined sampling locations and objective molecular assays and recorded metadata without investigator-assigned group allocation. |

Did the study involve field work? ☒ Yes ☐ No

## Field work, collection and transport

| | |
|---|---|
| Field conditions | The study was conducted in northeast Thailand, a melioidosis-endemic region with a tropical monsoon climate characterised by distinct wet and dry seasons and mean annual temperatures typically ranging from approximately 23–37 °C. The area lies within the lower Mekong River basin and consists largely of low-lying floodplains with seasonal flooding and abundant surface water. Land use is predominantly agricultural, particularly irrigated rice cultivation, and communities rely on multiple water sources including piped water, boreholes and surface water bodies. The region contains an extensive network of permanent and seasonally inundated water systems that vary with monsoon rainfall and influence environmental exposure to Burkholderia pseudomallei. |
| Location | The study area spans 14.99-15.9N latitude and 103.1-104.85E longitude, with elevations of approximately 100-200 m above sea level that decrease eastward toward the Mekong River floodplain. |
| Access & import/export | All participants provided informed consent for access to their properties for water sample collection. The study protocol was approved by the Sunpasitthiprasong Hospital Ethical Review Board (015/62C) and the Oxford Tropical Research Ethics Committee (OxTREC, 25-19). |
| Disturbance | Water samples were collected by trained staff, with collection times arranged to minimise disruption to participants' daily routines and conducted in a culturally sensitive manner that respected participants and their communities. |

# Reporting for specific materials, systems and methods

We require information from authors about some types of materials, experimental systems and methods used in many studies. Here, indicate whether each material, system or method listed is relevant to your study. If you are not sure if a list item applies to your research, read the appropriate section before selecting a response.

## Materials & experimental systems

| n/a | Involved in the study |
|---|---|
| x | Antibodies |
| x | Eukaryotic cell lines |
| x | Palaeontology and archaeology |
| x | Animals and other organisms |
|  | x Clinical data |
| x | Dual use research of concern |
| x | Plants |

## Methods

| n/a | Involved in the study |
|---|---|
| x | ChIP-seq |
| x | Flow cytometry |
| x | MRI-based neuroimaging |

# Clinical data

Policy information about clinical studies

All manuscripts should comply with the ICMJE guidelines for publication of clinical research and a completed CONSORT checklist must be included with all submissions.

| | |
|---|---|
| Clinical trial registration | This observational study is registered with the Thai Clinical Trial Registry (TCTR20190322003) |
| Study protocol | The protocol is summarised in Supplementary Methods with full protocol is available in PMID: 37928212 |
| Data collection | Cases and controls were enrolled between October 2019 and January 2023. Both groups resided in melioidosis-endemic areas of northeast Thailand. |
| Outcomes | The primary outcome was melioidosis status. Cases were defined by culture-confirmed melioidosis from clinical specimens. Controls were defined as individuals with no history of melioidosis despite long-term residence in the endemic area, confirmed by absence of clinical records and by personal interview. |

# Plants

| | |
|---|---|
| Seed stocks | n/a |
| Novel plant genotypes | n/a |
| Authentication | n/a |
