# Supplementary figures and images for: CRISPR-based environmental detection of Burkholderia pseudomallei identifies sanitation gaps and melioidosis risk in northeast Thailand
Source: Nat Commun. 2026 May 15;17:6460. doi: 10.1038/s41467-026-73286-8 (PMC13376406; doi:10.1038/s41467-026-73286-8)

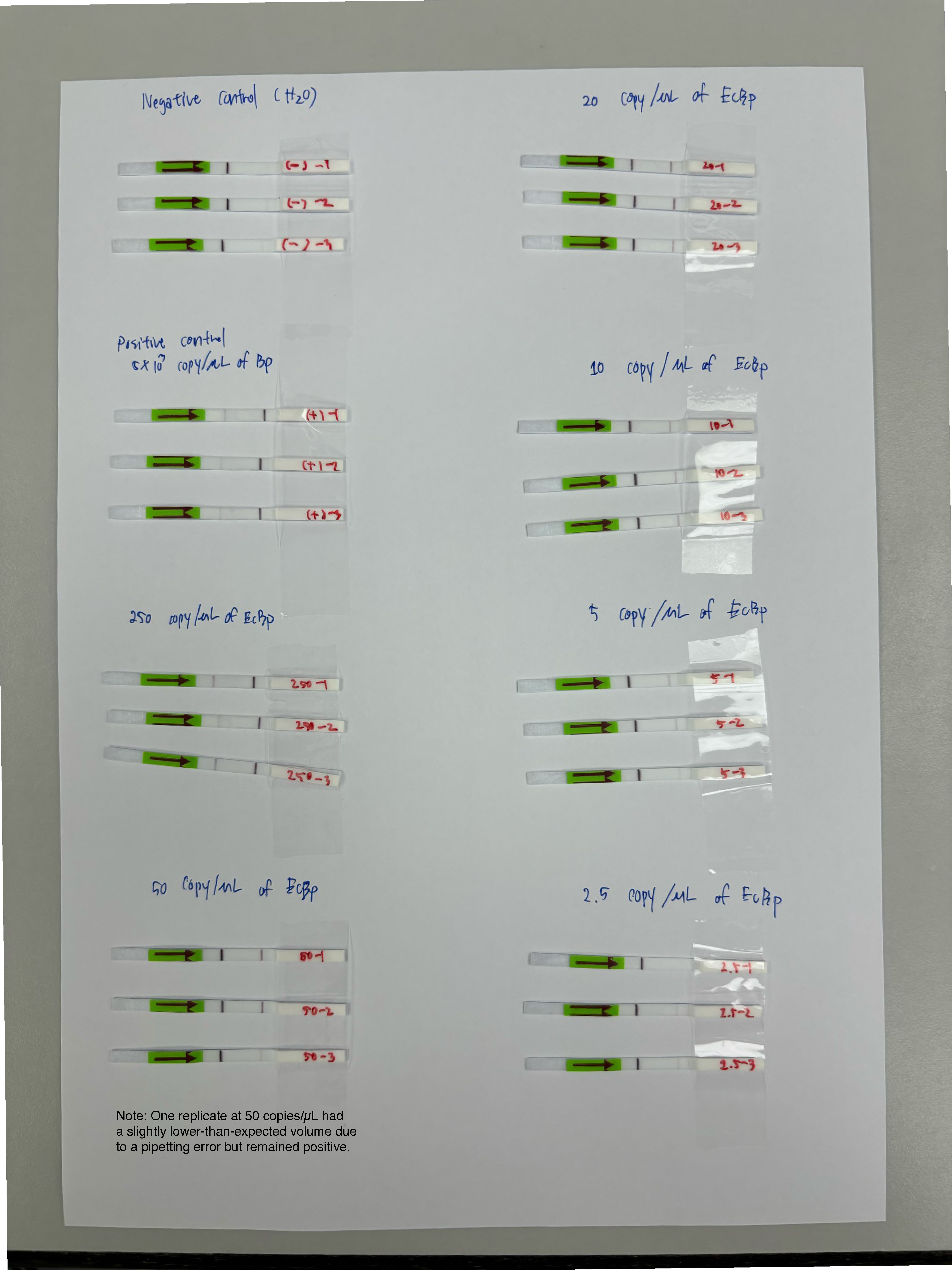

Supplement: Supplementary file 4 — Source Data [file 41467_2026_73286_MOESM4_ESM.zip › R3.CRISPR-BEEPs.Source.Data.files/R3.CRISPR-BEEPs.Source.Data.uncropped.sup.Figure2c.jpg]

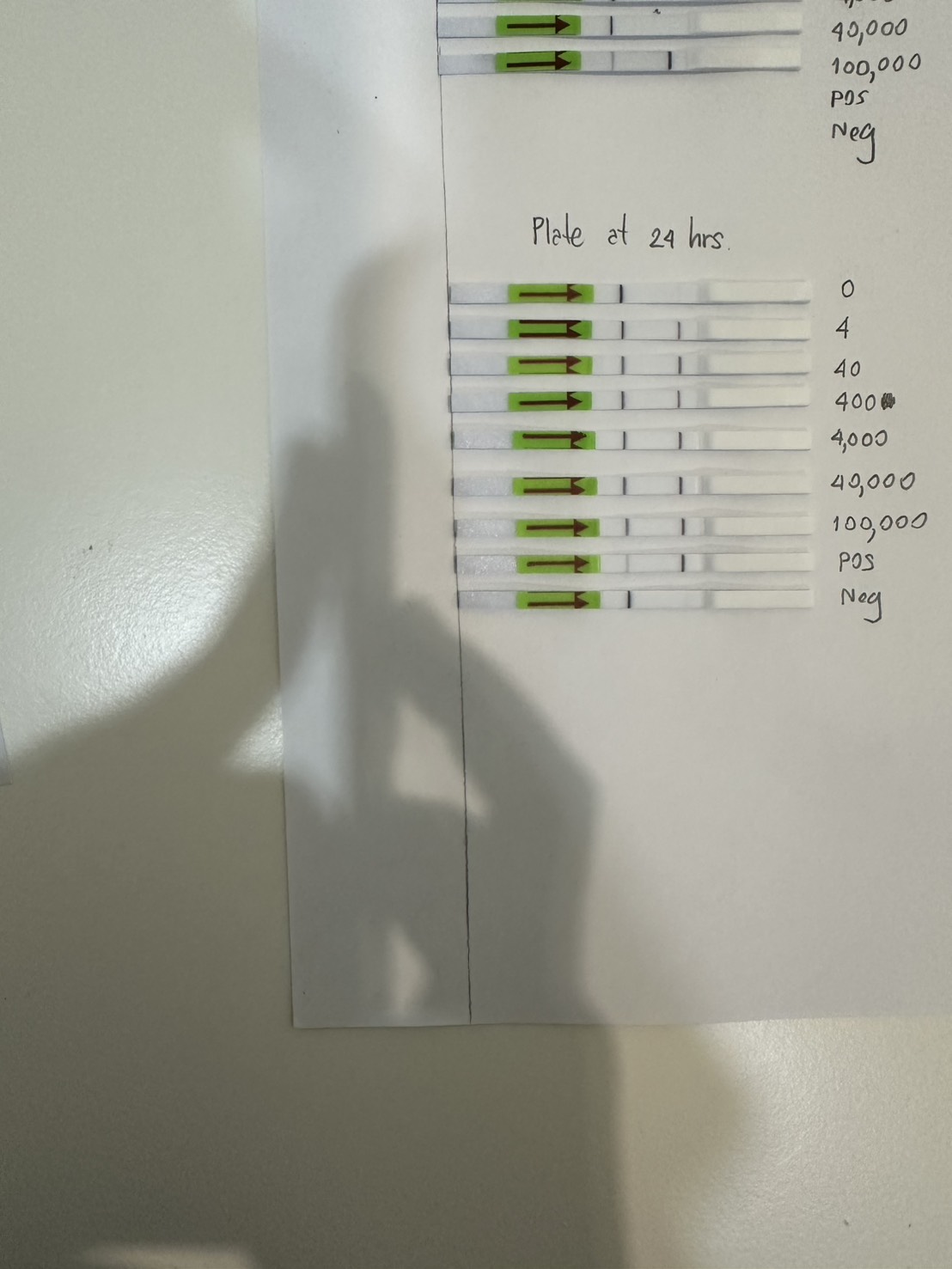

Supplement: Supplementary file 4 — Source Data [file 41467_2026_73286_MOESM4_ESM.zip › R3.CRISPR-BEEPs.Source.Data.files/R3.CRISPR-BEEPs.Source.Data.uncropped.sup.Figure3b.jpg]

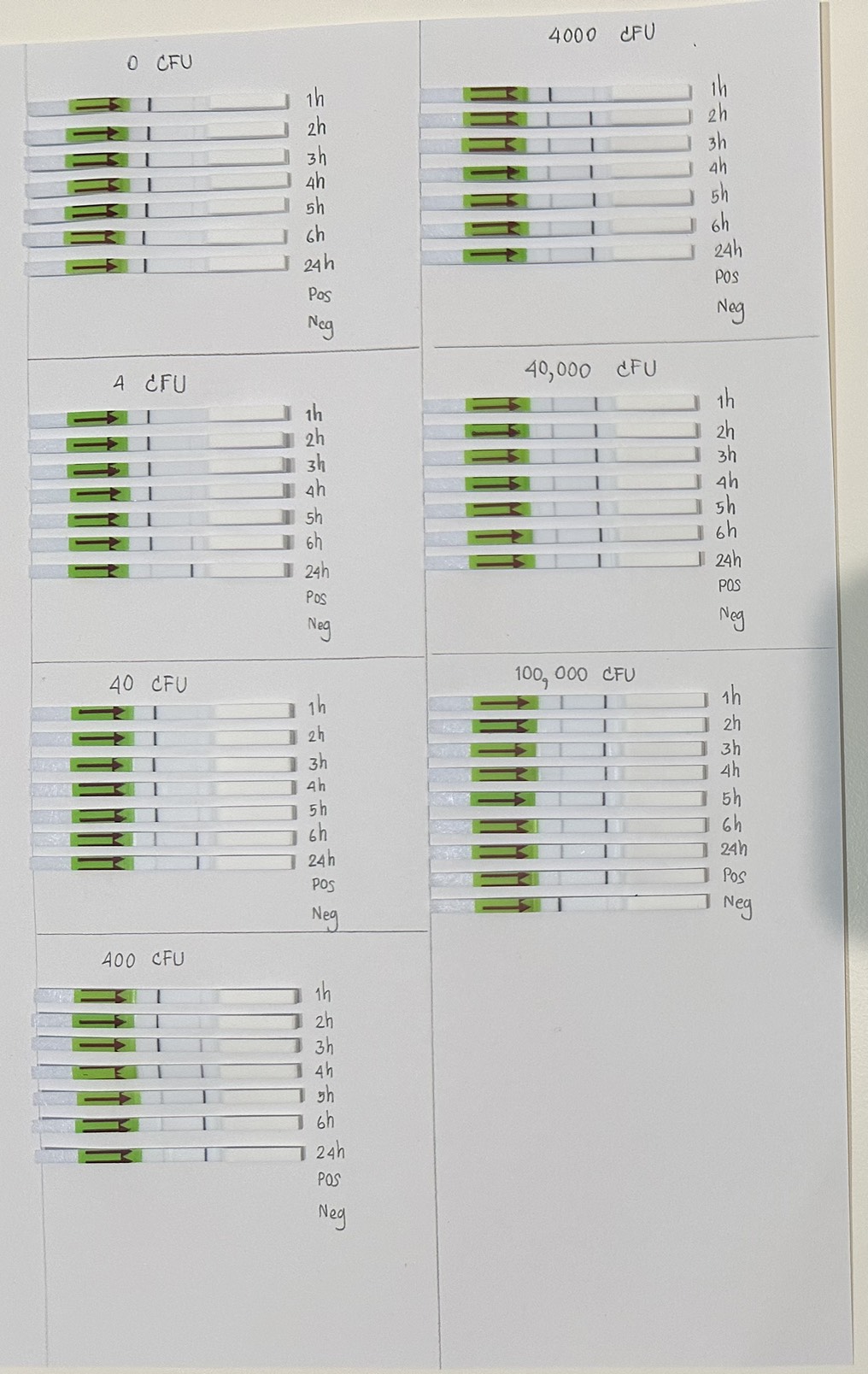

Supplement: Supplementary file 4 — Source Data [file 41467_2026_73286_MOESM4_ESM.zip › R3.CRISPR-BEEPs.Source.Data.files/R3.CRISPR-BEEPs.Source.Data.uncropped.sup.Figure3a.jpg]

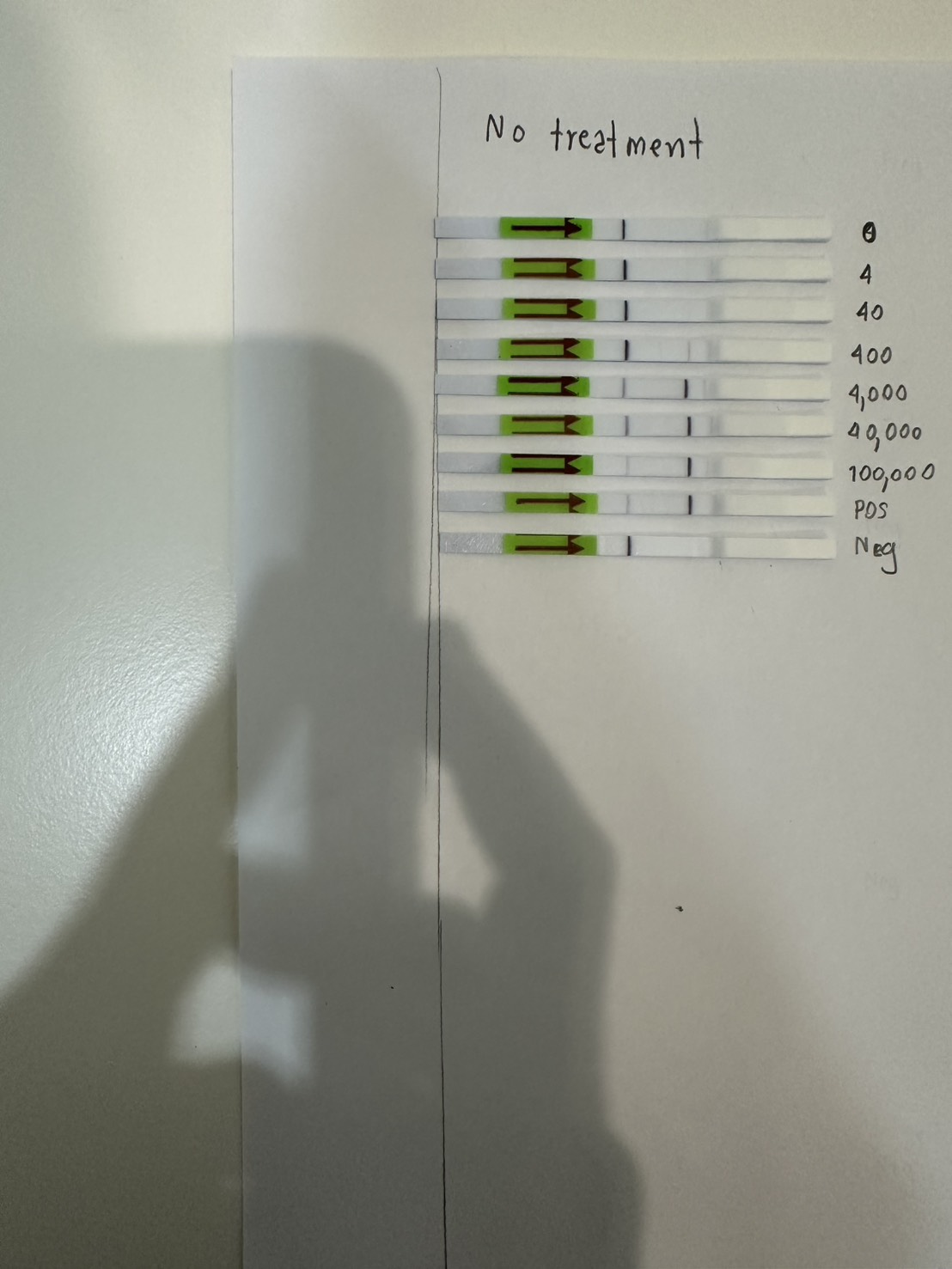

Supplement: Supplementary file 4 — Source Data [file 41467_2026_73286_MOESM4_ESM.zip › R3.CRISPR-BEEPs.Source.Data.files/R3.CRISPR-BEEPs.Source.Data.uncropped.main.Figure3a.no.treatment.jpg]

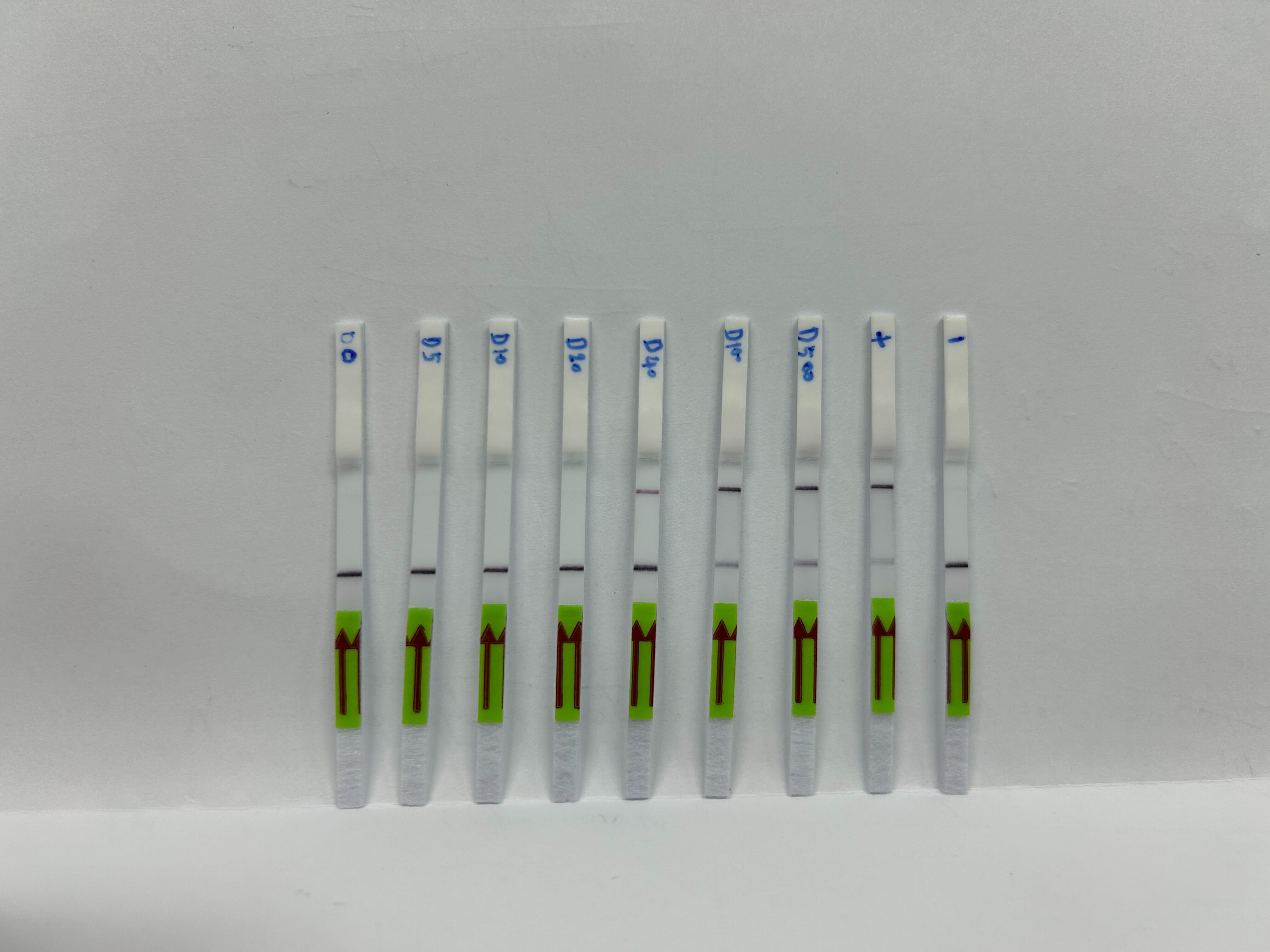

Supplement: Supplementary file 4 — Source Data [file 41467_2026_73286_MOESM4_ESM.zip › R3.CRISPR-BEEPs.Source.Data.files/R3.CRISPR-BEEPs.Source.Data.uncropped.main.Figure2b.jpg]

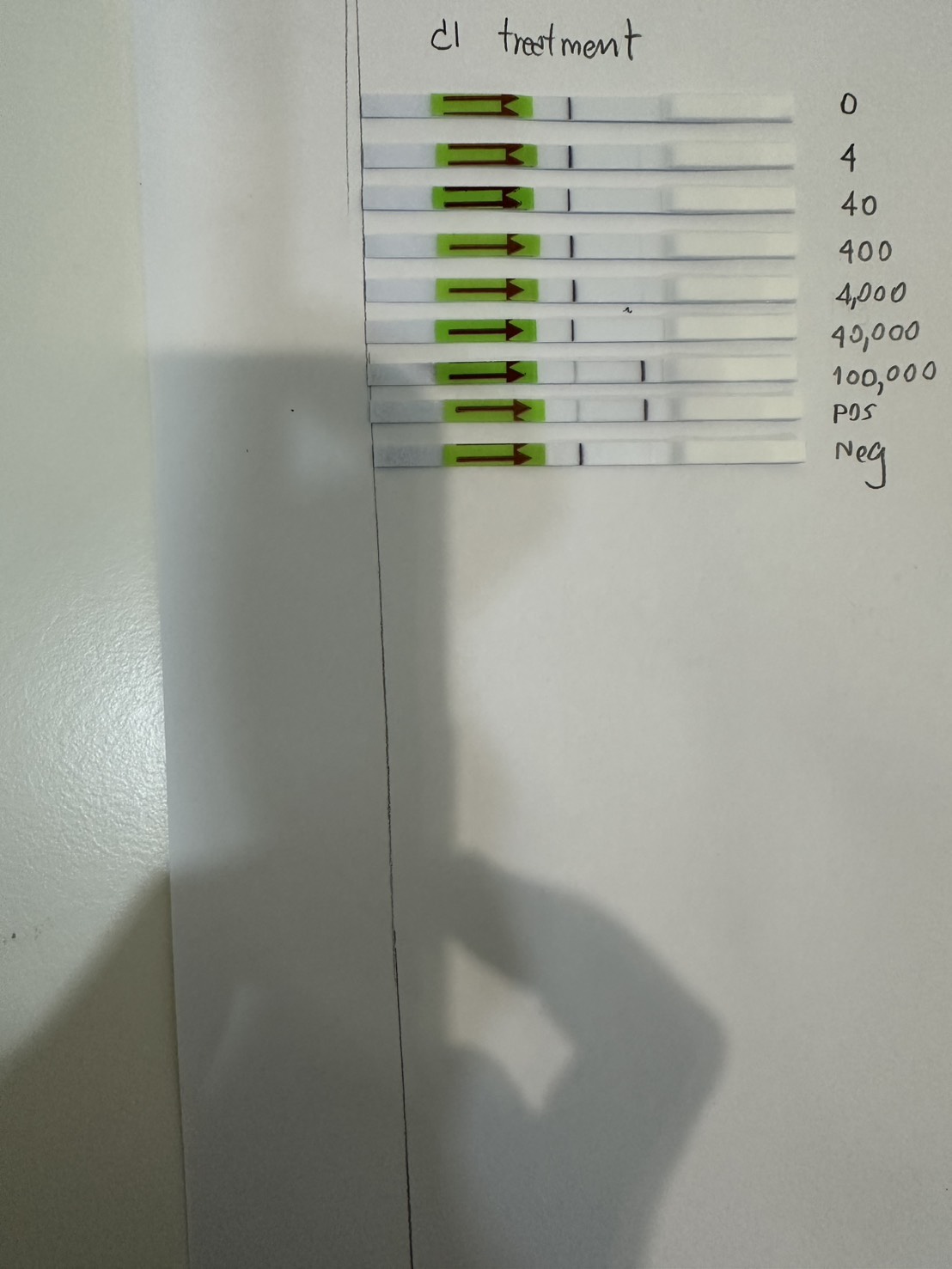

Supplement: Supplementary file 4 — Source Data [file 41467_2026_73286_MOESM4_ESM.zip › R3.CRISPR-BEEPs.Source.Data.files/R3.CRISPR-BEEPs.Source.Data.uncropped.main.Figure3a.Cl.treatment.jpg]
