# Supplementary material for: CRISPR-based environmental detection of Burkholderia pseudomallei identifies sanitation gaps and melioidosis risk in northeast Thailand
Source: Nat Commun. 2026 May 15;17:6460. doi: 10.1038/s41467-026-73286-8 (PMC13376406; doi:10.1038/s41467-026-73286-8)
Supplement: Supplementary file 3 — Transparent Peer Review file [file 41467_2026_73286_MOESM3_ESM.pdf]

nature portfolio

Peer Review File

CRISPR-based environmental detection of Burkholderia pseudomallei identifies sanitation gaps and melioidosis risk in northeast Thailand

Corresponding Author: Dr Claire Chewapreecha

This file contains all reviewer reports in order by version, followed by all author rebuttals in order by version.

Attachments originally included by the reviewers as part of their assessment can be found at the end of this file.

Version 0:

Reviewer comments:

Reviewer #1

(Remarks to the Author)
Reviewer Comments:

There are several studies reporting on melioidosis outbreaks with water as a source of infection. Accessible and sensitive methods for determining B. pseudomallei from environmental samples, including water are essential to eliminate potential infection sources and to raise awareness in endemic regions.

This well-written report by Pakdeerat addresses this topic and is of significance to the field. It demonstrates that the CRISPR-BEEPs method developed can detect B. pseudomallei colonies grown on a culture medium with significantly higher sensitivity than traditional visual inspection of colonies. Using this new methodology, the authors conducted a thorough and comprehensive study, proving that melioidosis cases occur significantly more frequently in households where B. pseudomallei has been detected in nearby water samples. This study will not only inform health policies to strengthen water sanitation but also provides a novel method to detect B. pseudomallei in water samples with improved sensitivity and specificity.

Consideration of the following points may help to further refine the evaluation of the CRISPR-based culture identification method in comparison to other methodological approaches.

Specific comments

1. The CRISPR-BEEPs method is primarily a culture method in which, after filtration of water samples, the filters are inoculated onto Ashdown medium and the growth of B. pseudomallei is detected more quickly and sensitively than by visual inspection of the culture plates. Compared to direct molecular detection, culture remains the time-determining step here. This limitation should be addressed, especially in outbreak situations.

2. It would be interesting to validate the performance of their CRISPR method in comparison to PCR for the direct detection of B pseudomallei DNA from water samples. Various e. g. mechanical methods could be used to recover B. pseudomallei from the filter after filtration of the water samples and test it directly without incubation. One would expect the detection limit of the CRISPR and PCR methods to be reached under such conditions. This would guarantee more timely results and initiation of countermeasures and may also circumvent fungal overgrowth observed in 21 of the tested samples. This potential advantage of direct testing should be discussed.

3. In line 369 to 374 the authors state that the culture step provides a more biologically relevant detection threshold, as it detects viable organisms and not just DNA. Can the authors provide data to support the assumption that the detection of viable cells is more disease-relevant compared to a direct semi-quantitative determination of B. pseudomallei DNA?

- Do the authors have data showing that a positive CRISPR reaction of plates that were negative by conventional inspection

is indeed due to proliferated cells? Comparisons of plate sweeps on day 0 and at later time points after incubation would be important here to show that positive signals were not due to B pseudomallei DNA that was simply washed off the filter, but are the results of bacterial growth.

- Along the same line, did the authors attempt to isolate strains from CRISPR and/or PCR-positive but inspection-negative plate sweeps through additional plating of diluted sweeps?

- How long were the filters incubated on Ashdown agar before colonies were harvested by sweeping the plates?

4. In Line 205-208 the authors state that most environmental water samples are likely to contain B. pseudomallei at a concentration below the direct detection limits of current molecular assays and therefore suggest pre-enrichment by proliferation on agar. In this context the filtration step performed should also be mentioned, as it represents a key enrichment step and could enable culture-independent detection using the CRISPR method. The detection limit of their CRISPR method should be discussed in the context of PCR-based methods, including references.

5. The wording in lines 241 to 242, ‘In contrast to the conventional culture-based method,’ might be changed to ‘in contrast to the conventional culture identification method,’ as it is still a culture-based approach.

6. The authors should consider discussing existing studies that have detected B. pseudomallei, from water samples using PCR of liquid enrichment. Did the authors also used the CRISPR technology with liquid enrichment. Have comparative studies on sensitivity been carried out? What was the rationale to use solid media enrichment?

7. In the abstract the authors state that CRISPR-BEEP offers improved sensitivity to conventional culture-based plate inspection technique. However, its sensitivity was slightly lower than the tested PCR-based method (101 positive water samples compared to 108 water samples). Although this difference is minor, the authors should include this information in the abstract and also add a sentence on explaining why CRISPR-BEEPs will be of value compared to established PCR-based methods.

8. Since soil is one of the most relevant habitats of B. pseudomallei, and since soil is especially challenging for PCR-based detection of B. pseudomallei, it would be very useful to evaluate CRISPR-BEEPs for this matrix. Did the authors evaluate their method on soil samples? This aspect should at least be addressed as a perspective in the discussion section of the manuscript.

9. In Line 362-365 the authors comment on the value of their method for resource-limited settings. However, it would be important to comment on the cost-efficiency of CRISPR-Beeps in comparison to other highly sensitive methods (e.g. PCR).

10. Starting in line 253 authors report on improved environmental detection of B. pseudomallei from water samples. However, it appears that qPCR and not CRISPR-Beeps was used for this analysis (?). The authors should clarify why CRISPR-Beep was not used for this part of the study?

Reviewer #2

(Remarks to the Author)
I co-reviewed this manuscript with one of the reviewers who provided the listed reports. This is part of the Nature Communications initiative to facilitate training in peer review and to provide appropriate recognition for Early Career Researchers who co-review manuscripts.

Reviewer #3

(Remarks to the Author)
Many thanks for the opportunity to review this paper. Please see review attached.

Version 1:

Reviewer comments:

Reviewer #1

(Remarks to the Author)
The authors have thoroughly addressed all points, conducted additional experiments, and integrated the new data into the revised manuscript. As a result, the manuscript has gained further in quality.

Reviewer #2

(Remarks to the Author)
I co-reviewed this manuscript with one of the reviewers who provided the listed reports. This is part of the Nature Communications initiative to facilitate training in peer review and to provide appropriate recognition for Early Career

Researchers who co-review manuscripts.

Reviewer #3

(Remarks to the Author)
Summary

Many thanks for the opportunity to re-review this important paper. This manuscript describes the development and field application of CRISPR-BEEPs, a CRISPR-Cas12-based workflow for environmental detection of Burkholderia pseudomallei. By integrating selective culture enrichment with plate-sweep DNA extraction and CRISPR-based detection, the authors present an approach that substantially improves sensitivity compared with conventional culture-based screening and performs comparably to a double-qPCR gold standard.

Application of this method across a large sampling region in northeast Thailand demonstrates widespread environmental detection of B. pseudomallei in multiple water sources and identifies an association between environmental detection and melioidosis risk at the community scale. Overall, the study addresses an important methodological challenge in environmental surveillance of B. pseudomallei and provides a potentially useful tool for field-based detection in endemic regions and indeed in regions where melioidosis endemicity is being investigated.

General Assessment

The revised manuscript has improved following peer review. The authors have clarified the methodological workflow and moderated several interpretations regarding environmental transmission and sanitation risk.

The combination of culture selection / enrichment with CRISPR-based detection represents a thoughtful and practical approach to improving environmental detection sensitivity while maintaining the ability to detect viable organisms. The integration of methodological validation with epidemiological analysis is also a strength of the study.

Overall, the manuscript is clearly written and the conclusions are broadly supported by the data. The work will be of interest to researchers studying melioidosis, environmental pathogen surveillance, and CRISPR-based diagnostics.

Minor Comments

Interpretation of environmental detection

The manuscript reports an association between environmental detection of B. pseudomallei and melioidosis risk. While this is an important finding, the discussion could more clearly emphasise that this represents an ecological association rather than direct evidence of transmission from specific sampled water sources.

Environmental sampling coverage

Environmental sampling was conducted for a subset of participants within the larger cohort. A brief clarification regarding how representative the sampling density is relative to environmental heterogeneity in the study region would strengthen interpretation of the spatial analysis.

Scope of the method

The current workflow is limited to water samples. Given that soil is widely recognised as an important environmental reservoir for B. pseudomallei, the discussion could briefly comment on whether similar approaches might be adapted for soil-based surveillance in future studies.

Terminology

The manuscript alternates between the terms CRISPR-BP34 and CRISPR-BEEPs. Maintaining consistent terminology throughout the manuscript would improve clarity.

Minor editorial clarity

A small number of sentences in the introduction and discussion could be shortened for readability, although these are minor stylistic issues.

Recommendation

This study presents a technically innovative and well-executed approach for improving environmental detection of Burkholderia pseudomallei. The revised manuscript has addressed the major concerns raised during review, and the remaining issues are minor clarifications.

I recommend acceptance after consideration of minor revision.

Open Access This Peer Review File is licensed under a Creative Commons Attribution 4.0 International License, which permits use, sharing, adaptation, distribution and reproduction in any medium or format, as long as you give appropriate credit to the original author(s) and the source, provide a link to the Creative Commons license, and indicate if changes were made.
In cases where reviewers are anonymous, credit should be given to 'Anonymous Referee' and the source.
The images or other third party material in this Peer Review File are included in the article's Creative Commons license, unless indicated otherwise in a credit line to the material. If material is not included in the article's Creative Commons license and your intended use is not permitted by statutory regulation or exceeds the permitted use, you will need to obtain permission directly from the copyright holder.
To view a copy of this license, visit https://creativecommons.org/licenses/by/4.0/

Our response is highlighted in grey

REVIEWER COMMENTS

Reviewer #1 (Remarks to the Author):

Reviewer Comments:

There are several studies reporting on melioidosis outbreaks with water as a source of infection. Accessible and sensitive methods for determining B. pseudomallei from environmental samples, including water are essential to eliminate potential infection sources and to raise awareness in endemic regions.

This well-written report by Pakdeerat addresses this topic and is of significance to the field. It demonstrates that the CRISPR-BEEPs method developed can detect B. pseudomallei colonies grown on a culture medium with significantly higher sensitivity than traditional visual inspection of colonies. Using this new methodology, the authors conducted a thorough and comprehensive study, proving that melioidosis cases occur significantly more frequently in households where B. pseudomallei has been detected in nearby water samples. This study will not only inform health policies to strengthen water sanitation but also provides a novel method to detect B. pseudomallei in water samples with improved sensitivity and specificity.

Consideration of the following points may help to further refine the evaluation of the CRISPR-based culture identification method in comparison to other methodological approaches.

We are immensely grateful to the reviewer for their constructive comments, which have substantially strengthened this manuscript. Their emphasis on distinguishing viable from non-viable cells aligns with concerns raised in our discussion with the Thailand Waterworks Authority, where DNA-based detection may represent residual DNA following chlorination. Guided by the reviewer's feedback, we have systematically evaluated three detection strategies – filtration alone, filtration followed by liquid culture, and filtration followed by solid plate culture – under both chlorine treated and untreated conditions (**Figure 2e**).

Our new experiments show that qPCR and CRISPR assays performed on directly filtered samples generate positive signals in both chlorinated and non-chlorinated water (**Figure 3a**), whereas filtration followed by culture distinguishes viable cells, with qPCR and CRISPR signals detected only in untreated samples and not in chlorine-treated samples (**Figure 3b & Supplementary Fig. 3**). This new experiment confirms that our analyses specifically target viable bacteria and strengthen the conclusion that the presence of viable *B. pseudomallei* in piped water represents a genuine public health concern. Together, this provides stronger evidence supporting water system interventions. We sincerely thank the reviewer for these insights. All comments are addressed below.

Specific comments

1. The CRISPR-BEEPs method is primarily a culture method in which, after filtration of water samples, the filters are inoculated onto Ashdown medium and the growth of B. pseudomallei is detected more quickly and sensitively than by visual inspection of the culture plates. Compared to direct molecular detection, culture remains the time-determining step here. This limitation should be addressed, especially in outbreak situations.

We thank the reviewer for this comment. We acknowledge that the culture step in CRISPR-BEEPs introduces a time delay relative to direct molecular detection, which may limit rapid outbreak response. However, this step is essential for distinguishing viable from non-viable *B. pseudomallei* as suggested by our new data (new **Figure 2e** and **Figure 3**). This is important for water treatment assessment, as residual DNA from dead cells can persist and lead to false-positive molecular signals, potentially triggering unnecessary public-health alerts. We now clarify this rationale (lines 199-205) and its time limitation (lines 422-428).

2. It would be interesting to validate the performance of their CRISPR method in comparison to PCR for the direct detection of B pseudomallei DNA from water samples. Various e. g. mechanical methods could be used to recover B. pseudomallei from the filter after filtration of the water samples and test it directly without incubation. One would expect the detection limit of the CRISPR and PCR methods to be reached under such conditions. This would guarantee more timely results and initiation of countermeasures and may also circumvent fungal overgrowth observed in 21 of the tested samples. This potential advantage of direct testing should be discussed.

We really appreciate this suggestion and have conducted additional experiments to assess the feasibility of direct detection of *B. pseudomallei* from water samples using filtration alone, and the filtration followed by culture. As our study included both piped and natural water sources, we evaluated two conditions: chlorine-treated water (to mimic piped water) and untreated water (to mimic natural sources), as schematically illustrated in new **Figure 2e**.

Due to biosafety constraints associated with processing large volumes of *B. pseudomallei*, we used *E. coli* engineered to carry the *B. pseudomallei* DNA target (*E. coli-Bp*) as a surrogate organism. We confirmed that this surrogate shows comparable analytical sensitivity to *B. pseudomallei* (**Figure 2a & b,** new **Supplementary Figure 2**). To simulate environmental conditions, we performed spiking experiments in which *E. coli-Bp* was added to PBS at final concentrations ranging from 4 to 100,000 CFU, consistent with reported environmental levels of *B. pseudomallei* in typically collected water volumes of 0.5 - 5 L. One set of samples was treated with chlorine to mimic standard water treatment practices, while the other set was left untreated. After filtration, both sets were tested directly using qPCR and CRISPR assays. Although chlorine treatment effectively killed *E. coli-Bp* across all tested concentrations (new **Figure 3c**), direct testing of filter papers produced positive qPCR and CRISPR signals in both chlorine-treated and untreated samples (new **Figure 3a**). Positive signals were consistently detected at ≥ 400 CFU in untreated samples and 100,000 CFU in chlorine-treated samples, indicating that residual DNA from non-viable cells remains detectable after chlorination.

These results show that while direct detection after filtration can reach the analytical limit of detection and offers speed advantages, it cannot distinguish viable from non-viable cells and may lead to false-positive results and unnecessary public-health alerts. We therefore conclude that, despite the additional time required, a culture step remains essential for biologically meaningful and actionable surveillance. This trade-off between speed and biologically relevance is now discussed in the revised manuscript (lines 422-428).

3. In line 369 to 374 the authors state that the culture step provides a more biologically relevant detection threshold, as it detects viable organisms and not just DNA. Can the authors provide data to support the assumption that the detection of viable cells is more disease-relevant compared to a direct semi-quantitative determination of B. pseudomallei DNA?

We thank the reviewer for raising this important point. The disease-relevance of nucleic acid-based environmental detection depends strongly on pathogen biology. For obligate intracellular pathogens such as SARS-CoV2, environmental RNA is relatively unstable and may reflects recent or ongoing infection, making semi-quantitative nucleic-acid detection clinically informative. In contrast, *B. pseudomallei* is a free-living environmental bacterium that persists in water and soil, and its DNA can remain detectable after cells are dead or inactivated, including following chlorination. Evidence from animal infection models demonstrates that melioidosis requires exposure to live *B. pseudomallei* capable of intracellular replication. Heat-killed or paraformaldehyde-killed *B. pseudomallei* (PMID:19490962, PMID:25223269), as well as purified *B. pseudomallei* DNA (PMID:16169637), do not cause infection in mice, although they may induce partial immune responses. Notably, lethal dose can occur with doses as low as 5-10 CFU, underscoring the central role of viable bacteria in disease causation and the need for highly sensitive detection of viable cells.

We now provide direct experimental evidence supporting this biological distinction. Direct detection after filtration generated positive qPCR and CRISPR signals even when viable cells were completely inactivated by chlorination (new **Figure 3a**). In contrast, inclusion of culture step selectively enriched viable bacteria, as demonstrated by increasing CFU counts over time (**Figure 3c**), which corresponded to increasing qPCR and CRISPR signals and enabled sensitive detection at starting concentrations as low as 4 CFU (**Figure 3b**, **Supplementary Figure 3**).

From a public health perspective, including our discussion with the Thailand Waterworks Authority, intervention decisions such as modifying water treatment practices require evidence of viable pathogens. DNA-only signals may reflect historical contamination or successful disinfection and can therefore trigger false-positive risk assessments and unnecessary public health responses. While semi-quantitative DNA-based detection can be faster and analytically sensitive, it cannot discriminate between viable and non-viable cells. We now explicitly discuss this trade-off between speed and biological relevance in the revised manuscript (lines 422-428)

-Do the authors have data showing that a positive CRISPR reaction of plates that were negative by conventional inspection is indeed due to proliferated cells? Comparisons of plate sweeps on day 0 and at later time points after incubation would be important here to show that positive signals were not due to B pseudomallei DNA that was simply washed off the filter, but are the results of bacterial growth.

We appreciate this important concern and performed additional experiments to address it. To determine whether positive CRISPR signals after culture reflect bacterial proliferation rather than residual *B. pseudomallei* DNA washed off the filter, we conducted time-course growth experiments in which filtered papers from both chlorine-treated and untreated samples were incubated in broth and on agar plates. This approached enabled quantitative enumeration of viable cells remaining on, and proliferating from, the filter over time. For biosafety reasons, *E. coli-Bp* was used as a surrogate organism, with sampling time points adjusted for its different growth rate.

Under chlorine-treated conditions, where only dead cells and residual DNA were present on the filter, no viable cells were detected at any time point (100 µL of incubated broth plated hourly and at 24 hours). Consistently, both qPCR and CRISPR assays on broth and agar cultures remained negative, indicating that DNA wash-off alone does not generate positive molecular signals. In contrast, under untreated conditions containing viable *E. coli-Bp*, CFU counts increased over time, consistent with bacterial proliferation. This increase closely paralleled the intensification of qPCR and CRISPR signals, which peaked at 24 hours in both broth and agar plate (new **Figure 3b, c**).

Collectively, these results confirm that positive CRISPR signals observed after culture arise from actively proliferating cells rather than residual DNA carried over from filtration.

-Along the same line, did the authors attempt to isolate strains from CRISPR and/or PCR-positive but inspection-negative plate sweeps through additional plating of diluted sweeps?

We agree with the reviewer that overcrowded growth could have obscured colony visualisation and that additional dilution could have rescued these samples. Unfortunately, in the original study, we did not perform serial dilutions at the time of sampling due to the unknown concentration of *B. pseudomallei* in natural water and noted this as one of the limitations (lines 87-89)

- How long were the filters incubated on Ashdown agar before colonies were harvested by sweeping the plates?

For *B. pseudomallei*, the filters were incubated on Ashdown agar for 3 days before sweeping. We have now included this information in the revised manuscript (line 600).

4. In Line 205-208 the authors state that most environmental water samples are likely to contain B. pseudomallei at a concentration below the direct detection limits of current molecular assays and therefore suggest pre-enrichment by proliferation on agar. In this context the filtration step performed should also be mentioned, as it represents a key enrichment step and could enable culture-independent detection using the CRISPR method. The detection limit of their CRISPR method should be discussed in the context of PCR-based methods, including references.

We thank the reviewer for this comment and agree that filtration is a critical enrichment step. We have now performed additional experiments in which qPCR and CRISPR assays were applied directly following filtration (new **Figure 3a**). These results show that filtration improves analytical sensitivity and enables rapid molecular detection. However, filtration alone cannot distinguish viable from non-viable cells, as DNA from inactivated bacteria remains detectable after chlorination. As this distinction is essential for public-health risk assessment in endemic settings, the culture step remains necessary both to further increase bacterial concentration and to confirm viability. The detection limits of qPCR and CRISPR following direct filtration in both untreated and chlorinated samples are now shown in Figure 3a and discussed in the Results (lines 198-234), and Methods (lines 538-586)

5. The wording in lines 241 to 242, ‘In contrast to the conventional culture-based method,’ might be changed to ‘in contrast to the conventional culture identification method,’ as it is still a culture-based approach.

We thank the reviewer for this comment and have revised the text accordingly.

6. The authors should consider discussing existing studies that have detected B. pseudomallei, from water samples using PCR of liquid enrichment. Did the authors also used the CRISPR technology with liquid enrichment. Have comparative studies on sensitivity been carried out? What was the rationale to use solid media enrichment?

We thank the reviewer for this thoughtful comment. Using *E. coli-Bp* as a proxy, we conducted additional experiments comparing liquid (broth) and solid (agar) enrichment following filtration (**Figure 2e, 3b, Supplementary Figure 3**). Both approaches yielded comparable results: viable-cell samples became positive by qPCR and CRISPR at 24 hours, while non-viable samples remained negative. Given this similar sensitivity, we selected solid agar enrichment for practical and biosafety reasons. Handling large volumes of liquid potentially containing *B. pseudomallei* poses a spillage risk. In addition, solid agar plates require less space than equivalent volumes of liquid media, an important logistic consideration in our laboratory in northeast Thailand. This choice enables safer handling and higher throughput for field surveillance (lines 227-234).

7. In the abstract the authors state that CRISPR-BEEP offers improved sensitivity to conventional culture-based plate inspection technique. However, its sensitivity was slightly lower than the tested PCR-based method (101 positive water samples compared to 108 water samples). Although this difference is minor, the authors should include this information in the abstract and also add a sentence on explaining why CRISPR-BEEPs will be of value compared to established PCR-based methods.

We are grateful for this comment, which helped improve the clarity of our reporting. We have revised the abstract to explicitly state that PCR-based method was used as the reference standard; consequently, the reported sensitivity of CRISPR-BEEPs is slightly lower relative to this reference (lines 39-40), while epidemiological results obtained from CRISPR-BEEPs remain comparable to those from PCR-based methods (lines 44). Due to an abstract 150-word limit, we highlight the advantages of CRISPR-BEEPs over the established PCR-based methods in the discussion (lines 390-395)

8. Since soil is one of the most relevant habitats of B. pseudomallei, and since soil is especially challenging for PCR-based detection of B. pseudomallei, it would be very useful to evaluate CRISPR-BEEPs for this matrix. Did the authors evaluate their method on soil samples? This aspect should at least be addressed as a perspective in the discussion section of the manuscript.

We totally agree with the reviewer and have now addressed this in the discussion (lines 403-407). While soil is a key reservoir, its complexity and inhibitory substances make molecular detection challenging. Evaluating CRISPR-BEEPs for soil is a priority for future work, but was beyond the scope of this study due to resource and technical constraints.

9. In Line 362-365 the authors comment on the value of their method for resource-limited settings. However, it would be important to comment on the cost-efficiency of CRISPR-Beeps in comparison to other highly sensitive methods (e.g. PCR).

We thank the reviewer for this important point. We have now expanded the manuscript to compare the cost-efficiency and practical implementation of CRISPR-BEEPs with the standard PCR-based methods, with a particular focus on resource-limited settings. This comparison is framed using WHO REASSURED criteria and is summarised in **Supplementary Table 16-17** in the **Supplementary Information**.

10. Starting in line 253 authors report on improved environmental detection of B. pseudomallei from water samples. However, it appears that qPCR and not CRISPR-Beeps was used for this analysis (?). The authors should clarify why CRISPR-Beep was not used for this part of the

study?

We apologise for the omission and thank the reviewers for highlighting this point. We have now included both qPCR and CRISPR-BEEPs results throughout the epidemiological analysis (lines 271-281, 297-308, 325-338 and 358-371; new **Figure 4, new Supplementary Table 2 & 3**). The two methods show comparable detection patterns and both reveal a significant association between environmental detection and melioidosis risk, whereas conventional plate inspection does not. Including CRISPR-BEEPs in this analysis strengthens our conclusions and clarifies its value over standard culture-based inspection.

Reviewer #2 (Remarks to the Author):

I co-reviewed this manuscript with one of the reviewers who provided the listed reports. This is part of the Nature Communications initiative to facilitate training in peer review and to provide appropriate recognition for Early Career Researchers who co-review manuscripts.

We are so grateful for the thoughtful comments provided. They have really helped strengthen our manuscript. We also appreciate the journal's efforts to foster training in the peer review process.

Reviewer #3 (Remarks to the Author):

Many thanks for the opportunity to review this paper. Please see review attached.
Key results

-The key result is the extent and epidemiological associations with Bps detection in water sources in an endemic region in Thailand.

-These associations were enabled by the development and validation of highly sensitive and specific molecular tools including a novel, for Bps environmental detection, CRISPR-based technique

-The paper sets a standard of how molecular techniques for Bps detection are validated and used to improve environmental surveillance and more broadly, melioidosis epidemiology

-The study avoids the issue of molecular tools detection (and therefore discussion of) detection of non-viable Bps genomes but DNA extracting not directly from environment samples but from the results of colonial growth of filtered samples from ASH selective agar

We sincerely thank the reviewer for their careful, thorough, and constructive review. Their comments prompted reanalysis of the surveillance and epidemiological sections, strengthening the robustness of our study, and overall improved the clarity and reproducibility of our work. We also appreciate the reviewer's recognition of our original detection approach – filtration followed by culture – which helps reduce false-positive signals from non-viable *B. pseudomallei*, which is particularly important for treated water sources where dead cells may persist. All suggestions have been carefully considered and incorporated in the revised manuscript.

Validity

-The study it excellent and important, only some minor suggestions of improvement, see below

-The development and validation of molecular tools for the detection of environmental Bps is important so to better understand the epidemiology of the disease

-The paper makes good progress however, because the molecular tools are used on cultivated samples, the assays are really being used to confirm a culture result rather test a sample directing. This means that communities where the assays are deployed still need to have access to a PC2 (at least lab) with skilled scientists, so even those the detection limit is significantly higher, infrastructure is stilled required.

-However, being used on cultured samples at least limits a discussion on a major issue of Bps environmental molecular detection and that is, the significance os viable vs non-viable

We thank the reviewer for recognising the validity and importance of our study. We agree that distinguishing between viable and non-viable *B. pseudomallei* is critical for environmental detection, particularly in treated water systems. To demonstrate this, we performed additional experiments assessing direct molecular detection from filter papers without culture, using *E. coli* engineered to carry the *B. pseudomallei* DNA target (*E. coli-Bp*) as a surrogate to meet biosafety requirements. We confirmed that this surrogate shows comparable analytical sensitivity to *B. pseudomallei* (**Figure 2a & b,** new **Supplementary Figure 2**). Our new results showed that direct, culture-independent detection from filters cannot distinguish viable from non-viable cells (new **Figure 3**), confirming that a culture step remains necessary for biologically and public-health-relevant detection. We acknowledge that inclusion of a culture step requires basic laboratory infrastructure, including BSL2 facilities and formally compared the utility CRISPR-BEEPs with standard qPCR using the WHO REASSURED framework, outlining the advantages, limitations, and deployment settings for each approach (**Supplementary Tables 16 & 17**).

Data and methodology

-Development, validation and use of the molecular assay methods is excellent

-Some subtle suggestions, for the description of the sampling and DNA extraction methods, see below

Analytical approach:

Stats look fine

Suggested improvements:

Please see below.

Clarity and context:

Paper is very well written

References:

Appropriate and thorough.

We appreciate the reviewer's careful assessment of our data, methodology, analytical approach, clarity of writing, and references. We are pleased that these aspects were well received.

Notes:

Abstract

-The title and abstract describes the use of CRISPR vs culture but doesn't include the use of double qPCRs which were really the gold standard assay used in the subsequent surveillance work.

We thank the reviewer and apologise for this oversight. We have evised the abstract to explicitly state the use of double-qPCR as a reference standard (lines 39-40), and have updated the surveillance description to focus on CRISPR-BEEPs, clarifying that the results are comparable to double-qPCR (line 44).

-Perhaps the major novelty if the CRISPR development and validation, however the results of the surveillance work and the subsequent epidemiological analysis could and was also determined my the double PCR technique from cultured samples

We thank the reviewer for raising this important point and apologise for the previous omission. We have now incorporated results from double-qPCR, CRISPR-BEEPs, and conventional plate inspection throughout the revised manuscript (lines 271-281, 297-308, 325-338 and 358-371; new **Figure 4**; and new **Supplementary Table 2 & 3**). These analyses show that double-qPCR and CRISPR-BEEPs yield comparable detection patterns and both reveal a significant association between environmental detection and melioidosis risk. In contrast, conventional plate inspection alone does not show this association, highlighting the added value of CRISPR-BEEPs over conventional plate inspection. The abstract has been revised to explicitly highlight the epidemiological findings obtained using CRISPR-BEEPs.

- 62-64: Reported case fatality rates typical in region where the health systems are not able to support adequate management; the disease is adequately reported in some regions where there is a disease surveillance networks, this and other issues of HS support reduce the CFT to less than 10%. Do the authors want to better contextualise these comments to regions with non-first world health systems?

We agree with the reviewer and have added text to better contextualised the reported case fatality rate, highlighting differences between regions with well-resourced health systems and those with limited healthcare infrastructure (lines 61-63).

- 73-76: contaminated aromatherapy is arguably more an QA preparation issue rather than linked issue of water sanitation, perhaps?

We agree with the reviewer and have removed this example from the Introduction.

- 89: or PCR from suspected colonies

We have amended the text as suggested (lines 84)

- 91: perhaps best to make it clear why AMR important here – "resistance to the antibiotics commonly used in media to aid in culture selection, such as gentamicin and colistin"

We thank the reviewer for this suggestion and have expanded the text for clarification (lines 85-87)

Results

Method development

- 128-137: excellent approach of specificity optimisation / validation, however the regions where samples have been collected and reported in the references MAG paper, do not seem to include melioidosis endemic regions in NT Australia, so therefore, the validation of the assay cant necessarily represent a melioidosis endemic globally relevance? Probably worth thinking about including this issue in the discussion / limitations

We agree with the reviewer and have added this limitation to the manuscript, noting that the reference MAG dataset does not fully represent all melioidosis-endemic regions (lines 131-134).

- 146: Perhaps just mention the “BCC and BPS complexes” or just state “members of the Burkholderiales order”

We thank the reviewer for this comment and have revised the text accordingly (lines 144).

- 151: very likely sequencing depth not sufficient in the MAG data set to ID spp with the Burkholderiales? Interesting issue of Bps low abundance but also potentially bacteria biogeography at play in regards to Bps – the MAG set is a result of isolated environmental sampling and prone to not represent non-randomly distributed bacterial populations accurately - see above comments

We agree with the reviewer and acknowledge the limitations of the MAG dataset, including insufficient sequencing read depth and potential biogeographical biases that may hinder detection of *B. pseudomallei*. We have now highlighted this issue in the manuscript (lines 146-149)

- 229 - 240: so double qPCR from DNA scaped off plates 108/335, colonial observation 23/335, CRISPR-BEEPs 101/108 PCR confirmed – the assay that then has been relied upon is the double qPCR rather than the CRISPR method, the title of the paper infers that the main outcome of the paper is the use of a novel CRISPR assay when perhaps the CRISPR assay has been validated during a water surveillance program? Perhaps the title of the paper should reflect that and/or consider a small methods paper independent of this paper to describe the CRISPR technique.

We apologise for the omission and thank the reviewer for highlighting this point. We have now included qPCR, CRISPR-BEEPs and conventional plate inspection results throughout surveillance and epidemiological analysis (lines 271-281, 297-308, 325-338 and 358-371; **new Figure 4, new Supplementary Table 2 & 3**). The qPCR and CRISPR-BEEPs show comparable detection patterns and both reveal a significant association between environmental detection and melioidosis risk, whereas conventional plate inspection does not. Including CRISPR-BEEPs in this analysis strengthens our conclusions and clarifies its value over standard culture-based inspection.

Surveillance

- 254-257: Using double qPCR, Bps detected in all types of water tested, highest in groundwater

- Subterranean bore water shielded from UV light and temperature fluctuations highest

– stable reservoirs

-269: **Seasonality** - Surface water in rainy season - 39% surface water in rainy season pos, none in the dry season (idea – test seasonal creeks across seasons) – trend also seen in pipped water (5.3% in dry season, 45% in wet season). “High resolution year round data, confirm earlier multi-observations year. “wet season condition substantially increase the environmental burden of Bps and likely elevate the exposure risk - good

- Very nice data

We thank the reviewer for their thorough review and support of the findings.

Exposure and risk

- 295-308 Melioidosis patients communities have a higher rate of environmental positivity but only just, good that the reality of host factors are included and discussed factors important

We are pleased that the reviewer appreciated the inclusion of host factors in the analysis.

Combined effect of environment and host

-330 - Female and being overweight likely reflect reduced high exposure occupation compared to lean male farm workers – good

- 340 – 350 - PCR based detection demonstrated significant associations with environmental detection and melioid incidence, direct culture didn’t – increased sensitivity important – this makes sense and also makes sense that once validated CRISPR more sensitive that culture observation, unsurprising.

We are delighted that the reviewer agrees with the findings and conclusions.

Discussion

- To balance sens and biological activity the sweep method limited the issues of non-viable bacteria - so no need to discuss the issue of the potential clinical significance of non-viable Bps n this paper, but probably useful to speak about (pros and cons) of this technique vs direct testing of environemntal samples

We fully agree with the reviewer. To address this point, and in response to another reviewer’s request, we perform additional experiments comparing direct molecular detection after filtration within detection following filtration and culture. These experiments highlight the necessity of a culture step in distinguishing viable from non-viable cells. We now discuss the advantages and limitations of these approaches in the revised manuscripts (lines 422-428)

- CRISR might be promising but its use in this paper is not the paper’s main output

We apologise for the earlier omission of CRISPR-BEEPs results from the surveillance and epidemiological sections, which may have given the impression that CRISPR-BEEPs was not a central output of the study. We have now reanalysed the data and revised the manuscript to fully

incorporate CRISPR-BEEPs into the main results and conclusions (lines 271-281, 297-308, 325-338 and 358-371).

- It is inferred that the CRISPR technique would be more sustainable in regional endemic communities that qPCR “equipment light” – is there any attempt at a cost comparison to support this? Would not labs that have the capacity to culture Bps also have a general capacity to do PCR?

We thank the reviewer for raising this point. Our data show that a culture step remains essential to distinguish viable from non-viable B. *pseudomallei*, particularly in endemic settings where residual DNA can persist after disinfection (new Figure 3); therefore, basic culture capacity is a minimum requirement for any approach. Following culture, CRISPR-BEEPs offers advantages over qPCR, as it is equipment-light, does not require a thermocycler, and is more tolerant of environmental inhibitors that may affect PCR performance and necessitate extensive troubleshooting. We now explicitly discuss these practical differences and include a cost and feasibility comparison with qPCR based on WHO REASSURED criteria (lines 390-395, **Supplementary Table 16-17**)

- Double PCR method still can’t completely rule out non-Bps detection or primers unable to detected novel Bps strains, particular if the primers validated against nonBps from the MAG data and not focused on melioidosis endemic regions.

We agree with the reviewer and acknowledge this limitation. No single primer set can completely exclude cross-reactivity or guarantee detection of all novel *B. pseudomallei* strains given incomplete representation of endemic regions in available MAG datasets. We now explicitly note this limitation and emphasise that combining culture with molecular confirmation mitigates, but not entirely eliminate this risk (lines 428-433)

M&M

- 448 – samples weren’t enriched as such, ASH is a selective agar, allowing Bps to

replicate in the presence of inhibitors towards other microbes

We have reworded this accordingly in the manuscript (lines 598-599).

- What volume was filtered? All 5 litres or just 1 litre?

We apologise for this oversight and have expanded the water sampling protocol and the volume rationale in the manuscript (lines 590-597)

- Plates incubated at 40 degrees should limit fungi

We initially had the same expectation, but fungal growth was still observed at 40C. This potentially suggests the presence of heat-tolerant fungi, which may warrant further investigation in future studies.

- Bacterial DNA extraction - For molecular testing, a sweep from the plate was preserved n 20% glycerol stock and stored at -80

We envision that DNA extraction could be performed either directly from plates or from preserved samples. Due to limited manpower on site, we chose to preserve the samples for later DNA extraction.

- Glycerol stock was cultured – how much was sub cultured? “a loop full of colonies was used for bacterial DNA extraction…” So, was the resulted individual colonies of a sweep from plate or a resweep?? – please explain this a bit more clearly

We thank the reviewer for highlighting this point and for the opportunity to clarify our workflow. Due to limited manpower at the study site during the water sampling period, DNA extraction could not be performed directly from the initial plate sweeps. Therefore, plate-sweep material from each sample was preserved as 1 mL glycerol stock for later processing. For DNA extraction, a 20 µL aliquot from each glycerol stock was plated onto Ashdown agar and incubated. After growth, the entire plate was re-swept to collect the mixed bacterial population, and this re-sweep material was used for DNA extraction. These details have now been added to the Methods section (lines 600-603 and 616-620)

-The diagram in figure doesn’t represent the described method accurately as the glycerol stock component is not included.

We thank the reviewer for this comment. The figure is intended to illustrate the core analytical workflow rather than intermediate sample preservation steps. The glycerol stock represents a temporary storage step introduced for logistical reasons and does not change the underlying method shown in the schematic. We have clarified this in the figure legend (lines 959-961) and Methods section (lines 616-620) to avoid confusion.

Our response is highlighted in grey.

REVIEWERS' COMMENTS

Reviewer #1 (Remarks to the Author):

The authors have thoroughly addressed all points, conducted additional experiments, and integrated the new data into the revised manuscript. As a result, the manuscript has gained further in quality.

We are incredibly grateful for reviewer feedback, which has been a huge help in strengthening our work.

Reviewer #2 (Remarks to the Author):

I co-reviewed this manuscript with one of the reviewers who provided the listed reports. This is part of the Nature Communications initiative to facilitate training in peer review and to provide appropriate recognition for Early Career Researchers who co-review manuscripts.

We thank the reviewer for constructive feedback, and especially for including an early career researcher in the review process. It is really inspiring and it encourages us to do the same when we can.

Reviewer #3 (Remarks to the Author):

Summary

Many thanks for the opportunity to re-review this important paper. This manuscript describes the development and field application of CRISPR-BEEPs, a CRISPR-Cas12-based workflow for environmental detection of Burkholderia pseudomallei. By integrating selective culture enrichment with plate-sweep DNA extraction and CRISPR-based detection, the authors present an approach that substantially improves sensitivity compared with conventional culture-based screening and performs comparably to a double-qPCR gold standard.

Application of this method across a large sampling region in northeast Thailand demonstrates widespread environmental detection of B. pseudomallei in multiple water sources and identifies an association between environmental detection and melioidosis risk at the community scale. Overall, the study addresses an important methodological challenge in environmental surveillance of B. pseudomallei and provides a potentially useful tool for field-based detection in endemic regions and indeed in regions where melioidosis endemicity is being investigated.

General Assessment

The revised manuscript has improved following peer review. The authors have clarified the methodological workflow and moderated several interpretations regarding environmental transmission and sanitation risk.

The combination of culture selection / enrichment with CRISPR-based detection represents a thoughtful and practical approach to improving environmental detection sensitivity while maintaining the ability to detect viable organisms. The integration of methodological validation with epidemiological analysis is also a strength of the study.

Overall, the manuscript is clearly written and the conclusions are broadly supported by the data. The work will be of interest to researchers studying melioidosis, environmental pathogen surveillance, and CRISPR-based diagnostics.

We are deeply grateful to the reviewer for the thorough and constructive comments, which have helped strengthen not only this manuscript but also reflect the supportive spirit of the melioidosis community. We are thankful for the opportunity to contribute to these shared efforts to reduce melioidosis risk.

Minor Comments

Interpretation of environmental detection

The manuscript reports an association between environmental detection of B. pseudomallei and melioidosis risk. While this is an important finding, the discussion could more clearly emphasise

that this represents an ecological association rather than direct evidence of transmission from specific sampled water sources.

We thank the reviewer for this important point. We have revised the discussion to clarify that the relationship between environmental detection and melioidosis risk represents an ecological association. The text has been updated to emphasise this interpretation while noting that the findings still provide an important evidence base for urgent public health intervention (lines 446, 448-450).

Environmental sampling coverage

Environmental sampling was conducted for a subset of participants within the larger cohort. A brief clarification regarding how representative the sampling density is relative to environmental heterogeneity in the study region would strengthen interpretation of the spatial analysis.

We thank the reviewer for this suggestion. Environmental sampling was conducted in a subset of participants within the larger cohort to enable detailed household-level investigation while remaining logistically feasible across a large endemic region. The sampling framework captured some environmental heterogeneity, including water sources and hydrological conditions (for example, flooding-prone area vs dry areas). Sampling framework was designed to capture spatial variation in exposure at the scale relevant to individual risk and correlate with population density by the district (figure 6c). We have now clarified this in the Methods and Discussion to emphasise the scope of the sampling framework (lines 467-469 and 487-489).

Scope of the method

The current workflow is limited to water samples. Given that soil is widely recognised as an important environmental reservoir for B. pseudomallei, the discussion could briefly comment on whether similar approaches might be adapted for soil-based surveillance in future studies.

We thank the reviewer and have added a statement in the Discussion noting that adapting CRISPR-based approaches for soil would likely require a soil-specific extraction workflow and represents an important direction for future methodological development (lines 407-408).

Terminology

The manuscript alternates between the terms CRISPR-BP34 and CRISPR-BEEPs. Maintaining consistent terminology throughout the manuscript would improve clarity.

The manuscript has been revised to ensure consistent use of term CRISPR-BEEPs throughout the text to improve clarity.

Minor editorial clarity

A small number of sentences in the introduction and discussion could be shortened for readability, although these are minor stylistic issues.

The Introduction and Discussion have been edited to improve clarity and readability as suggested.

Recommendation

This study presents a technically innovative and well-executed approach for improving environmental detection of Burkholderia pseudomallei. The revised manuscript has addressed the major concerns raised during review, and the remaining issues are minor clarifications.

I recommend acceptance after consideration of minor revision.

We are very grateful to the reviewer for the constructive feedback throughout the review process. We have addressed the minor clarifications raised and revised the manuscript accordingly.

Key results

- The key result is the extent and epidemiological associations with Bps detection in water sources in an endemic region in Thailand.
- These associations were enabled by the development and` validation of highly sensitive and specific molecular tools including a novel, for Bps environmental detection, CRISPR-based technique
- The paper sets a standard of how molecular techniques for Bps detection are validated and used to improve environmental surveillance and more broadly, melioidosis epidemiology
- The study avoids the issue of molecular tools detection (and therefore discussion of) detection of non-viable Bps genomes but DNA extracting not directly from environment samples but from the results of colonial growth of filtered samples from ASH selective agar

Validity

- The study it excellent and important, only some minor suggestions of improvement, see below

Significance

- The development and validation of molecular tools for the detection of environmental Bps is important so to better understand the epidemiology of the disease
- The paper makes good progress however, because the molecular tools are used on cultivated samples, the assays are really being used to confirm a culture result rather test a sample directing. This means that communities where the assays are deployed still need to have access to a PC2 (at least lab) with skilled scientists, so even those the detection limit is significantly higher, infrastructure is stilled required.
- However, being used on cultured samples at least limits a discussion on a major issue of Bps environmental molecular detection and that is, the significance os viable vs non-viable

Data and methodology

- Development, validation and use of the molecular assay methods is excellent
- Some subtle suggestions, for the description of the sampling and DNA extraction methods, see below

Analytical approach

- Stats look fine

Suggested improvements

Please see below.

Clarity and context

Paper is very well written

References

Appropriate and thorough.

Notes:

Abstract

- The title and abstract describes the use of CRISPR vs culture but doesn't include the use of double qPCRs which were really the gold standard assay used in the subsequent surveillance work.
- Perhaps the major novelty if the CRISPR development and validation, however the results of the surveillance work and the subsequent epidemiological analysis could and was also determined my the double PCR technique from cultured samples

Introduction

- **62-64:** Reported case fatality rates typical in region where the health systems are not able to support adequate management; the disease is adequately reported in some regions where there is a disease surveillance networks, this and other issues of HS support reduce the CFT to less than 10%. Do the authors want to better contextualise these comments to regions with non-first world health systems?
- **73-76:** contaminated aromatherapy is arguably more an QA preparation issue rather than linked issue of water sanitation, perhaps?
- **89:** or PCR from suspected colonies
- **91:** perhaps best to make it clear why AMR important here – "resistance to the antibiotics commonly used in media to aid in culture selection, such as gentamicin and colistin"

Results

Method development

- **128-137:** excellent approach of specificity optimisation / validation, however the regions where samples have been collected and reported in the references MAG paper, do not seem to include melioidosis endemic regions in NT Australia, so therefore, the validation of the assay cant necessarily represent a melioidosis endemic globally relevance? Probably worth thinking about including this issue in the discussion / limitations
- **146:** Perhaps just mention the "BCC and BPS complexes" or just state "members of the Burkholderiales order"
- **151:** very likely sequencing depth not sufficient in the MAG data set to ID spp with the Burkholderiales? Interesting issue of Bps low abundance but also potentially bacteria biogeography at play in regards to Bps – the MAG set is a result of isolated environmental sampling and prone to not represent non-randomly distributed bacterial populations accurately - see above comments
- **229 - 240:** so double qPCR from DNA scaped off plates 108/335, colonial observation 23/335, CRISPR-BEEPs 101/108 PCR confirmed – the assay that then has been relied upon is the double qPCR rather than the CRISPR method, the title of the paper infers that the main outcome of the paper is the use of a novel CRISPR assay when perhaps the CRISPR assay has been validated during a water surveillance program? Perhaps the title of the paper should reflect that and/or consider a small methods paper independent of this paper to describe the CRISPR technique.

Surveillance

- **254-257:** Using double qPCR, Bps detected in all types of water tested, highest in groundwater
- Subterranean bore water shielded from UV light and temperature fluctuations highest – stable reservoirs
- **269: Seasonality -** Surface water in rainy season - 39% surface water in rainy season pos, none in the dry season (idea – test seasonal creeks across seasons) – trend also seen in pipped water (5.3% in dry season, 45% in wet season). "High resolution year round data, confirm earlier multi- observations year. "wet season condition substantially increase the environmental burden of Bps and likely elevate the exposure risk - good
- Very nice data

Exposure and risk

- **295-308** Melioidosis patients communities have a higher rate of environmental positivity but only just, good that the reality of host factors are included and discussed factors important

Combined effect of environment and host

- **330 -** Female and being overweight likely reflect reduced high exposure occupation compared to lean male farm workers – good
- **340 – 350 -** PCR based detection demonstrated significant associations with environmental detection and melioid incidence, direct culture didn't - increased sensitivity important – this makes sense and also makes sense that once validated CRISPR more sensitive that culture observation, unsurprising.

Discussion

- To balance sens and biological activity the sweep method limited the issues of non-viable bacteria - so no need to discuss the issue of the potential clinical significance of non-viable Bps n this paper, but probably useful to speak about (pros and cons) of this technique vs direct testing of environemntal samples
- CRISR might be promising but its use in this paper is not the paper's main out put
- It is inferred that the CRISPR technique would be more sustainable in regional endemic communities that qPCR "equipment light" – is there any attempt at a cost comparison to support this? Would not labs that have the capacity to culture Bps also have a general capacity to do PCR?
- Double PCR method still can't completely rule out non-Bps detection or primers unable to detected novel Bps strains, particular if the primers validated against nonBps from the MAG data and not focused on melioidosis endemic regions.

M&M

- **448 –** samples weren't enriched as such, ASH is a selective agar, allowing Bps to replicate in the presence of inhibitors towards other microbes
- What volume was filtered? All 5 litres or just 1 litre?
- Plates incubated at 40 degrees should limit fungi
- **Bacterial DNA extraction -** For molecular testing, a sweep from the plate was preserved n 20% glycerol stock and stored at -80
- Glycerol stock was cultured – how much was sub cultured? "a loop full of colonies was used for bacterial DNA extraction…" So, was the resulted individual

colonies of a sweep from plate or a resweep?? – please explain this a bit more clearly

- The diagram in figure doesn't represent the described method accurately as the glycerol stock component is not included.
